# Facilitative lysosomal transport of bile acids alleviates ER stress in mouse hematopoietic precursors

**DOI:** 10.1038/s41467-021-21451-6

**Published:** 2021-02-23

**Authors:** Avinash K. Persaud, Sreenath Nair, Md Fazlur Rahman, Radhika Raj, Brenna Weadick, Debasis Nayak, Craig McElroy, Muruganandan Shanmugam, Sue Knoblaugh, Xiaolin Cheng, Rajgopal Govindarajan

**Affiliations:** 1 Division of Pharmaceutics & Pharmacology, College of Pharmacy, Ohio State University, Columbus, OH 43210 USA; 2 Depatment of Veterinary Biosciences, College of Veterinary Medicine, Ohio State University, Columbus, OH 43210 USA; 3 Division of Medicinal Chemistry & Pharmacognosy, College of Pharmacy, Ohio State University, Columbus, OH 43210 USA; 4 Translational Therapeutics, Ohio State University Comprehensive Cancer Center, Ohio State University, Columbus, OH 43210 USA

**Keywords:** Stress signalling, Lysosomes, Endoplasmic reticulum, Haematopoietic stem cells, Pathogenesis

## Abstract

Mutations in human equilibrative nucleoside transporter 3 (ENT3) encoded by *SLC29A3* results in anemia and erythroid hypoplasia, suggesting that ENT3 may regulate erythropoiesis. Here, we demonstrate that lysosomal ENT3 transport of taurine-conjugated bile acids (TBA) facilitates TBA chemical chaperone function and alleviates endoplasmic reticulum (ER) stress in expanding mouse hematopoietic stem and progenitor cells (HSPCs). *Slc29a3*^*−/−*^ HSPCs accumulate less TBA despite elevated levels of TBA in *Slc29a3*^*−/−*^ mouse plasma and have elevated basal ER stress, reactive oxygen species (ROS), and radiation-induced apoptosis. Reintroduction of ENT3 allows for increased accumulation of TBA into HSPCs, which results in TBA-mediated alleviation of ER stress and erythroid apoptosis. Transplanting TBA-preconditioned HSPCs expressing ENT3 into *Slc29a3*^*−/−*^ mice increase bone marrow repopulation capacity and erythroid pool size and prevent early mortalities. Together, these findings suggest a putative role for a facilitative lysosomal transporter in the bile acid regulation of ER stress in mouse HSPCs which may have implications in erythroid biology, the treatment of anemia observed in ENT3-mutated human genetic disorders, and nucleoside analog drug therapy.

## Abstract (web summary)

Mutations in ENT3, encoded by *SLC29A3*, result in anaemia and erythroid hypoplasia, suggesting roles in erythropoiesis. Here the authors show that ENT3 acts as a lysosomal bile acid transporter, and mutation compromises taurine conjugated bile acid transport in erythroid progenitors leading to ER stress, and anaemia.

## Introduction

*SLC29A3* resides in the long (q) arm of chromosome 10 at position 22.1 and encodes the intracellular equilibrative nucleoside transporter ENT3^1–3^. Mutations in *SLC29A3* cause an expanding spectrum of human monogenic diseases (H syndrome, Pigmented Hypertrichosis with Insulin-dependent Diabetes Mellitus (PHID) syndrome, Rosai Dorfman Disease (RDD), and Sinus Histiocytosis with Massive Lymphadenopathy (SHML), etc.) with anemia, erythroid hypoplasia, and red cell aplasia as accompanying features^4–12^. Mice deficient for the mouse orthologue of human ENT3 (mENT3) develop severe anemia and early mortalities, and through screening a mouse knockout library of 472 transmembrane proteins, it was discovered that *Slc29a3* (which encodes mENT3) is a critical gene in the development of RBCs^13,14^. However, the relationship between erythropoiesis and ENT3 and the molecular pathogenesis of anemia in *SLC29A3* mutation-driven disorders remains poorly understood.

We have previously determined that ENT3 deficiencies alter hematopoietic and mesenchymal stem cell fates in *Slc29a3*^−/−^ mice, which may underline the pathogenicity of ENT3-based genetic disorders^14^. We demonstrated that the loss of ENT3 depletes bone marrow cellularity and impairs self-renewal and differentiation properties of mouse hematopoietic and mesenchymal stem cells, and we identified that aberrant adenosine monophosphate kinase (AMPK) signaling contributes to the loss of stemness observed in *Slc29a3*^−/−^ adult stem cells^14^. Although stem cell transplantation and pharmacological AMPK activation both increased survival times, only stem cell transplantation extended long-term survival and fully recovered anemia in *Slc29a3*^−/−^ mice^14^. These findings suggest that additional stem cell-related mechanisms may be involved in the ENT3-regulation of erythropoiesis.

Here, by evaluating ENT3-null (*Slc29a3*^−/−^) mice and conducting mass spectrometry-based metabolomics and functional bone marrow reconstitution assays, we uncover a mechanism that allows ENT3 to regulate erythroid pool size. This mechanism incorporates ENT3-mediated lysosomal transport of bile acids (BA), newly discovered substrates of ENT3, which facilitates the chemical chaperone function of taurine-conjugated BA (TBA) to regulate ER stress in hematopoietic stem and progenitor cells (HSPC). Our findings expand the known substrate repertoire of ENT3 and demonstrate that the lysosomal retrograde transport of BA is critical for the regulation of ER stress and HSC homeostasis during mouse erythropoiesis.

## Results

### Reduction in bone marrow HSPCs and defects along the erythroid line of differentiation lead to anemia in *Slc29a3*^−/−^ mice

**Fig. 1:**
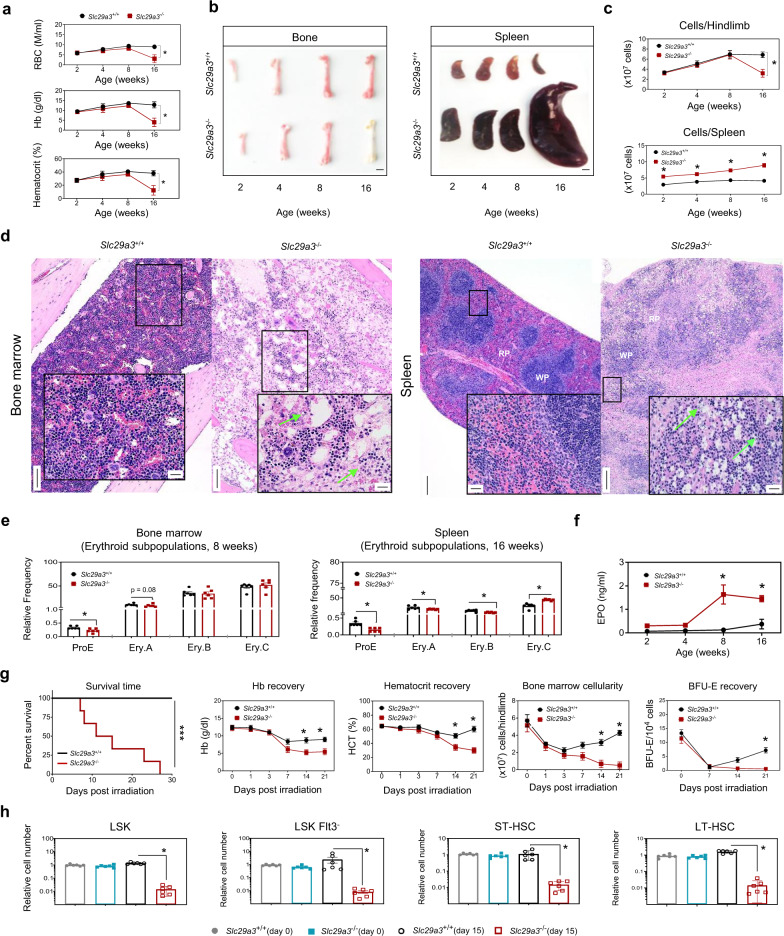
Reduction in HSPCs leads to progressively severe anemia in *Slc29a3*^*−/−*^ mice. **a** Quantification of blood parameters (red blood cell count (RBC), hemoglobin (Hb), and hematocrit) from 2 to 16- week-old *Slc29a3*^+/+^ (black) and *Slc29a3*^−/−^ (red) mice. Data represent mean ± SEM (*n* = 6 mice/group, *p < 0.05 by two-way ANOVA and Tukey’s post hoc test). **b** Representative images of femurs and spleens excised from 2 to 16-week-old *Slc29a3*^+/+^ and *Slc29a3*^−/−^ mice; Scale bar 2 mm. **c** Cellularity of excised femurs and spleens in *Slc29a3*^+/+^ (black) and *Slc29a3*^−/−^ (red) mice. Data represent mean ± SEM (*n* = 6 mice/group, *p < 0.05 by two-way ANOVA and Tukey’s post hoc test). **d** Representative H&E stained bone marrow (×10; Scale bar = 100 mm) and splenic sections (×4; Scale bar = 200 mm) comparing *Slc29a3*^+/+^ with *Slc29a3*^−/−^ mice at 16 weeks. One representative image from three independent experiments is shown for the bone marrow and spleen. Insets show higher magnification (×40; Scale bar = 20 mm) of boxed regions. Note, bone marrow is severely hypocellular with hematopoietic cell loss and erythroid progenitor cell death (green arrows) in *Slc29a3*^−/−^ mice. Also, severe atrophy of splenic WP with diffuse expansion of the RP with signs of hematopoiesis and erythroid cell death (green arrows). WP white pulp, RP red pulp. **e** Flow cytometry quantification of erythroid cell composition of bone marrow (8 weeks) and spleen (16 weeks) in *Slc29a3*^+/+^ (*black*) and *Slc29a3*^−/−^ (*red*) mice. Data represent mean ± SEM (*n* = 6 mice/group, *p < 0.05 by two-tailed *t*-test). **f** Plasma erythropoietin EPO levels in *Slc29a3*^+/+^ (black) and *Slc29a3*^−/−^ (red) mice from 2 to 16-week-old. Data represent mean ± SEM (*n* = 4 mice/group, *p < 0.05 by two-tailed *t*-test). **g** Survival time, Hb%, HCT%, bone marrow cellularity, and BFU-E recovery after 6.5 Gy total body irradiation (TBI) of *Slc29a3*^−/−^ (red) mice at 8 weeks old. Data represent mean ± SEM (*n* = 6 mice/group, *p < 0.05 by two-tailed *t*-test). Survival curves were plotted with the Kaplan–Meier method (*n* = 6/group, ***P < 0.001; Mantel-Cox test). **h** LSK, LSK Flt3^-^, ST-HSCs, and LT-HSCs were examined for relative cell number in 8-week-old *Slc29a3*^+/+^ mice pre (day 0) (gray) and post (day 15) (black) irradiation, and *Slc29a3*^−/−^ mice pre (day 0) (blue) and post (day 15) (red) irradiation. Data represent mean ± SEM (*n* = 6 mice/group, *p < 0.05 by ANOVA and Tukey’s post hoc test).

Since *Slc29a3*^−/−^ homozygous mice are anemic^13,14^, we studied the course of anemia and pathologies in *Slc29a3*^−/−^ mouse erythropoiesis. Examination of the blood parameters of *Slc29a3*^−/−^ mice^15^ identified that *Slc29a3*^−/−^ mouse pups had normal RBC counts, hemoglobin (Hb), and hematocrit levels until 8 weeks of age (Fig. 1a)^14^. Thereafter, mice developed progressive anemia that was severe at 16 weeks of age. Mice concurrently developed progressively hypoplastic bone marrow that was also evident at 16 weeks (Fig. 1b, c), and ultimately, developed severe aplastic bone marrow (Fig. 1d).

To determine if defects in the erythroid lineage contributed to the observed anemia in *Slc29a3*^−/−^ mice, we examined erythroid subpopulations within the bone marrow. We observed fewer proerythroblasts (proE) and basophilic erythroblasts (EryA) in *Slc29a3*^−/−^ mouse bone marrow before we could quantifiably determine the onset of anemia at 8 weeks (Fig. 1e). Erythroid subpopulations were gated to isolate cells at different stages of maturity; the relative number in each subpopulation demonstrated a shift toward more mature normoblasts (EryB and EryC; Fig. 1e). The decrease in proE in the bone marrow paralleled with an increase in circulating levels of erythropoietin (EPO), indicating the early onset of a systemic response to compensate for proE loss in *Slc29a3*^−/−^ mice (Fig. 1f). Within the first 2 weeks of life, *Slc29a3*^−/−^ mice developed splenomegaly with hypercellularity (Fig. 1b, c), which suggests early extramedullary compensation of anemia. Since splenic erythropoiesis is associated with stress erythropoiesis in mice after 8 weeks, we examined erythroid subpopulations of spleens of 16-week-old *Slc29a3*^−/−^ mice. The results showed a marked depletion of proE and EryA/B in the spleen of 16-week-old *Slc29a3*^−/−^ mice (Fig. 1e), despite significant counter-regulation by EPO release (Fig. 1f). In addition, we observed multifocal erythroid cell death in red pulp regions in spleens of 16-week-old *Slc29a3*^−/−^ mice with increased cleaved caspase-3 immunoreactivity in the spleen (Fig. S1). These results indicate that *Slc29a3*^−/−^ mice have defective generation of proE cells in the bone marrow and spleen.

When tested for primitive erythroid progenitors, we observed a decrease in both burst-forming unit-erythroid (BFU-E) and colony-forming unit-erythroid (CFU-E) colony formation from *Slc29a3*^−/−^ bone marrow-derived HSPCs at 12 weeks of age (Fig. S2). There was a trend of age-dependent decrease in BFU-E formation from HSPCs derived from the bone marrow, which was significant at 12 weeks of age. There was no difference in BFU-E formation from HSPCs derived from spleens of *Slc29a3*^−/−^ mice below 8 weeks, but a marginal increase in BFU-E formation was observed at 12 weeks. To evaluate if the differentiation defect observed in *Slc29a3*^−/−^ bone marrow HSPCs is erythroid-specific, we quantified the number and frequency of multipotent progenitors (MPPs) and more committed progenitors, including common myeloid progenitors (CMPs), megakaryocyte/erythrocyte progenitors (MEPs), and granulocyte/macrophage progenitors (GMPs; Fig. S3). We observed a significant decrease in MEPs and MPPs (MPP3) from *Slc29a3*^−/−^ bone marrow cells with a marginal increase in GMPs. These results identify the defective generation of erythroid-specific progenitors in *Slc29a3*^−/−^ mice.

The expansion of erythropoietic lineage depends on the proliferation of HSPCs; thus, we suspected that the erythroid hypoplasia observed in *Slc29a3*^−/−^ bone marrow and spleen might arise because HSCs are exhausted^14^. To test this hypothesis, we utilized a repair model to assess HSC and HSPC function. Since HSCs and HSPCs are more sensitive to erythropoietic stress than multipotent progenitor cells (MPP) or immediate erythroid precursors^16^, we used a radiation-induced repair model to assess HSC survival and blood regeneration. Notably, all *Slc29a3*^−/−^ mice died after sublethal irradiation (6.5 Gy whole-body irradiation), while all the *Slc29a3*^*+/+*^ mice survived up to 4 weeks post-exposure (Fig. 1g). *Slc29a3*^−/−^ mice almost completely lost the hematopoietic regenerative response during the recovery phase after radiation, as demonstrated by severely reduced Hb and hematocrit levels (Fig. 1g). Moreover, the failure of *Slc29a3*^−/−^ mice to recover bone marrow cellularity and primitive erythroid progenitors (burst-forming units (BFU-E); Fig. 1g) further supports this relationship between bone marrow erythroid hypoplasia and poor blood regenerative capacity. Consistently, there was a significant reduction in the relative numbers of HSPCs (Lin^−^IL7Ra^−^Sca1^+^c-kit^+^ (LSK), LSK Flt3^−^, short term (ST)-HSCs (LSK Flt3^−^ CD34^+^), and long term (LT)-HSCs (LSK Flt3^−^ CD34^−^)) on day 15 post-irradiation (Figs. 1h and S4). Thus, the long-term decrease in HSPCs and defective erythroid line of differentiation may underlie the impairment in blood cell regeneration and contribute to anemia in *Slc29a3*^−/−^ mice.

### Anemia in *Slc29a3*^−/−^ mice is associated with elevated basal ER stress and apoptosis in HSPCs

**Fig. 2:**
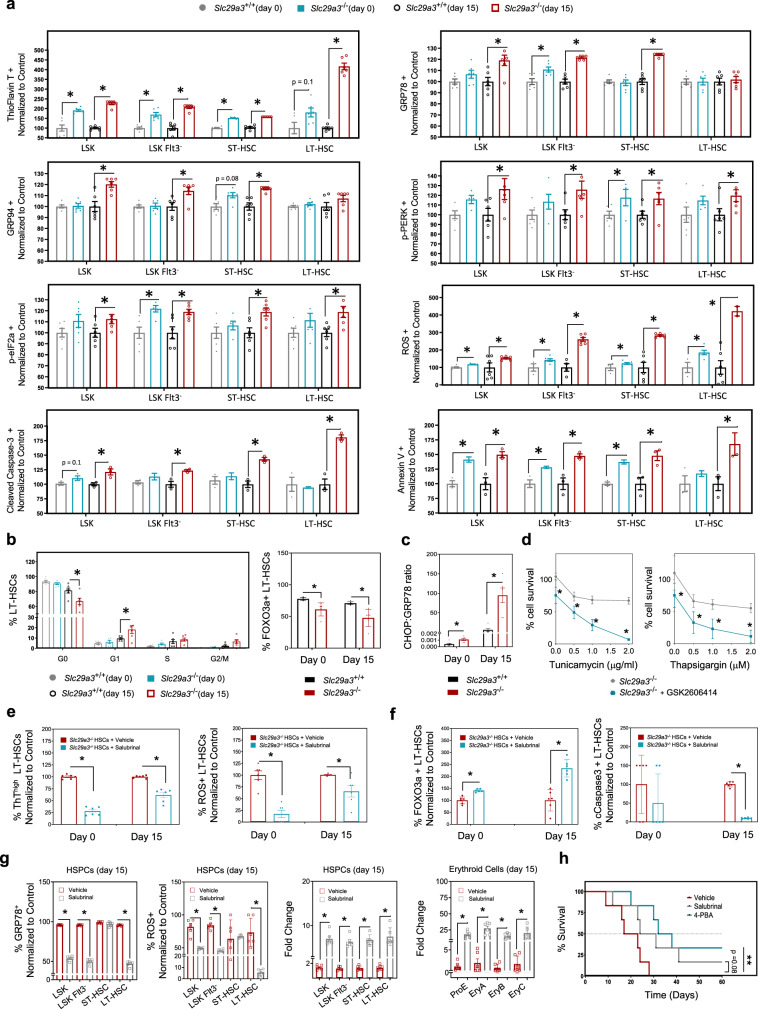
Anemia in *Slc29a3*^−/−^ mice is associated with elevated basal ER stress and apoptosis in HSPCs. **a** LSK, LSK Flt3^-^, ST-HSCs, and LT-HSCs were examined for the aggresomal marker Thioflavin T (TFT), ER stress markers (GRP78, GRP94, p-PERK, and p-eI2Fα), reactive oxygen species (ROS), and apoptotic markers (annexin V, and cleaved caspase-3) in 12-week-old *Slc29a3*^+/+^ mice pre (day 0) (gray) and post (day 15) (black) irradiation, and *Slc29a3*^−/−^ mice pre (day 0) (blue) and post (day 15) (red) irradiation. Data represent mean ± SEM normalized to *Slc29a3*^+/+^ pre (day 0) and post (day 15) irradiation (*n* = 6 mice/group, except cleaved caspase 3 and annexin V *n* = 3 mice/group, *p < 0.05 by ANOVA and Tukey’s post hoc test). **b** The proportion of LT-HSCs in quiescence (G_0_) or expressing FOXO3a in 8-week-old *Slc29a3*^+/+^ mice pre (day 0) (gray) and post (day 15) (black) irradiation, and *Slc29a3*^−/−^ mice pre (day 0) (blue) and post (day 15) (red) irradiation. Data represent mean ± SEM (*n* = 6 mice/group, *p < 0.05 by two-tailed *t*-test). **c** CHOP/GRP78 ratio in LT-HSCs in 8-week-old *Slc29a3*^+/+^ (black) and *Slc29a3*^−/−^ (red) mice pre (day 0) and post (day 15) irradiation. Data represent mean ± SEM (*n* = 6 mice/group, *p < 0.05 by two-tailed *t*-test). **d** Treatment with the PERK inhibitor (GSK2606414, 0.5 mM) (blue) or without (gray) alters cell death in *Slc29a3*^−/−^ HSPCs in the presence of tunicamycin (0–2 mg/ml) or thapsigargin (0–2 mM). Data represent mean ± SEM (*n* = 6 mice/group, *p < 0.05 by two-tailed *t*-test). **e** A proportion of LT-HSCs exhibiting high Thioflavin T fluorescence (ThT^high^) and ROS positivity (ROS^+^) when cultured in the presence of DMSO (vehicle; red) or salubrinal (10 mM; blue). Data represent mean ± SEM (*n* = 6, *p < 0.05 by two-tailed *t*-test). **f** Proportion of LT-HSCs positive for FOXO3a or cCasp3 when cultured in the presence of DMSO (vehicle; red) or salubrinal (10 mM; blue). Data represent mean ± SEM (*n* = 6, *p < 0.05 by two-tailed *t*-test). **g** Relative number of HSPCs and their subsets positive for GRP78 and ROS in *Slc29a3*^+/+^ and *Slc29a3*^−/−^ bone marrow after 6.5 Gy TBI followed by daily treatment vehicle (DMSO; red) or salubrinal (gray; 1 mg/kg, s.c.) and fold change of HSPCs and erythroid cells after 6.5 Gy TBI followed by daily treatment vehicle (DMSO) (red) or salubrinal (gray) (1 mg/kg, s.c.). Data represent mean ± SEM (*n* = 6, *p < 0.05 by two-tailed *t*-test). **h** Survival curves for 60 days were plotted with the Kaplan–Meier method (*n* = 6/group, ***p < 0.001; Mantel-Cox test). Vehicle (DMSO; red); Sal salubrinal (1 mg/kg, s.c; gray), 4-PBA 4-phenylbutyrate (10 mg/kg, s.c.; blue).

A high ER stress response is generally prevalent in lysosomal storage disorders resembling ENT3 spectrum disorders^17,18^. Further, the increase in erythroid cell death in the bone marrow and splenic red pulp regions in *Slc29a3*^−/−^ mice (Figs. 1d and S1) and our earlier finding that ex vivo cultured *Slc29a3*^−/−^ HSCs are hyperproliferative^14^ suggest that a high protein folding demand and ER stress response might cause damage that results in the decrease of *Slc29a3*^−/−^ HSPCs. We confirmed that mouse HSPCs express *Slc29a3* transcripts (Fig. S5a, b), and ENT3 protein is detected in the purified lysosome fraction isolated from mouse HSPCs (Fig. S5c), as reported earlier for other cell types^19,20^. To evaluate the mechanisms underlying the observed decrease in *Slc29a3*^−/−^ HSPCs, we analyzed the unfolded proteins and the unfolded protein response (UPR) in *Slc29a3*^−/−^ HSPC subsets using analytical cytometry (Figs. 2a, S4, S6, and S7). Consistent with our hypothesis, the levels of thioflavin T (ThT)-stained aggresomes (aggregation of misfolded proteins) and two major glucose-regulated ER stress proteins (GRP78 and GRP94) were highly elevated in *Slc29a3*^−/−^ HSPC subsets at 12 weeks of age (Fig. 2a). Additionally, the phosphorylated form of double-stranded protein kinase RNA-like endoplasmic reticulum kinase (pPERK), a major sensor that detects protein folding imbalances during ER stress, and the phosphorylated eukaryotic initiation factor 2 alpha (peIF2α), a substrate of PERK that terminates overall protein translation, were elevated in *Slc29a3*^−/−^ HSPC subsets at 12 weeks of age (Fig. 2a)^21–23^. Increases in GRPs (GRP78 and GRP94) and activation of the PERK signaling branch (pPERK, pEIF2α) was confirmed by immunoblotting analyses of *Slc29a3*^−/−^ ST-HSCs derived from 2, 5-, and 12-week mice (Fig. S8a). When analyzed for the involvement of other branches of UPR signaling^21–23^, we observed increased cleavage of activating transcription factor 6 (ATF6), increased phosphorylation of inositol-requiring enzyme 1 alpha (IRE1α), and increased splicing of X-box binding protein 1 (XBP1) in *Slc29a3*^−/−^ ST-HSCs at <8 weeks (2 and 5 weeks) of age but not (or less prominent) at 12 weeks (Fig. S8a, b and Supplementary Data 1). Together, these results indicate defective ER stress signaling associated with the PERK signaling branch may underlie the observed decrease in *Slc29a3*^−/−^ HSPCs. A similar UPR response is also observed in CMPs, MEPs, and ProEs derived from *Slc29a3*^−/−^ mice at 12 weeks of age (Fig. S9), which suggests the persistence of an ER stress defect along the erythroid line of differentiation.

Accumulation of aggresomes and ER stress markers occurred at a basal state (non-irradiated) but became exaggerated in day15 post-irradiation (Figs. 2a, S6, and S7); this suggests that pre-existing ER stress may cause *Slc29a3*^−/−^ mice to develop HSPC defects. Further, we observed *Slc29a3*^−/−^ HSPCs had increased reactive oxygen species (ROS), both at a basal state and day 15 post-irradiation (Fig. 2a); this finding also suggests a potential role of ER stress-mediated cell death in *Slc29a3*^−/−^ mice during hematopoietic stress. Consistently, the levels of annexin V and cleaved caspase-3 (cCasp3), markers of apoptosis, were increased in *Slc29a3*^−/−^ HSPC subsets, indicating that unresolved ER stress initiates apoptotic signals inducing cell death in *Slc29a3*^−/−^ HSPCs (Fig. 2a). Cell cycle analysis showed a loss of quiescence (reduced G0) in *Slc29a3*^−/−^ HSCs at day 15 post-irradiation with increased accumulation of cells at S phase (Figs. 2b and S4). *Slc29a3*^−/−^ LT-HSCs at day15 post-irradiation also showed reduced expression of FOXO3a quiescence marker (Fig. 2b), which, together with increased expression of annexin V and cCasp3, was suggestive of proliferative exhaustion of LT-HSCs in the absence of ENT3. Increased CHOP^+^/GRP78^+^ ratio in LT-HSCs derived from non-irradiated *Slc29a3*^−/−^ mice (Fig. 2c) further substantiated that the basal ER stress may induce cell death in *Slc29a3*^−/−^ LT-HSCs. Consistently, PERK inhibition in the presence of two chemical ER stressors (thapsigargin and tunicamycin) exacerbated ER stress response, tipping the balance from cell survival to cell death in *Slc29a3*^−/−^ LT-HSCs (Fig. 2d).

Remarkably, salubrinal, an ER stress inhibitor that reduces global protein synthesis by inhibiting dephosphorylation of eIF2α^24^, protected *Slc29a3*^−/−^ LT-HSCs from both ER stress and ROS (Figs. 2e and S10A, B). In addition, salubrinal treatment increased FOXO3a positivity and reduced cCasp3 positivity in *Slc29a3*^−/−^ LT-HSCs (Fig. 2f). Consistently, treatment of cultured *Slc29a3*^−/−^ HSPCs with salubrinal significantly inhibited the rapid loss of LT-HSC markers in progenies derived after passage (Fig. S10c)^14^. Moreover, salubrinal partially restored the differentiation defect in *Slc29a3*^−/−^ HSPCs by increasing the formation of BFU-E (Fig. S10d). To translate these findings, in vivo salubrinal treatment of irradiated *Slc29a3*^−/−^ mice was performed, which resulted in reduced ER stress and ROS in HSPCs, amplified erythroid and HSPC pool size, and increased survival time of the animals (Fig. 2g, h). Survival was further increased with in vivo treatment of a synthetic ER chemical chaperone, 4-phenyl butyric acid (PBA)^24–26^ (Fig. 2h). Collectively, these findings indicate that ER stress response is a putative mechanism contributing to HSPC dysfunction, which leads to impaired erythropoiesis in *Slc29a3*^−/−^ mice.

### Reintroduction of ENT3 alleviates ER stress response in transplanted *Slc29a3*^−/−^ HSPCs

**Fig. 3:**
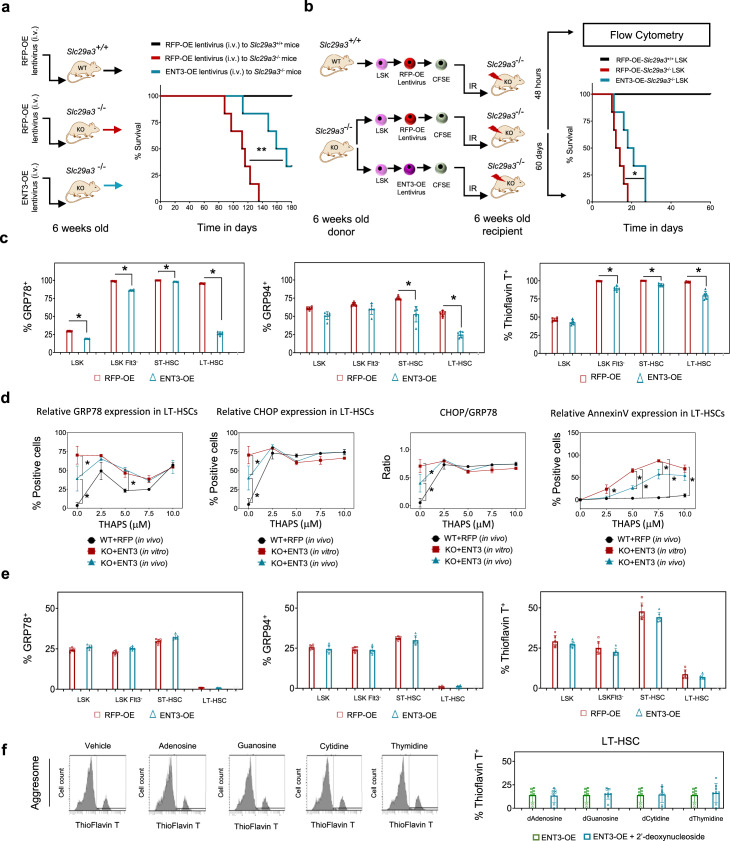
Ectopic expression of ENT3 in HSPCs ameliorates ER stress when transplanted into irradiated mice. **a** Experimental protocol (left) showing lentiviruses (10^8^ TU) overexpressing RFP (RFP-OE) or ENT3 (ENT3-OE) intravenously injected into the tail vein in *Slc29a3*^+/+^or *Slc29a3*^−/−^ mice at 6 weeks of age and monitored for survival. Kaplan–Meier analysis of survival for RFP-OE *Slc28a3*^*+/+*^(black), RFP-OE *Slc28a3*^−/−^(red), ENT3-OE *Slc28a3*^−/−^(blue) is presented (right) (*n* = 6/group; **p < 0.01; Mantel-Cox test). **b** Experimental protocol (left) showing single transplant of *Slc29a3*^−/−^ LSK cells after lentiviral overexpression of RFP (RFP-OE) or ENT3 (ENT3-OE) in 6.5 Gy irradiated *Slc29a3*^−/−^ mice. Prior to transplant, all cells are labeled with cell tracker CFSE fluorescent dye. Kaplan–Meier analysis of 60-day survival post-irradiation for RFP-OE *Slc28a3*^*+/+*^ *LSK* (black), RFP-OE *Slc28a3*^−/−^ *LSK* (red), and ENT3-OE *Slc28a3*^−/−^ *LSK* (blue) are presented (*n* = 6/group; *p < 0.001; Mantel-Cox test). **c** Quantification of positive cells for aggresomes and ER stress markers within each HSPC subpopulation overexpressing RFP (red) or ENT3 (blue) in vivo is presented. Data represent mean ± SEM (*n* = 6 mice/group, *p < 0.05 by two-tailed *t*-test). **d** Flow cytometry quantification of the proportion of LT-HSCs expressing CHOP, GRP78, CHOP/GRP78 ratio, and annexin V after exposure to thapsigargin (0–10 mM) for 3 h and recovery in fresh media for 24 h. CHOP/GRP78 ratio was normalized to relative GRP78 expression. Data represent mean ± SEM (*n* = 6 mice/group, *p < 0.05 by two-tailed *t*-test). WT + RFP (in vivo) (black), KO + ENT3 (in vitro) (red), and KO + ENT3 (in vivo) (blue). **e** Quantification of positive cells for aggresomes and ER stress markers within each HSPC subpopulation overexpressing RFP (red) ENT3 (blue) in vitro is presented. Data represent mean ± SEM. (*n* = 6 mice/group, statistical comparisons were insignificant by a two-tailed *t*-test). **f** Representative flow cytometry plots demonstrating the inability of 2′deoxynucleosides to reverse ER stress in cultured *Slc29a3*^−/−^ LT-HSCs even after introducing the *Slc29a3* gene (ENT3-OE) (left). Quantification results for ENT3-OE (green) and ENT3-OE + 2′-deoxynucleoside (blue) are shown (right). Data represent mean ± SEM. (*n* = 6 mice/group, statistical comparisons were insignificant by a two-tailed *t*-test).

The observed increase in ER stress markers in *Slc29a3*^−/−^ mice suggests that ER stress response machinery is intact, which allows for the activation of the UPR to engage and decrease the unfolded protein load to regain ER homeostasis. However, the persistence of high basal ER stress and induction of apoptosis after irradiation indicates that the ER stress recovery and UPR are compromised and fail to restore ER homeostasis in *Slc29a3*^−/−^ HSPCs. A single tail vein injection of mENT3 harboring lentiviruses (at 6 weeks of age) extended survival in *Slc29a3*^−/−^ mice (Fig. 3a). Furthermore, improved survival occurred when mENT3 transduced HSPCs were transplanted into irradiated *Slc29a3*^−/−^ mice at 6 weeks of age (Fig. 3b). We previously transplanted *Slc29a3*^*+/+*^ HSPCs into 10-week-old non-irradiated *Slc29a3*^−/−^ mice and *Slc29a3*^*−/−*^ mice transplanted with *Slc29a3*^*+/+*^ HSPCs observed 100% survival at 28 weeks of age with rescued bone marrow cellularity and anemia^14^. These results indicated that an ENT3-dependent, HSPC-specific effect is contributing to the increased survival of *Slc29a3*^−/−^ mice. However, *Slc29a3*^−/−^ HSPCs do not benefit from the *Slc29a3*^*+/+*^ niche and that *Slc29a3*^*+/+*^ HSPCs are not affected by the *Slc29a3*^−/−^ niche; these studies suggest that stem cell defects might occur in a cell-autonomous manner^14^. Therefore, niche-mediated regulation might be responsible for the increased survival of *Slc29a3*^−/−^ mice with transplanted HPSCs ectopically expressing ENT3. To test this hypothesis and to further probe into the mechanisms by which ENT3 reduces ER stress, we collected carboxyfluorescein succinimidyl ester (CFSE) prelabeled, bone marrow engrafted HSPCs within 24 h of intravenous infusion and evaluated the restoration of ER stress. To determine if transplanted HSPCs differed after homing the niche in vivo, we compared the amount of misfolded protein aggregates and ER stress in ENT3-RFP expressing *Slc29a3*^−/−^ HSPCs versus control (RFP expressing) *Slc29a3*^−/−^ HSPCs. Cells were gated on RFP positivity, and we discovered that engrafted ENT3 expressing *Slc29a3*^−/−^ HSPCs had a significant reduction of intracellular misfolded protein aggregates and ER stress markers (Fig. 3c). In addition, engrafted ENT3 expressing *Slc29a3*^−/−^ HSPCs exhibited reduced ER stress markers (GRP78, CHOP, and CHOP/GRP78 ratio) and resisted thapsigargin mediated apoptotic cell death (Fig. 3d) compared with RFP-alone expressing controls. These results suggest that ER stress reduction in vivo contributed to the rescue of HSPC functions.

To test if the factors provided by the bone marrow niche or circulation were responsible for the resolution of the ER stress response in ENT3 expressing *Slc29a3*^−/−^ HSPCs, we interrogated ER stress response in ENT3 expressing, cultured *Slc29a3*^−/−^ HSPCs. Particularly, we examined if ectopic ENT3 ameliorated ER stress by restoring protein folding in cultured *Slc29a3*^−/−^ HSPCs. Unexpectedly, the ThT-stained aggresome and the levels of GRP78 and GRP94 did not change significantly (*p* > 0.05) when ENT3 was ectopically expressed in cultured *Slc29a3*^−/−^ HSPCs (Fig. 3e); these results indicate that the presence of ENT3 alone was not sufficient enough to affect the ER stress response. Substrate concentration levels can affect the function of solute carrier transporters like ENT3. Thus, we suspected that the substrate concentration in the culture conditions (media) might limit ENT3 function. We investigated the *Slc29a3*^−/−^ HSPC ER stress response after replenishing the culture media with each of the four major 2′-deoxyribose nucleosides, which are the primary substrates of ENT3^27^. Despite nucleoside supplementation, the ER stress response persisted in ENT3 expressing *Slc29a3*^−/−^ LT-HSCs (Fig. 3f); this indicates that ER stress is independent of ENT3’s nucleoside transporting properties. A similar lack of recovery was evident with supplementation of a nucleobase (adenine) or a monoamine (serotonin), which are the secondary substrates of ENT3^27^. Overall, the discrepancy between the effect of ENT3 expression in vivo and in vitro remains unresolved. However, extraneous cellular factors ameliorated the ER stress in HSCs; this suggests that presently unknown ENT3 substrates in the bone marrow niche or circulation regulate ENT3 functions. Thus, ENT3 reintroduction and the bone marrow niche and/or circulatory factors were both required to restore the erythropoietic impairment of *Slc29a3*^−/−^ HSPCs.

### ENT3 is a low-affinity, acidic-pH dependent, and lysosomal transporter of BA chemical chaperones

**Fig. 4:**
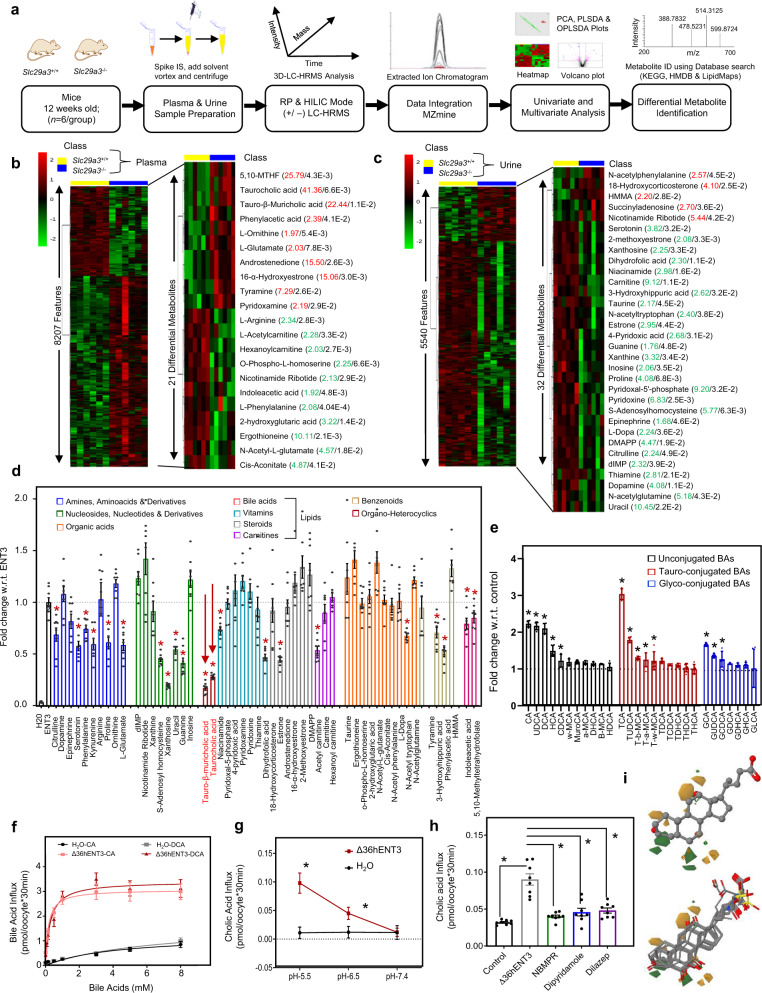
Untargeted metabolomics and transport analyses identify ENT3 as a facilitative transporter of bile acids. **a** Schematic workflow used to profile the plasma and urine metabolome of *Slc29a3*^+/+^ and *Slc29a3*^−/−^ mice. **b**, **c** Heat maps illustrating hierarchical clustering of differential features and metabolites detected across 6 *Slc29a3*^+/+^ (yellow) and *Slc29a3*^−/−^ (blue) mice plasma (left), and urine (right) samples by mass spectrometry-based metabolomics. MS signal intensities were clustered in two dimensions on the basis of Euclidean distance (row, metabolites; column, samples). Colors indicate the metabolite abundances (red, high; green, low). For identified metabolites, increased (red) or decreased (green) fold change in *Slc29a3*^−/−^ and corresponding *p*-value (black) indicated. **d** Inhibition of [^3^H]adenosine uptake at pH 5.5 in oocytes expressing del36hENT3 in the presence of differentially produced metabolites (100 µM). Colors indicate class of substrates, with red arrows highlighting bile acids. Data show the average ± SEM (*n* = 8 oocytes). *p < 0.05 (one-way analysis of variance compared with transport in uninhibited condition). **e** Fold increase in the transport of unconjugated BAs (black), tauro-conjugated BAs (red), and glyco-conjugated BAs (blue) (0.02 µM) in oocytes expressing del36hENT3 at pH 5.5 compared with water-injected oocytes. Transport was conducted in sodium-free transport buffer, and flux measurements were made using LC-MS/MS analyses. Data show the average ± SEM (*n* = 5 oocytes). *p < 0.05 (one-way analysis of variance compared with water-injected oocytes). **f** Concentration-dependent uptake of [^3^H]cholic acid (CA) (light red) and [^3^H]deoxycholic acid (DCA) (dark red) in oocytes expressing del36hENT3 at pH 5.5. Data show the average ± SEM (*n* = 12 oocytes). **g** pH-dependent transport of [^3^H]CA in del36hENT3 expressing oocytes (red), compared to H_2_O (black). Uptake of [^3^H]CA (0.02 μm) into oocytes was measured in transport solutions buffered to different pH. Data show the average ± SEM (*n* = 8 oocytes/group, *p < 0.05 by ANOVA and Tukey’s post hoc test). **h** [^3^H]CA uptake into Δ36hENT3 expressing was inhibited by NBMPR, dipyridamole, and dilazep at 10 µM. Data show the average ±SEM (*n* = 8 oocytes *p < 0.05 by ANOVA and Tukey’s post hoc test). **i** Contour representation of the key features from a CoMFA analysis of the 21 bile acids that showed any transport activity. Orange solids indicate regions where it is favorable to place steric functional groups, while green solids where it would be unfavorable. Orange wireframes indicate regions where it is favorable to place positive charges, while green wireframes where it would be unfavorable. The chemical structure of cholic acid (top) and alignment of 21 different BAs (bottom) are presented.

To screen for potential ENT3 substrates involved in ER stress reduction observed in vivo, we profiled plasma and urine from *Slc29a3*^−/−^ mice utilizing unbiased LC-HRMS metabolomics to identify metabolites specifically altered by a lack of ENT3 (Fig. 4a). ENT3 is an established transporter of polar compounds (nucleosides); accordingly, we adopted chromatographic (RP and HILIC) mode separations and mass spectrometric (positive and negative mode) ionization analyses to cover a wide range of endogenous metabolites from mid-polar to polar molecules. The analyses detected 4467 (Fig. 4b) and 5533 (Fig. 4c) metabolite features across the plasma and urine samples, respectively (Supplementary Data 2 and 3). By performing mass-based metabolite database searches (KEGG, HMDB, and LIPID MAPS) and comparing MS-MS fragmentation spectra with reference standards (Figs. S11 and S12), we identified distinctly altered metabolites including many amines and amino acid derivatives, lipid derivatives, benzenoids, and organic heterocyclics; at least 21 metabolites in plasma samples (Figs. 4b and S11) and at least 32 metabolites in urine samples (Figs. 4c and S12) of *Slc29a3*^−/−^ mice. To determine if any of the identified metabolites were ENT3 substrates, we performed an *X. laevis* oocyte-based transport inhibition assay to assess possible substrate competition with ^3^H-adenosine used as a probe ENT3 substrate^27^. Notably, we have reported earlier that the truncation of the 36 N-terminal amino acids, which harbor the intracellular targeting signal(s) in ENT3 (designated as del36ENT3), allows for the expression of ENT3 at the oocyte cell surface to characterize ENT3 lysosomal transport at pH 5.5 (measured as an influx in the oocyte assay; see Methods section)^2,27^. This analysis identified that 19 out of 51 metabolites including previously known ENT3 substrates serotonin (a monoamine; 42.24%; *p* < 0.0001) and xanthosine (a nucleoside; 80.95%; *p* < 0.0001) inhibited ENT3-mediated ^3^H-adenosine transport by 25–83% (Fig. 4d). To test if any of these 17 compounds (i.e., excluding serotonin and xanthosine) are genuinely ENT3 substrates, we conducted mass spectrometric analyses to directly measure the flux of each of these compounds into oocytes expressing hENT3. Remarkably, two BAs (tauromuricholic acid (TMCA) and taurocholic acid (TCA), which play a vital role as endogenous chemical chaperones to reduce ER stress, demonstrated transport mediated by ENT3 with 82.93% and 71.96% adenosine transport inhibition (*p* < 0.0001), respectively (Fig. 4d).

Direct analysis for ENT3 transport of 30 endogenous BAs using a more sensitive UPLC-MS/MS method was performed^28^, and found that 13 of the 30 BAs exhibited transport mediated by ENT3 (Fig. 4e). These results identify that a subset of endogenous BAs are substrates of ENT3. Among the BAs transported by ENT3, the highest amount of transport was demonstrated by TCA (3.04 fold; *p*, 0.005) followed by cholic acid (CA; 2.23 fold; *p*, 0.005), ursodeoxycholic acid (UDCA; 2.17 fold; *p*, 0.005), and deoxycholic acid (DCA; 2.10 fold; *p*, 0.005; Fig. 4e). We validated the observed ENT3-mediated transport of BAs with a radiometric oocyte assay using tritiated (^3^H)-CA and ^3^H-DCA as substrates (Fig. S13); we found a high correlation between both assays (*r*^2^ = 0.89). Kinetic analysis showed that BA flux in ENT3 injected oocytes (corrected for basal uptake in water-injected oocytes) was saturable and conformed to simple Michaelis–Menten kinetics (CA, *V*_max_: 3.07 ± 0.04 pmol/30 min/oocyte, *K*_m_: 167.5 ± 13.3 µM; DCA, *V*_max_: 3.4 ± 0.06 pmol/30 min/oocyte, *K*_m_: 269.6 ± 24.3 µM; Fig. 4f). In addition, ENT3-mediated transport of BA was dependent on an acidic pH (pH 5.5) consistent with its localization on the lysosomal membrane of HSPCs (Figs. 4g and S5c) and was inhibited by established ENT inhibitors (Fig. 4h). When ENT3-transported BAs were aligned with CA, quantitative structure-activity relationship (QSAR) models showed a high structure-activity relationship (SAR) with 98% accuracy as indicated by the regression coefficient (*r*^2^ = 0.98). When the derived QSAR models were visualized in 3D to study the SAR mechanism underlying BA transport; this transport activity was found dominated by steric effects, particularly by substitutions in positions 3, 7, and 12 of BAs with a minor role played by electrostatic interactions (Figs. 4i and S13). However, the cross-validation regression coefficient was minimal (*q*2 = 0.24), suggesting alternative transport mechanisms may be responsible for the ENT3-facilitated transport of BAs and adenosine.

### ENT3 facilitates ER accumulation of TBAs in HSPCs, and loss of ENT3 disrupts tissue disposition of BA

**Fig. 5:**
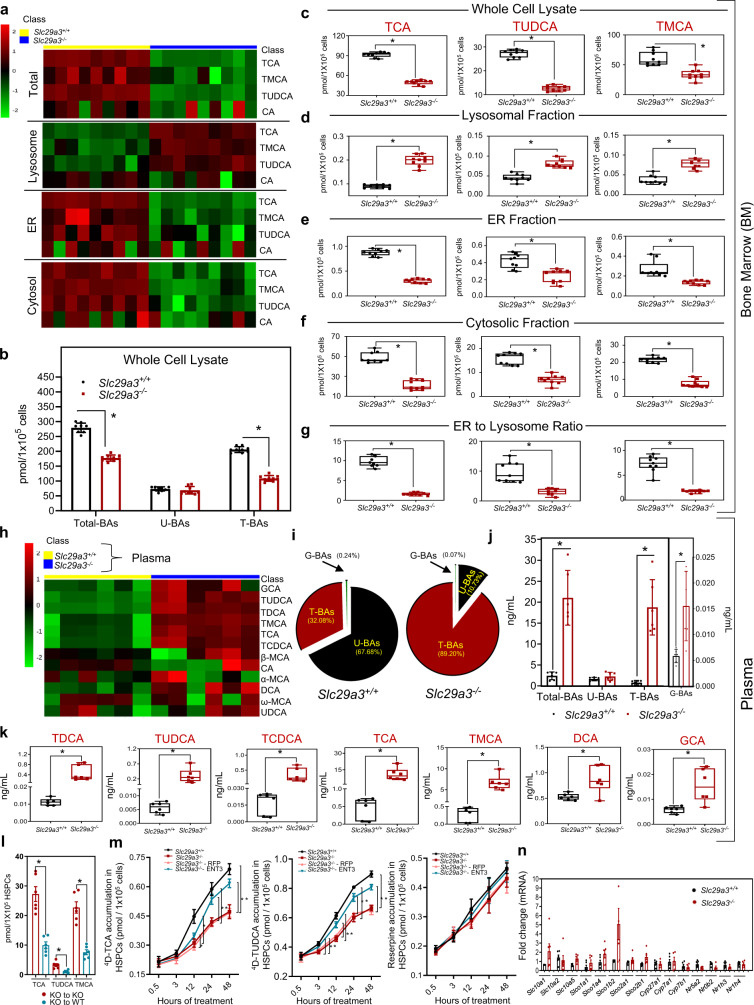
Targeted analysis of BA disposition in S*lc29a3*^+/+^ and S*lc29a3*^−/−^ mice. **a** Heat maps constructed for differentially identified BA as determined by targeted mass spectrometric analysis of total, cytosolic, ER, and lysosomal fractions from S*lc29a3*^+/+^ (yellow) and *Slc29a3*^−/−^ (blue) mice (12 weeks) bone marrow cells, respectively (*n* = 9 mice/group). MS signal intensities were clustered in two dimensions (row, metabolites; column, samples) on the basis of Euclidean distance. Colors indicate the metabolite abundances (red, high; green, low). **b** The concentrations of plasma total BAs, unconjugated BAs (U-BAs), taurine-conjugated BAs (T-BAs), and glycine-conjugated (G-BAs) in S*lc29a3*^+/+^ (black) and *Slc29a3*^−/−^ (red) mice bone marrow cells (whole cell lysate) as determined by LC-MS/MS analyses. Data represent mean ± SEM (*n* = 9 mice/group, *p < 0.05 by two-tailed *t*-test). **c–f** Box-whisker plot representation of alterations in BA levels in total, lysosomal, ER, and cytosolic fractions prepared from *Slc29a3*^+/+^ (black) and *Slc29a3*^−/−^ (red) mice bone marrow cells, respectively. Data represent mean ± SEM (*n* = 9 mice/group, *p < 0.05 by two-tailed *t*-test). **g** Box-whisker plot representation of estimated alterations in ER to lysosome ratio of BA levels in *Slc29a3*^+/+^ (black) and *Slc29a3*^−/−^ (red) mice bone marrow cells^.^ Data represent mean ± SEM (*n* = 9 mice/group, *p < 0.05 by two-tailed *t*-test). **h** Heat map representing hierarchical clustering of differentially identified BA as determined by targeted mass spectrometric analysis of *Slc29a3*^+/+^ (yellow) and *Slc29a3*^−/−^ (blue) (12 weeks) mouse plasma (*n* = 6 mice^/^group). MS signal intensities were clustered in two dimensions (row, metabolites; column, samples) on the basis of Euclidean distance. Colors indicate the metabolite abundances (red, high; green, low). **i**, **j** Pie chart and bar graph representation of changes in total, unconjugated (U-BA) and taurine- (T-BA) and glycine- (G-BA) conjugated BAs in S*lc29a3*^+/+^ and *Slc29a3*^−/−^ mice plasma samples. Data represent mean ± SEM (*n* = 6 mice/group, *p < 0.05 by two-tailed *t*-test). **k** Box-whisker plot representation of significantly altered BAs in *Slc29a3*^+/+^ (black) and *Slc29a3*^−/−^ (red) mice plasma as determined by targeted LC-MS/MS analyses. Data represent mean ± SEM (*n* = 6 mice/group, *p < 0.05 by two-tailed *t*-test). **l** Cellular content of TCA, TUDCA, and TMCA in the *Slc29a3*^−/−^ donor-derived (CFSE labeled) HSPCs harvested from irradiated S*lc29a3*^+/+^ (blue) or *Slc29a3*^−/−^ (red) recipient mice at 48 h after transplantation. Data represent mean ± SEM (*n* = 6 mice/group, *p < 0.05 by two-tailed *t*-test). **m** Uptake of ^4^D (deuterated)-TCA, ^4^D-TUDCA, and reserpine into *Slc29a3*^+/+^ (black), *Slc29a3*^−/−^ (red), RFP (light red), or ENT3 (blue) expressing *Slc29a3*^−/−^ HSPCs after 0.5, 3, 12, 24, and 48 h of incubation with the respective ^4^D-BAs. For L and M, samples were pooled from 5 to 6 mice/group to reach 1 × 10^5^ HSPCs to ensure that analytes were above the lower limit of quantitation in our LC-MS/MS assay. Data represent mean ± SEM (*n* = 5pooled mouse samples/group, *p < 0.05 by two-tailed *t*-test). **n** qPCR analysis of mRNA levels in LT-HSCs for transporters, metabolic enzymes, and nuclear receptors involved in BA homeostasis from *Slc29a3*^+/+^ (black) and *Slc29a3*^−/−^ (red) mice. Data represent mean ± SEM (*n* = 6 mice/group, *Slc29a3*^+/+^; *n* = 5 mice/group, *Slc29a3*^−/−^, statistical comparisons were insignificant by two-tailed *t*-test. All box plots in this figure represent the median (middle line), 25th, and 75th percentile (box), while the whiskers span from the minimum to the maximum value.

Taurine-conjugated BAs are a major class of BAs that endogenously attenuate ER stress^29^. Since ENT3 facilitated the transport of at least 6 out of 10 taurine-conjugated BAs, we tested whether the loss of ENT3 reduced the intracellular availability of taurine-conjugated BAs in *Slc29a3*^−/−^ HSCs as a probable mechanism of increased basal ER stress. Since LT-HSC levels are low in mice, we pooled LT-HSCs from multiple mice (10–12 mice/group) within each group to obtain sufficient cells for our analysis. Pooling samples allowed us to achieve TCA concentration (no other TBAs) above the lower limit of detection in our LC-MS assay, with ~50% reduction in TCA levels detected in LT-HSCs derived from *Slc29a3*^−/−^ mice. However, to overcome the issues related to limits of detection and to get more robust estimates for various TBAs, we used whole bone marrow cells for subsequent analysis. We found a uniform reduction in TCA (45.8%; *p* < 0.05), tauroursodeoxycholic acid (TUDCA; 53.5%; *p* < 0.05), and TMCA (43.8%; *p* = ns) in total bone marrow cells collected from 9 independent *Slc29a3*^−/−^ mice (Fig. 5a–c). Overall, there was a shift in the percentage of total taurine-conjugated BAs, decreasing to nearly 63.6% of the level of *Slc29a3*^*+/+*^ (Fig. 5b).

Since ENT3 is localized in the lysosome^19,20^, we next conducted subcellular fractionation of *Slc29a3*^−/−^ whole bone marrow cells to identify potential alterations in the organelle disposition of BA within HSCs. Compared to *Slc29a3*^*+/+*^ whole bone marrow cells, we found that the levels of TCA (119.8%; *p* < 0.05), TUDCA (77.4%; *p* < 0.05), and TMCA (108.3%; *p* < 0.05) were increased in the lysosomal fraction of *Slc29a3*^−/−^ whole bone marrow cells (Fig. 5a, d). This finding is consistent with the loss of ENT3 transport function across lysosomal membranes, causing sequestration of substrates within lysosomes. Contrary to increases in lysosomal content of taurine conjugated BAs, the levels of TCA (56.3% and 64.9%, respectively; *p* < 0.05), TUDCA (65.9% and 42.7%, respectively; *p* < 0.05), and TMCA (55.7% and 48.3%, respectively; *p* < 0.05) were decreased in the ER and cytosolic fractions of *Slc29a3*^−/−^ bone marrow cells (Fig. 5a, e, f) with an estimated decrease of 78.7% (*p* < 0.05) in ER to lysosomal ratio of TCA in *Slc29a3*^−/−^ bone marrow cells (Fig. 5g). Moreover, to address the prevalence of ENT3 transport defect, we conducted a targeted mass spectrometric analysis of bile acids in *Slc29a3*^−/−^ liver tissue, as the liver tissue normally expresses high levels of ENT3^27,30^. Similar to that seen with whole bone marrow cells (Fig. 5a–c), a significant decrease in the concentration of total BA and TBA with a significant increase in the concentration of UBA were observed in the liver of *Slc29a3*^−/−^ mice (Fig. S14a–d). Additionally, the lysosomal content of all BAs increased in the liver of *Slc29a3*^−/−^ mice (Fig. S14e–h). These results suggest that ENT3 is critical for the proper disposition of TBA in multiple tissues with loss of ENT3, resulting in lysosomal accumulation of BA.

### Loss of intracellular ENT3 and not plasma BA levels or cell surface BA transport limits BA availability in *Slc29a3*^−/−^ HSPCs

We measured the BA levels in *Slc29a3*^−/−^ plasma to assess if circulating BAs impacted the concentrations of BAs in the bone marrow niche and, thereby, the concentration of BAs in whole bone marrow cells. In addition to increased TCA and TMCA levels identified by untargeted metabolomics (Fig. 4b, c), the majority of the TBAs were found to be highly elevated (19.6–53.5 folds; *p* < 0.05) in *Slc29a3*^−/−^ plasma (Fig. 5h–k). Furthermore, we observed significant increases in the glycine-conjugate BAs (GCA (2.7-fold); *p* < 0.05) and a minor, non-significant increase in unconjugated BAs (UBA (1.1–1.3-fold)) in *Slc29a3*^−/−^ plasma (Fig. 5h–k). Since TBAs are the major BAs in mice^28^, the shifts in the percentage of total TBAs from 32.08% to 89.2% and total UBAs from 67.68% to 10.73 % resulted in an ~8.73-fold increase in total BA molar concentration in *Slc29a3*^−/−^ plasma (Fig. 5j). Accordingly, transplanted *Slc29a3*^−/−^ HSPCs (pooled from 5 to 6 mice/group) into irradiated *Slc29a3*^*+/+*^mice showed a further reduction in BA accumulation likely due to lower plasma BA content in *Slc29a3*^*+/+*^ mouse plasma compared to *Slc29a3*^−/−^ mouse plasma (Fig. 5l). Together, these results suggest that the availability of BA in the plasma does not affect the accumulation of BA into bone marrow cells of *Slc29a3*^−/−^ mice; the loss of ENT3 is likely responsible for the impaired accumulation of BA into bone marrow cells.

The reduced accumulation of BAs in *Slc29a3*^−/−^ mouse bone marrow cells despite the presence of high plasma BAs suggests potential impairment either in the cell surface uptake of BA in circulation (or residing in the bone marrow niche) or in the utilization of BA within bone marrow cells. To test these possibilities, we treated *Slc29a3*^*+/+*^ and *Slc29a3*^−/−^ HSPCs with deuterated (^4^d)-TCA at a roughly physiological total BA concentration (10 µM)^28^ and analyzed alterations in total cellular accumulation of labeled BA in short intervals of time (1–30 min). The initial flux rates ^4^d-TCA were not significantly different between *Slc29a3*^*+/+*^ and *Slc29a3*^−/−^ HSPCs up to 30 min; this result suggested that cell surface transport of BA was not responsible for the reduced accumulation of BAs (Fig. 5m). Furthermore, the transcript expression of major cell surface BA uptake transporters, metabolic enzymes, and nuclear receptors implicated in BA homeostasis did not vary significantly in *Slc29a3*^−/−^ LT-HSCs except for an adaptive increase in some *Slco* family members (Fig. 5n). To determine if the cellular utilization of BAs is altered in *Slc29a3*^−/−^ HSPCs, we repeated the ^4^d-TCA uptake experiment in *Slc29a3*^*+/+*^ and *Slc29a3*^−/−^ HSPCs utilizing longer time intervals (Fig. 5m). A reduction in accumulation was observed approximately after 12 h of treatment in *Slc29a3*^−/−^ HSPCs; this effect was more prominent at the end of 24–48 hours observation period (Fig. 5m), which is consistent with the lack of cellular utilization or metabolism of BAs^31^. The observed differences in accumulation after an extended duration (i.e., >12 hours) were ameliorated by ectopic expression of lentiviral-transduced ENT3 (and not RFP); these results suggest that ENT3 is essential for utilization of BA in HSPCs (Fig. 5m). Similar results were obtained with TUDCA, a substrate for ENT3, which is implicated in ER stress response (Fig. 5m), but not for reserpine, which is not transported by ENT3; both *Slc29a3*^*+/+*^ and *Slc29a3*^−/−^ HSPCs treated with reserpine accumulated similar levels of BAs up to 48 h (Fig. 5m). Similar transport trends were obtained when ^3^H-CA transport was conducted in HEK-293 cells and HEK-293 overexpressing ENT3 (Fig. S15). Collectively, these data suggest that the BA production in the liver or the circulating plasma TBAs in *Slc29a3*^−/−^ mice was not limiting BA availability in HSPCs. Rather, the loss of BA transport by ENT3, which sequesters BAs in lysosomes, impairs their cellular availability, and/or utilization in bone marrow HSPCs.

### ENT3 confers tauroursodeoxycholic acid-dependent in vivo gain-of-function to *Slc29a3*^−/−^ HSPCs

**Fig. 6:**
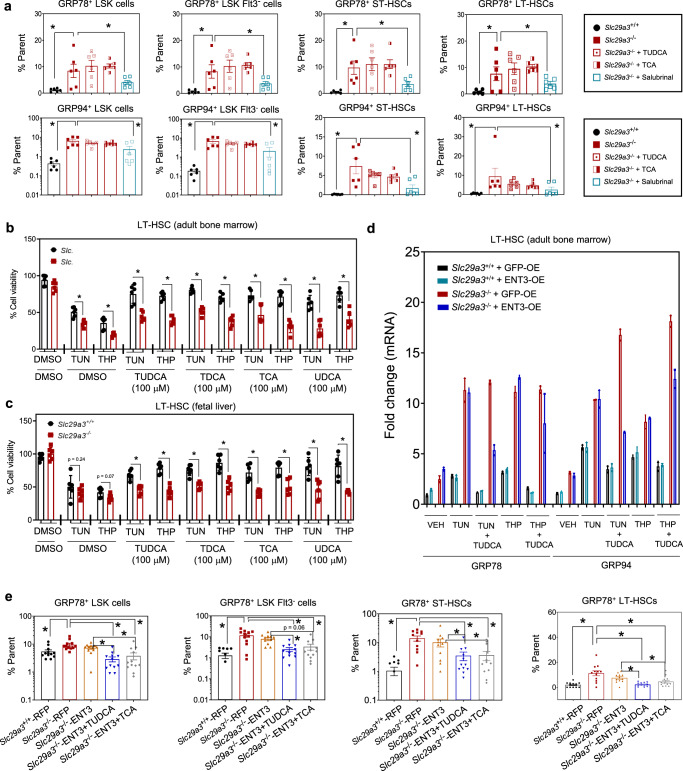
Ectopic expression of ENT3 confers taurine-conjugated bile acid-dependent amelioration of ER stress in cultured *Slc29a3*^−/−^ HSPCs. **a** Flow cytometry quantification of the proportion of *Slc29a3*^−/−^ HSPCs expressing ER stress markers GRP78 and GRP94 in the presence or absence of BAs. *Slc29a3*^*+/+*^(black), *Slc29a3*^−/−^(red), *Slc29a3*^−/−^ + TUDCA (red open box), *Slc29* *a3*^−/−^ + TCA (red half-filled box), and *Slc29a3*^−/−^ + Salubrinal (blue open box). Data represent mean ± SEM (*n* = 6 mice/group, *p < 0.05 by two-tailed *t*-test). **b** Taurine-conjugated BA (100 mM) promotes survival in *Slc29a3*^+/+^ (black) but not in *Slc29a3*^−/−^ (red) bone marrow derived L T-HSCs that are treated with tunicamycin (10 mg/ml) or thapsigargin (10 mM). Data represent mean ± SEM (*n* = 6 mice/group, *p < 0.05 by ANOVA and Tukey’s post hoc test). **c** Taurine-conjugated BA (100 mM) promotes survival in *Slc29a3*^+/+^ (black) but not in *Slc29a3*^−/−^ (red) fetal liver derived LT-HSCs that are treated with tunicamycin (10 mg/ml) or thapsigargin (10 mM). Data represent mean *±* SEM (*n* = 6 mice/group, *p < 0.05 by ANOVA and Tukey’s post hoc test). **d** qPCR analysis of mRNA levels for ER stress marker genes GRP78 and GRP94 after treating *Slc29a3*^+/+^ or *Slc29a3*^−/−^ LT-HSCs expressing GFP (GFP-OE) or ENT3 (ENT3-OE) with tunicamycin (2 mg/ml) or thapsigargin (2 mM) in the presence or absence of TUDCA. *Slc29a3*^*+/+*^GFP-OE (black), *Slc29a3*^*+/+*^*+* ENT3-OE (green), *Slc29a3*^−/− ^*+* GFP-OE (red), and *Slc29a3*^−/−^ + ENT3-OE (blue). Data represent mean ± SEM (*n* = 2 mice*/*group). **e** Flow cytometry-based quantification of proportion of HSPCs expressing ER stress markers GRP78 and GRP94 in *Slc29a3*^+/+^ or *Slc29a3*^−/−^ cells after reintroducing ENT3 and preconditioning with taurine-conjugated BA for 48 h. Data represent mean ± SEM (*n* = 12 mice/group, *p < 0.05 by two-tailed *t*-test).

The inhibitory effect of taurine-conjugated BAs on ER stress, which is reportedly vital for HSC function^29,32^, and the aforementioned finding that ENT3, an intracellular BA transporter, is indispensable for HSPC survival suggested that the observed recovery from anemia and lethality in vivo may depend on ENT3-BA interplay utilizing ENT3’s natural substrate in circulation. To study the regulation of ER stress by TBAs in *Slc29a3*^−/−^ HSPCs, we examined whether TCA or TUDCA-treated *Slc29a3*^−/−^ HSPCs effectively re-establish native protein folding by reversing pre-existing ER stress, as observed with salubrinal. We found that neither TCA nor TUDCA could inhibit expression of ER stress markers GRP78 and GRP94 in cultured *Slc29a3*^−/−^ HSPCs (Fig. 6a) or tunicamycin or thapsigargin induced cell death (Fig. 6b) in *Slc29a3*^−/−^ bone marrow LT-HSCs. Similar results were obtained with cultured *Slc29a3*^−/−^ fetal liver LT-HSCs (Fig. 6c). Overexpression of ENT3-GFP (without TUDCA) also produced no inhibition of GRP78 and GRP94 expression at transcript levels in *Slc29a3*^−/−^ LT-HSCs (Fig. 6d). In contrast, under the same in vitro conditions, both GRP78 mRNA and GRP94 mRNA in *Slc29a3*^−/−^ LT-HSCs (Fig. 6d), as well as the percentage of GRP78^+^ *Slc29a3*^−/−^ HSPCs (Fig. 6e), decreased after the concomitant ectopic expression of ENT3 and preconditioning with TUDCA or TCA for 48 h. These results provide further evidence for an integral role of ENT3 in the ER stress inhibitory function of BA in HSPCs.

**Fig. 7:**
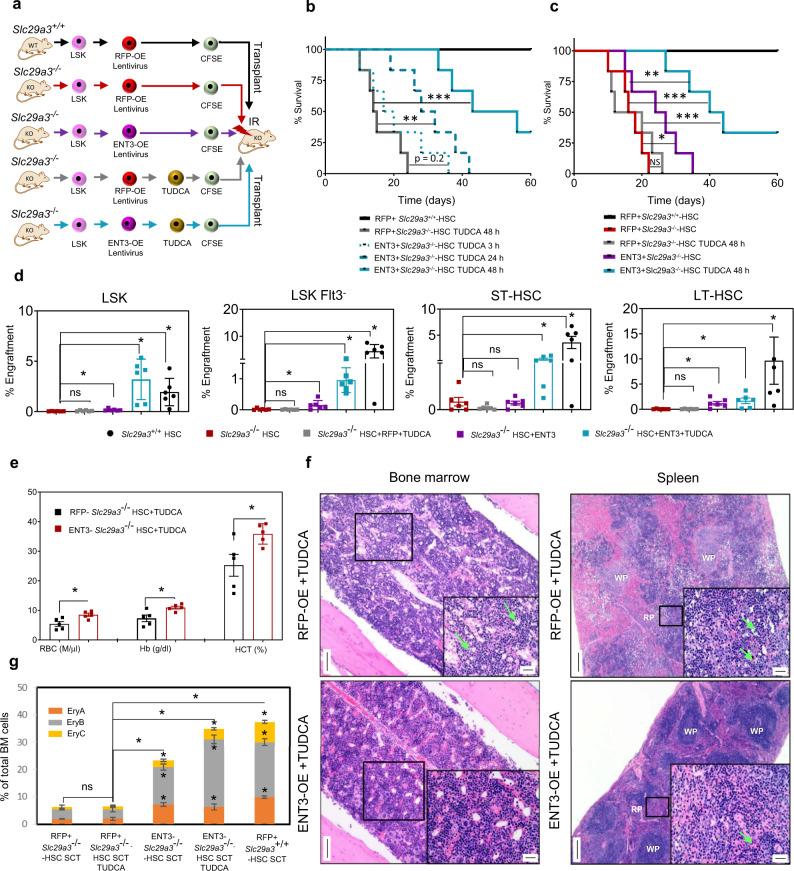
Ectopic expression of ENT3 in HSPCs confers TUDCA-dependent bone marrow homing and survival benefits in irradiated *Slc29a3*^−/−^ mice. **a** Experimental protocol showing LSK cells from *Slc29a3*^−/−^ mouse bone marrow transplanted into 6-week-old irradiated mice after lentiviral overexpression of RFP (RFP-OE) or ENT3 (ENT3-OE) with or without TUDCA preconditioning for 48 hours. **b** Kaplan–Meier analysis of 60-day survival after transplanting 6.5 Gy irradiated *Slc29a3*^−/−^ mice with RFP-OE or ENT3-OE LSK cells preconditioned with TUDCA for various periods or (**c**) in the presence or absence of TUDCA preconditioning of RFP-OE LSKs or ENT3-OE LSKs for 48 h (right) (*n* = 6/group; ***p < 0.001; Mantel-Cox test). RFP+*Slc29a3*^*+/+*^*-*HSC (black), RFP + *Slc29a3*^−/−^*-*HSC TUDCA 48 h (gray), RFP + *Slc29a3*^−/−^*-*HSC TUDCA 3 h (blue dots), RFP + *Slc29a3*^−/−^*-*HSC TUDCA 24 h (blue dashes), RFP + *Slc29a3*^−/−^*-*HSC TUDCA 48 h (blue), and RFP + *Slc29a3*^−/−^*-*HSC (red), and ENT3 + *Slc29a3*^−/−^(purple). **d** Percent HSPC bone marrow engraftment at 24 h after transplantation through the tail vein. Data represent mean ± SEM (*n* = 6 mice/group, *p < 0.05 by two-tailed *t*-test). *Slc29a3*^*+/+*^HSC (black), *Slc29a3*^−/−^ HSC (red), *Slc29a3*^−/−^HSC + RFP + TUDCA (gray*)*, *Slc29a3*^−/−^ HSC + ENT3 (*purple*), and *Slc29a3*^−/−^ HSC + ENT3 + TUDCA (blue). **e** Red cell indices of ENT3-OE + TUDCA conditioned HSPC transplanted *Slc29a3*^−/−^ mice (red) compared to RFP-OE + TUDCA conditioned HSPC transplanted *Slc29a3*^−/−^ mice (black). Data represent mean ± SEM (*n* = 6 mice/group, *p < 0.05 by two-tailed *t*-test). **f** Representative histological changes in bone marrow (H&E, magnification, ×10; Scale bar = 100 mm) and spleen (H&E, magnification, ×4; Scale; bar = 200 mm) of ENT3-OE + TUDCA conditioned HSPC transplanted *Slc29a3*^−/−^ mice compared with RFP-OE + TUDCA conditioned HSPC transplanted *Slc29a3*^−/−^ mice. One representative image from three independent experiments is shown for each condition in the bone marrow and spleen. Insets show higher magnification (×40; Scale bar = 20 mm) of boxed regions. The bone marrow is of normal cellularity with all lineages represented in the ENT3-OE + TUDCA conditioned mice compared to the hypocellularity with erythroid progenitor cell death (green arrows) seen in RFP-OE + TUDCA conditioned mice. The spleen of ENT3-OE + TUDCA conditioned mice reverts to normal architecture and hematopoietic state compared to atrophic white pulp (with diffuse expansion of the red pulp with hematopoiesis and erythroid cell death (green arrows)) noted in RFP-OE + TUDCA conditioned mice (5 out of 5 animals). WP white pulp, RP red pulp. **g** Flow cytometry quantification of erythroid cell composition of bone marrow (EryA (orange), EryB (gray), and EryC (yellow)) of ENT3-OE + TUDCA conditioned HSPC transplanted *Slc29a3*^−/−^ mice compared with RFP-OE + TUDCA conditioned HSPC transplanted *Slc29a3*^−/−^ mice. Data represent mean ± SEM (*n* = 6 mice/group, *p < 0.05 by two-tailed *t*-test). SCT stem cell transplant.

To determine if the functionality of taurine-conjugated BAs is impacted by ENT3 expression in HSPCs in vivo^29,32^, we assessed engraftment and hematopoietic regenerative potential of HSPCs in the bone marrow. We tracked functional HSPCs at 24 h after transplantation into irradiated *Slc29a3*^−/−^ mice. *Slc29a3*^*+/+*^ LSK cells, *Slc29a3*^−/−^ LSK cells, or *Slc29a3*^−/−^ LSK cells with RFP or ENT3 transgene expression, conditioned with or without TUDCA, were labeled with CFSE and injected into lethally irradiated syngeneic *Slc29a3*^−/−^ recipients (Fig. 7a). To assess the rapidity of protein folding as a function of HSPC^32–34^, we varied the preconditioning times from 3 to 48 h (Fig. 7b, c). Twenty-four hours later, the CFSE^+^ cells in the bone marrow were gated for each subpopulation and quantified by flow cytometry (Figs. 7d and S4). All *Slc29a3*^−/−^ HSPC subpopulations homed >5-fold less efficiently than the *Slc29a3*^*+/+*^ HSPCs derived from the respective donor LSKs (Fig. 7d). Homing of *Slc29a3*^−/−^ HSPCs after ENT3 expression was significantly improved (1.7-fold compared to RFP control) but remained less efficient than *Slc29a3*^*+/+*^ cells (7-fold compared to RFP control; Fig. 7d). There were no appreciable differences in RFP controls when HSPC subpopulations were analyzed in the presence or absence of TUDCA (Fig. 7d). Consistent with these effects on homing abilities, only in the presence of ENT3, preconditioning of *Slc29a3*^−/−^ HSPCs with 100 µM TUDCA resulted in enhanced survival of transplanted *Slc29a3*^−/−^ mice (Fig. 7b). With 3–48 h of TUDCA preconditioning, the percent survivors of ENT3 expressing group progressively increased from 8.3 to 68.3%, supporting that a time-dependent transport ability of ENT3 is associated with survival benefits (Fig. 7b). Similarly, the ENT3-dependency on TUDCA functions was evident by the lack of survival benefits from transplantation of TUDCA preconditioned (48 h), RFP expressing *Slc29a3*^−/−^ HSPCs (Fig. 7c). Consistent with the observation that ENT3 can derive benefit from the natural substrates (BAs) in vivo, expression of ENT3 in the absence of TUDCA preconditioning produced some survival benefits, albeit at a lower magnitude (50% animals survived 27 days) than that of expression of ENT3 in the presence of TUDCA preconditioning (50% animals survived 56 days) (Fig. 7c). Thus, in addition to cell autonomous effect^14^, these findings uncover a niche-mediated, nonautonomous effect responsible for the increased survival of *Slc29a3*^−/−^ mice with transplanted HPSCs ectopically expressing ENT3.

Finally, we compared the TUDCA-preconditioned (48 h), ENT3 expressing, *Slc29a3*^−/−^ HSPCs group with TUDCA-preconditioned (48 h), and the RFP expressing *Slc29a3*^−/−^ HSPCs group for erythroid lesions. In addition, we also evaluated ENT3 expressing without TUDCA preconditioning as a control and observed no difference. We observed increased hematopoietic regeneration as evidenced by improved peripheral RBC counts, percent Hb and hematocrit levels (Fig. 7e); this hematopoietic regeneration was further supported by a bone marrow and splenic erythroid regenerative response characterized by increased cellularity to bone marrow with all lineages represented, decreased extramedullary hematopoiesis of the spleen, and decreased erythroid precursor apoptosis in both the bone marrow and spleen (Fig. 7f). The splenic architecture returned to normal white pulp and red pulp in at least two out of five mice (Fig. 7f). Examination of bone marrow cell types revealed a near-complete rescue of erythroid precursors in TUDCA-preconditioned, ENT3 expressing, *Slc29a3*^−/−^ HSPCs transplanted animals, and only a partial regeneration in ENT3 expressing (no TUDCA preconditioning), *Slc29a3*^−/−^ HSPCs transplanted animals (supplemental Fig. 8g). Together, these data indicate that ENT3 facilitates TUDCA chemical chaperone function and that their interplay provides the necessary stimulus for erythropoietic regeneration.

## Discussion

In this report, we investigated ENT3-dependent mechanisms involved in erythropoiesis in bone marrow at steady-state and after recovery from sub-lethal radiation. First, we found that ENT3 plays an essential role in the maintenance of the erythroid lineage and the expansion of erythroid progenitor and precursor pools. Second, we identified that HSPCs lacking ENT3 have impaired hematopoietic homeostasis caused by abnormal protein folding and increased ER stress. Third, we uncovered that ENT3 is a low-affinity, acidic-pH dependent, lysosomal BA transporter, and that the loss of ENT3 results in increased lysosomal BA sequestration, which hinders cellular BA utilization compromising ER chemical chaperone activity. Finally, we demonstrated that the reintroduction of ENT3 restores the accumulation of BA in HSPCs, ameliorates ER stress, and improves erythropoiesis. These findings illustrate the mechanisms by which ENT3 regulates ER stress in erythroid progenitors, and how the loss of ENT3 results in impaired erythropoiesis and anemia noted in *Slc29a3*^−/−^ mice and several ENT3-mutated human genetic disorders.

Erythroid development is a dynamic, multistep process that relies upon erythroid lineage specification in HSPCs and the progression of multipotent erythroid progenitors to early erythroid precursors and mature erythrocytes. During erythroid development, HSCs orchestrate a demand-adapted switch between quiescent and active repopulation states^35^. In the present study, we identified elevated basal UPR signaling in *Slc29a3*^−/−^ HSPCs and along the erythroid line of differentiation, the UPR was predominantly mediated through the PERK signaling branch. Increases in ATF6 cleavage, IRE1α phosphorylation, and XBP1 splicing mainly observed in mice <8 weeks indicate that young mice experience general UPR signaling, and with age, this response becomes more restricted to the PERK signaling branch. Activation of the IRE1α signaling branch through IRE1α phosphorylation and XBP1 splicing provides cytoprotection against ER stress^21–23^. As a result, cytoprotective UPR signaling mediated through the IRE1α signaling branch is likely resisting anemia in young *Slc29a3*^−/−^ mice until 8 weeks of age. The age-dependent increase in ER stress present in *Slc29a3*^−/−^ HSPCs is related to increased cytotoxic UPR signaling through the PERK signaling branch^36^.

We also found that excess accumulation of misfolded proteins, ER stress, and ROS possibly contributes to loss of quiescence and defective repopulation abilities in *Slc29a3*^−/−^ HSCs. When we exposed *Slc29a3*^−/−^ mice to sub-lethal irradiation, *Slc29a3*^−/−^ HSCs that were deficient for ENT3 experienced increased apoptosis during recovery. Since apoptotic death at the basal state is minimal, it is likely that protective ER stress pathways are activated to offset constitutive UPR signaling^32,37,38^. However, sub-lethal doses of irradiation caused an increase in the demand for protein synthesis to repopulate HSCs. This increase in protein synthesis causes increased ER stress, which overwhelms UPR signaling and promotes apoptotic cell death signaling. The preservation of basal HSPC numbers and compensated anemia when mice were under 8 weeks of age further suggests that *Slc29a3*^−/−^ HSPCs are not defective in initiating replication or protein synthesis. Rather, *Slc29a3*^−/−^ HSPCs undergo apoptosis that is likely exacerbated during increasing animal age, developmental demands, or other replicative demands, including radiation stress. Other functional defects, including compromised bone marrow reconstitution potential and myeloid bias, were evident even under basal conditions in *Slc29a3*^−/−^ mice, and these results recapitulate the phenotypes observed in ER-stressed (GRP78+) HSCs^32,36^.

Previous studies have demonstrated that endogenous BAs can improve the general chaperone defense in HSCs and that endogenous BAs can fully compensate for molecular chaperones^29^. Elevating ER stress, then ameliorating the detrimental effects of ER stress through the administration of salubrinal or 4-PBA, established a cause and effect relationship between ER stress and ENT3 in *Slc29a3*^−/−^ HSPCs. However, the continued accumulation of unfolded proteins and ER stress proteins, despite the supplementation of TUDCA, suggests that endogenous BAs leave misfolded proteins largely unattended in the absence of ENT3. Several solute carrier BA transporters are implicated in the cell surface translocation of BA chemical chaperones^39^. However, the expression of canonical BA influx transporters and major BA homeostatic genes are largely unchanged in *Slc29a3*^−/−^ HSCs. Interestingly, the deletion of ENT3 caused increased accumulation of BA in plasma and poor retention in total, cytosolic, and ER fractions of bone marrow cells including LT-HSCs.

Unlike certain solutes (e.g., amino acids and cholesterol), the intracellular transport of BA remains poorly characterized^40^. The identification of ENT3 as a low-affinity, putative lysosomal BA transporter in this study suggested that ENT3 could promote subcellular trafficking of BA. In addition, we observed maximum ENT3-facilitated transport of BA at acidic pH values; this finding demonstrates that ENT3 transport optimally occurs in the acidic milieu of lysosomes. Furthermore, the observed transport maximum at pH 5.5 and the lack of transport of BA at pH 7.4 (cytosolic pH) suggests that ENT3 unidirectionally mobilizes BA from lysosome to cytosol and not from cytosol to lysosome increasing cytosolic concentrations of BAs. One of the key findings in the current study is that enhanced sequestration of BA in lysosomes of *Slc29a3*^−/−^ bone marrow cells occurs when cells are deficient of ENT3; concomitantly, BA levels in cytosol and ER decrease due to defects in BA lysosomal trafficking. Moreover, in addition to the erythroid lineage, we observed a significant decrease in the concentration of TBA in the liver of *Slc29a3*^−/−^ mice. These results suggest that lysosomal ENT3 is necessary for the subcellular transport of TBA in multiple tissues. It is likely that the erythroid lineage is particularly sensitive to the loss of lysosomal ENT3 because these cells, in the absence of TBA, are known to undergo elevated ER stress and apoptosis as HSPCs differentiate into erythroid cells. Our results support that the lack of ENT3-mediated transport of TBA fails to mobilize TBA from the lysosome and alleviate ER stress in *Slc29a3*^−/−^ HSPCs. Ultimately, this sequestration and decrease in BA levels contribute to the development of anemia resulting from functional ENT3 deficiency.

Intriguingly, the ablation of ENT3 not only decreased the cytosolic and ER levels of BA in tissues but also dramatically increased plasma accumulation of BA. The mechanism of how the deficiency of a lysosomal BA transporter alone, which handles only a low amount of BA, can bring drastic effects on the subcellular, cellular, and tissue disposition of BA is not completely clear, but it is likely that ENT3 facilitates continuous recycling of BA from lysosomes to the cytosol and that lysosomal sequestration of BA in the absence of ENT3 negatively regulates the cellular accumulation and/or utilization of BA in multiple body tissues sufficient to affect the plasma BA concentration. In this regard, the increased lysosomal retention of endogenous BA in various *Slc29a3*^−/−^ cell types, the delayed cellular accumulation of extraneous BA into *Slc29a3*^−/−^ cells, and the dysregulation of lysosomal signaling pathways in the absence of ENT3^14^ supports this proposition. Furthermore, the loss of ENT3 and tissue utilization of BA may increase the production of BA in the liver through a feedback mechanism, since increased levels of UBA was observed in *Slc29a3*^−/−^ livers. Increased plasma BA can have systemic cytotoxic effects and has been shown to contribute to adverse cellular effects including cancer development^41,42^. Interestingly, in addition to anemia, loss of ENT3 leads to the development of histiocytic sarcoma^19^, a cancer of hematopoietic origin cardinally observed in ENT3 null mice.

We observed that reintroduction of ENT3 increased the cellular accumulation of BA in a gradual, time-dependent manner. These results suggest that intracellular BA transport facilitates cellular BA utilization and/or metabolism and that occurrence of these processes draws more flux from the extracellular milieu. Our findings are supported by other studies that have demonstrated that lysosomal storage disorders may be caused by deficient lysosomal transport of solutes preventing cellular metabolism of substrates. For example, Niemann Pick disorder is caused by reduced cholesterol efflux from the lysosome into the cytosol and metabolism caused by mutations in the lysosomal transporters NPC1 and NPC2^43^. Another possible mechanism is that the impaired lysosomal proteolytic system in *Slc29a3*^−/−^ mice might have worsened the accumulation of ER stress and subsequent ROS production, causing a failure to sustain the number of HSC in *Slc29a3*^−/−^ mice^17^. Thus, these findings support that the lysosomal mobilization of BA and perhaps, lysosomal homeostasis per se are necessary elements for the ER stress-reducing function of BA, particularly in HSCs.

Deficiencies in ENT3-mutated lysosomal disorders sustain ER stress-mediated UPR pathways that are eventually detrimental to HSC self-renewal and erythroid differentiation. The failure of lysosome-sequestered BA chaperones to reduce ER stress and recovery by ectopically expressed ENT3 suggests that BA selectively depends on ENT3-mediated lysosomal transport to achieve optimal concentrations in the cytosol and the ER to maintain HSC homeostasis. Functionally, this ability is reflected by the alleviation of ER stress in ENT3-introduced, TUDCA preconditioned *Slc29a3*^−/−^HSCs. Altogether, our data support ENT3 as a critical regulator of chemical chaperone defense in hematopoietic stem and progenitor cells.

In summary, the current study uncovers the fundamental role of ENT3 in regulating hematopoietic homeostasis by conducting retrograde transport of lysosomal BA required for normal mouse erythropoietic development. Our findings present both pathophysiological and therapeutic implications for understanding the role of ENT3 in erythroid biology and treating anemia observed in several human genetic disorders occurring due to mutations in hENT3 or nucleoside analog-based drug therapy.

## Methods

### Chemicals, reagents, and antibodies

Bile acid standards viz. cholic acid (CA), ursodeoxycholic acid (UDCA), deoxycholic acid (DCA), hyocholic acid (HCA), chenodeoxycholic acid (CDCA), alpha-muricholic acid (α-MCA), beta-muricholic acid (β-MCA), omega-muricholic acid (ω-MCA), murocholic acid (Muro CA), dehydrocholic acid (DHCA), hyodeoxycholic acid (HDCA), taurocholic acid (TCA), TUDCA, tauro-alpha-muricholic acid (T-α-MCA), tauro-beta-muricholic acid (T-β-MCA), tauro-omega-muricholic acid (T-ω-MCA), taurodeoxycholic acid (TDCA), taurochenodeoxycholic acid (TCDCA), taurodehydrocholic acid (TDHCA), taurohyocholic acid (THCA), taurohyodeoxycholic acid (THDCA), glychocolic acid (GCA),glycoursodeoxycholic acid (GUDCA), glycochenodeoxycholic acid (GCDCA), glycodeoxycholic acid (GDCA), glycodehydrocholic acid (GDHCA), glycohyocholic acid (GHCA), and glycolithocholic acid (GLCA) were procured from Steraloids (Newport, RI, USA). Additionally, deuterated bile acid standards, i.e, taurocholic acid-d4 were obtained from Cayman Chemicals (Ann Arbor, MI, USA); TUDCA-d4 was purchased from Sigma-Aldrich (St. Louis, MO, USA). Internal standards viz. reserpine, 4-nitrophenol, ^4^d-cholic acid and ^4^d-glycocholic acid were procured from Sigma-Aldrich (St. Louis, MO). LC-MS grade methanol, acetonitrile, ammonium acetate, formic acid, and ammonium hydroxide were obtained from Sigma-Aldrich (St. Louis, MO, USA). Deionized distilled water was prepared in-house using a Milli-Q (Millipore) water purification system and was further filtered through a 0.22-µm filter prior to use. All other chemicals and reagents used in this study were of AR grade.

Recombinant mouse erythropoietin, SCF, and mouse erythropoietin quantikine ELISA kits were purchased from R&D (Minneapolis, MN, USA). Thioflavin T (ThT) was obtained from Sigma Aldrich (St. Louis, MO, USA)). The total ROS detection kit was obtained from Enzo Life Sciences (Farmingdale, NY, USA). Plasmids pLenti-CMV-RFP-2A-Puro-Blank control and pLenti-GIII-CMV-RFP-2A-Puro-*Slc29a3* vectors were purchased from Applied Biological Materials (Richmond, BC, Canada). Trans Lentiviral Packaging Mix was obtained from Dharmacon (Lafayette, CO, USA). CellTrace CFSE cell proliferation kit was purchased from Life Technologies (Carlsbad, CA, USA). StemSpan Serum-free medium for culture and expansion of HSCs was purchased from Stem Cell Technologies (Vancouver BC, Canada).

Antibody conjugates for Flt3 (PE-CF594-anti-CD135, clone A2F10.1; 1:50 dilution), IL7Ra (V450-anti-CD127, clone SB/199; 1:50 dilution), Sca1 (PE-Cy7-anti-Ly-6A/E, clone D7; 1:50 dilution), and CD34 (FITC-anti-CD34, clone RAM34; 1:50 dilution) were obtained from BD Biosciences (San Jose, CA), while c-kit (PE-anti-CD117, clone REA791; 1:50 dilution) was obtained from Miltenyi Biotech (Bergisch Galadbach, Germany). Alternative conjugates for CD34 (Alexa Fluor 700-anti-CD34, clone RAM34; 1:50 dilution), IL7Ra (PE-CF594-anti-CD127, clone SB/199; 1:50 dilution), and Flt3 (APC-anti-CD135, clone A2F10.1; 1:50 dilution) were also obtained from BD Biosciences (San Jose, CA, USA). The Lineage Cell Detection Cocktail-Biotin and the ani-Biotin secondary antibody (PerCP-Vio700-anti-Biotin, clone Bio3-18E7, 1:50 dilution) was obtained from Miltenyi Biotech (Bergisch Galadbach, Germany).

The unconjugated ER stress antibody for p-eI2Fα (anti-p-eIF2α, clone EPR11042; 1:20 dilution) was were obtained from Cell Signaling Technology (Danvers, MA, USA), the p-PERK (anti-p-PERK, clone Thr980; 1:20 dilution) was obtained from ThermoFisher Scientific (Waltham, MA, USA), and antibodies for GRP78 (anti-GR78/BIP, clone EPR4041; (2) 1:20 dilution) and GRP94 (anti-GRP94, clone EPR22847-50, 1:20 dilution) were obtained from Abcam (Cambridge, United Kingdom). The antibody for the apoptotic marker cleaved caspase-3 (anti-cleaved-caspase-3, clone 299518, 1:20 dilution) was obtained from R&D Systems (Minneapolis, MN) and the APC-Annexin V Kit from BioLegend (San Diego, CA, USA) was used to stain for the apoptotic marker Annexin V. The antibody recognizing the cell cycle marker Ki-67 (APC-anti-Ki-67, clone SolA15, 1:50 dilution) and the DNA dye Hoechst 33342 were obtained from ThermoFisher Scientific (Waltham, MA, USA).

The stem cell transplant flow cytometry was performed using APC-mouse lineage antibody cocktail (1:5 dilution), BUV395 anti-Sca1 (1:50 dilution), APC/Cy7 anti-Sca1 (1:50 dilution), PE-Cy7 anti-CD117 (1:50 dilution), FITC anti-CD34, PerCP/Cy5.5 anti-CD34 (1:50 dilution), PE anti-CD135 (Flt3) (1:50 dilution), and PerCP/Cy5.5 anti-CD71 (1:50 dilution) antibodies were purchased from BD Biosciences (San Jose, CA, USA). Biotin Anti-mouse TER119 (1:50 dilution) and APC streptavidin (1:5000) were purchased from BioLegend (San Diego, CA, USA).

### Breeding, genotyping, and maintenance of *Slc29a3*^−/−^ mice

The *Slc29a3*^+/−^ gene trap knockout line on a 129S5/SvEvBrd × C57BL6/J hybrid background (Mutant Mouse Resource and Research Center) was used to generate *Slc29a3*^+/+^, Slc29a3^+/−^, and *Slc29a3*^−/−^ littermates. Mice were backcrossed 10 times. In total, 3–12 mice per experiment were used in the study. Mice from both sexes were included. Mice were housed in a vivarium with controlled temperature (20–26 °C) and humidity (40–60%), maintaining a 12/12-h dark/ light cycle. Standard pellet diet and water were provided ad libitum. After the animals fasted for 12 h on the 12th week, simultaneous blood from the facial vein and urine samples were collected in heparin and microcentrifuge tubes, respectively.

### Isolation of hematopoietic subpopulations and identification of ER stress and apoptosis with flow cytometry

HSPCs were isolated and cultured following our previously published protocol^14^. Briefly, HSPCs were cultured in SFEM medium (STEMCELL Technologies, Vancouver, BC, Canada) containing 100 ng/ml SCF, 20 ng/ml TPO, and 20 ng/ml IL-3 and all experiments were conducted using fresh HSPCs within 2 weeks of isolation. Pooled HSPCs from multiple mice were used for experimental approaches that needed more cells as with transplantation, bone marrow engraftment analyses, and immunoblots.

To analyze subpopulations of cells in the spleen, mouse spleen cells were prepared by mechanical disruption of the spleen in phosphate buffer saline (PBS) and filtered through 40 µm pore size cell strainer (VWR, PA, USA). For bone marrow analyses, the marrow was flushed with PBS and passed through a 25-gauge needle and filtered through a 40-µm pore size cell strainer (VWR, PA, USA). Freshly harvested bone marrow cells were c-kit-enriched using c-kit-conjugated magnetic beads (Miltenyi, Bergisch Gladbach, Germany), and subsequently costained with antibodies against lineage markers (Lineage Cell Detection Cocktail, Miltenyi), Flt3 (PE-CF594-anti-CD135), IL7Ra (V450-anti-CD127), c-kit (PE -anti-CD117), Sca1 (PE-Cy7-anti-Ly-6A/E), CD34 (FITC-anti-CD34), FcgRII/III (BV605-anti- FcgRII/III), and CD150 (BV421-anti-CD150), and CD48 (APC-Cy7-anti-CD48). After cell surface staining, cells were fixed and permeabilized using BD Cytofix/Cytoperm (BD Biosciences, Bedford, MA) and stained for the intracellular ER stress markers (GRP78, GRP94, p-eIF2α, and p-PERK) or apoptotic markers (annexin V and cleaved caspase-3). The APC fluorochrome was conjugated to unconjugated ER stress or apoptotic markers using the Zenon Mouse IgG Labeling Kit (Life Technologies, Carlsbad, CA) and used for downstream experiments. An alternative HSPC flow cytometry panel was used in conjunction with the aggresome marker ThioFlavin T (TFT). In these experiments, c-kit-enriched cells were subsequently costained with antibodies against lineage markers (Lineage Cell Detection Cocktail, Miltenyi), Flt3 (APC-anti-CD135), IL7Ra (PE-CF594-anti-CD127), c-kit (PE -anti-CD117), Sca1 (PE-Cy7-anti-Ly-6A/E), and CD34 (Alexa Fluor 700-anti-CD34). After fixation and permeabilization, cells were stained with TFT, which naturally emits fluorescence between 445 nm and 482 nm and were visualized under Indo-1 violet. The LIVE/DEAD Fixable Near-IR Dead Cell Stain Kit (ThermoFisher Scientific, Waltham, MA) was used to exclude dead cells. Cell staining was performed on ice and analyzed using a BD LSRFortessa equipped with five lasers (355, 405, 488, 561, and 633 nm). Color compensation was performed using BD FACSDIVA 8.0.1 Software (BD Biosciences, Bedford, MA), and FACS data analysis was performed using FlowJo 10.4.2 (Tree Star, Ashland, OR).

LSK, LSK Flt3− short-term HSC (ST-HSC), and long-term HSC (LT-HSC) cell types were quantified following gating schemes described in Supplemental Fig. 7^14^. Within each HSPCs population, we subsequently measured ER stress markers, apoptotic markers, and the aggresomal marker TFT (Fig. S7).

The erythroid cells were initially isolated from the bone marrow based on positivity for CD71 and TER119 and further resolved based on a forward scatter (FSC) or cell size into CD71^high^Ter119^med^ proerythroblasts (ProE), CD71^high^Ter119^high^FSC^high^ basophilic (Ery.A), CD71^high^Ter119^high^FSC^low^ late basophilic or polychromatic (Ery.B), and CD71^low^Ter119^high^FSC^low^ orthochromatic (Ery.C) erythroblasts as described in Supplemental Fig. 7^14^.

C/EBP homologous protein (*CHOP*) and cleaved caspase*-3* were assessed by Alexa Fluor 647 anti-GADD153/CHOP (Novus Biologicals, CO, USA) and FITC anti-active caspase3 (BD Biosciences, San Jose, CA, USA). The proportion of quiescent (G_0_) cell populations and cell cycle distribution (G_1_, S or G_2_/M) was quantified by the simultaneous analysis of proliferative marker (Ki-67; ThermoFisher Scientific, Waltham, MA, USA) and cellular DNA content (staining of Hoechst 33342 (ThermoFisher Scientific, Waltham, MA, USA)).

### Transplantation and engraftment assays

*Slc29a3*^*+/+*^ and *Slc29a3*^−/−^ mice were subjected to total body irradiation (TBI) using 6.5 Gy in the Small Animal Radiation Research Platform (SARRP) in collaboration with the OSU Small Animal Imaging Core. The ionizing radiation dose of 6.5 Gy was selected to deliver a sublethal dose in *Slc29a3*^*+/+*^ mice. After irradiation, the recipient mice were intravenously injected with 1 × 10^4^ bone marrow-derived *Slc29a3*^+/+^ and *Slc29a3*^−/−^ HSPCs that underwent a diverse range of treatments including ENT3 or RFP overexpression, bile acid (TUDCA) pre-conditioning and CFSE labeling in a sequential manner prior to transplant. After 48 h, TCA, TUDCA, and TMCA in the *Slc29a3*^−/−^ donor-derived (CFSE labeled) HSPCs were quantified from pooled samples (5–6 mice/group). The percentage engraftment of donor HSPC subpopulations after gaining access to the recipient environment was assessed by observing CFSE positivity. The CFSE positive cells were sorted and subsequently analyzed for positivity to thioflavin T fluorescence (TFT; aggresomes) and ER stress protein expression by fluorochrome labeled antibodies (PerCP/Cy5.5 anti-GRP78 and PerCP/Cy5.5 anti-GRP94).

### LC-FTMS metabolomics profiling

Separation of metabolites in the RP mode was achieved using a Scherzo SMC_18_ column (150 mm × 4.6 mm, 3 µm particle size) from Imtakt (Portland, OR). The mobile phase for RP positive as well as negative analysis was Solvent A, Water (10 mM Ammonium Acetate, 0.1% FA), and Solvent B, Acetonitrile (0.1% FA). The flow rate was set to 0.5 mL/min and the optimized gradient conditions were 0 min- 5%B; 2 min- 5%B; 18 min- 20%B; 23 min- 20%B; 24 min- 5%B; and 30 min- 5%B. The SMC_18_ column was maintained at 40 °C throughout the experiment.

For the separation of metabolites in the HILIC mode, a Luna NH_2_ column (100 mm × 2 mm, 3 µm particle size) from Phenomenex (Torrance, CA) was used. The mobile phase for HILIC positive as well as negative analysis was Solvent A, Water (20 mM Ammonium Acetate, 0.06 M ammonium hydroxide) and Solvent B and Acetonitrile. The optimized gradient conditions were: 0 min- 95%B; 2 min- 95%B; 18 min- 10%B; 23 min- 10%B; 24 min- 95%B; 30 min- 95%B, and the flow rate was set at 0.3 mL/min. The NH_2_ column was maintained at 30 °C throughout the experiment.

Before the beginning of each experiment, the mass spectrometer was calibrated as per the manufacturer’s specifications. Data were acquired in the centroid mode using full scan MS method. The mass range was scanned from *m*/*z* 50–1200 at 0.1 scan/sec. Data were collected separately in positive and negative electrospray ionization mode. The general mass spectrometer operating conditions for RP and HILIC mode were Spray voltage: +4.5 kV for positive mode and −3.5 kV for negative mode; HESI temperature: 350 °C; Vaporizer temperature: 450 °C, Sheath gas: 35 arbitrary units; Auxiliary gas: 10 arbitrary units; and Sweep gas: 2 arbitrary units. QC samples were included in the batch randomly to monitor the method and instrument reproducibility in order to reduce the error associated with instrument drift.

Additional details on instrumentation, sample preparation and extraction and LC-FTMS data analysis can be found in the Supplemental Information.

### Clinical and anatomic pathology

Mice were euthanatized by carbon dioxide asphyxiation. Whole blood was collected by percutaneous cardiac puncture following euthanasia. Complete blood counts with 6-part white blood cell differential were performed on a portion of EDTA anti-coagulated whole blood (FORCYTE Autosampler 10, Oxford Science, Inc., Oxford, CT). Following the coagulation of the remaining whole blood at room temperature for 30 min, the clotted blood was centrifuged at 1500 × *g* for 5–10 min at 4 °C. Biochemical profiles were performed on serum samples (VetAxcel, Alfa Wasserman, West Caldwell, NJ). Complete postmortem evaluations were performed, and body and organ (thymus, heart, lungs, liver, spleen, kidneys, adrenals, ovaries and uterus or testes, and epididymis, brain) weights were obtained on all mice. Gross lesions were measured in three dimensions and/or weighed, diagrammed and photographed. All tissues were fixed in 10% neutral buffered formalin with the exception of the skull, sternum, vertebral column, and rear legs which were fixed in Decalcifier I (Surgipath Medical Industries, Inc., Richmond, IL) for 48 h. All tissues were processed by routine methods and embedded in paraffin wax. Sections (4 μm) were stained with hematoxylin and eosin (H&E) and evaluated with a Nikon Eclipse Ci (Nikon Instruments, Melville New York) with attached SC50 digital camera, Olympus, B and B Microscopes Limited, Pittsburgh, PA) by a veterinary pathologist certified by the American College of Veterinary Pathologists (ACVP).

### Immunohistochemistry

In all, 4 µm unstained paraffin sections were stained for the following primary antibodies: Cleaved Caspase- 3 (1:180; polyclonal rabbit [Asp175], catalog no. 9661, Cell Signaling Technology, Beverly, MA). Slides were deparaffinized in xylene and rehydrated in graded dilutions of ethanol in water. Slides were treated with Cytomation Target Retrieval Solution (pH 6.0, Dako) in a Decloaking Chamber (Biocare Medical, Concord, CA) heated to 125 °C and then cooled to 90 °C for 10 s before cooling with the lid removed for 10 min to unmask epitopes for detection of all antigens. Slides were transferred to a Dako Universal Training Center automatic immunostainer for all subsequent steps at room temperature.

Slides were treated with 3% hydrogen peroxide for 10 min, followed by serum-free protein block (Dako) for 10 min. Slides were incubated with primary antibody diluted per above concentration in antibody diluent background reducing agent (Dako). Slides were incubated with the primary antibody at 1:180 for 30 min, then rinsed and incubated with biotinylated goat anti-rabbit secondary antibody (Vector Laboratories) at 1:500 dilution for 30 min.

### Statistics

All comparisons between multiple groups were performed using a one-way or two-way analysis of variance (ANOVA) with Tukey post hoc analysis. Comparisons between values were performed using a Student’s *t* test (two-tailed). For all statistical analyses, *p* < 0.05 was considered statistically significant, unless otherwise indicated. Results are expressed as mean ± SEM. For survival analysis, Kaplan–Meier survival curves were generated with data from the day of treatment initiation to mortality. The log-rank comparison of groups (Mantel–Cox) was used to determine the statistical significance of different survival times resulting from various treatments. A multivariate statistical analysis was performed for the identified metabolite peak lists using MetaboAnalyst^®^ 3.0. Principal component analysis (PCA), partial least squares discriminant analysis (PLS-DA) as well as an orthogonal projection to latent structures discriminant analysis (OPLS-DA) was used to visualize clustering of the *Slc29a3*^+/+^ and *Slc29a3*^−/−^ samples. Unsupervised hierarchical clustering for data overview was done with the help of a heatmap generated using the *t*-test, Euclidean distance measure and Ward clustering algorithm.

### Study approval

All animal experimental procedures were conducted in strict accordance with the statutes of the Animal Welfare Act and the Guide for the Care and Use of Laboratory Animals (revised 1996). The study protocol was approved by the Institutional Animal Care and Use Committee (IACUC) of The Ohio State University, Columbus, Ohio. We have complied with the relevant ethical considerations for animal research overseen by this committee.

### Reporting summary

Further information on research design is available in the Nature Research Reporting Summary linked to this article.

## Supplementary information

Supplementary Information

Reporting Summary

Description of Additional Supplementary Files

Supplementary Data 1

Supplementary Data 2

Supplementary Data 3

## Data Availability

The data sets generated during and/or analyzed during the current study are all available within the article and its Supplementary Information files. A reporting summary for this Article is available as a Supplementary Information file. Mass-based metabolite data searches used for metabolite identification was conducted using KEGG (https://www.genome.jp/kegg/kegg1.html), HMDB (https://hmdb.ca/spectra/ms_ms/search), and LIPID MAPS (https://www.lipidmaps.org/resources/tools/index.php?tab=ms). Metabolomics data have been deposited to the EMBL-EBI MetaboLights database with the identifier MTBLS1214. Source data are provided with this paper.

## Source data

Source Data
