# Supplementary material for: Facilitative lysosomal transport of bile acids alleviates ER stress in mouse hematopoietic precursors
Source: Nat Commun. 2021 Feb 23;12:1248. doi: 10.1038/s41467-021-21451-6 (PMC7902824; doi:10.1038/s41467-021-21451-6)
Supplement: Supplementary file 1 — Supplementary Information [file 41467_2021_21451_MOESM1_ESM.pdf]

Supplemental Information

Facilitative lysosomal transport of bile acids alleviates ER stress in mouse hematopoietic precursors

Persaud et al.

Supplemental Materials and Methods

Gene expression analysis

Total RNA was extracted from cells using the RNeasy Mini Kit (Qiagen, Valencia, CA) following the manufacturer's protocol, and the RNA quality and quantity were measured using the NanoDrop™ 2000 Spectrophotometer (ThermoFisher Scientific, Waltham, MA). Quantitative PCR detection of genes was reported after reverse transcription of 1μg of total RNA using the High Capacity cDNA Reverse Transcription Kit (ThermoFisher Scientific, Waltham, MA). qPCR was performed using the PowerUP SYBR Green Master Mix (ThermoFisher Scientific, Waltham, MA) with sequence-specific primers (Sigma). ΔΔCT method (*1*) was used to determine the expression levels of genes under investigation by normalizing the Ct values to *GAPDH*. Each gene was amplified independently, and all experiments were performed in triplicate. Exon-spanning primers were designed using the primer bank web application and are described in Table S3.

Lysosome Isolation and Enrichment

Enriched lysosomes from mouse bone marrow cells were obtained using a lysosome isolation kit (LYSISO1, Sigma-Aldrich, St. Louis, MO) with differential centrifugation, followed by a density gradient centrifugation and calcium precipitation, as mentioned in the manufacturer's protocol. In brief, bone marrow cell homogenates (H) were first prepared in 1x extraction buffer using a glass Dounce homogenizer and were subjected to centrifugation for 10 min at 1,000 x*g* at 4°C to remove the nuclear pellet (NP). The post-nuclear supernatant (PNS) obtained from the earlier step was further centrifuged at 20,000 x*g* at 4°C to obtain the crude

lysosomal fraction (CLF) containing a mixture of lysosomes and other cell organelles, while the resulting supernatant fraction was collected as the cytosolic fraction (CF). The CLF was re-suspended in a minimal volume of 1x extraction buffer (0.8mL per gram mice tissue) and purified via density gradient centrifugation at 150,000 x*g* for 4 h on a multistep OptiPrep gradient (8-27%), according to the manufacturer's details. Altogether, 10 fractions of 500 μL each were collected, starting from the top of the gradient, and labeled as ELF 1-10 fractions (Enriched Lysosomal Fraction). Further purification of the ELF fractions was carried out using calcium chloride solution (250 mM), which precipitated the rough endoplasmic reticulum and mitochondria followed by its subsequent centrifugation at 5,000 x*g* at 4°C to obtain the PLF 1-10 fractions (Purified Lysosomal Fraction). In order to determine the enrichment as well as recovery of lysosomes, the homogenates as well as the different lysosomal fractions were assayed for protein concentration (using BCA protein assay) and acid phosphatase activity, a lysosomal marker enzyme (CS0740, Sigma-Aldrich, St. Louis, MO); while intactness of the lysosomes was assessed, using neutral red dye assay provided with the LYSISO1 kit. Enriched lysosomes from mouse HSCs were obtained using the Minute™ Lysosome Isolation Kit (Invent Biotechnologies, INC., Plymouth, MN) following the manufactures protocol. Then, the expression of different subcellular marker proteins in the lysosomal fractions were evaluated using Western blotting analysis by probing with specific antibodies against each marker protein like LAMP1.

ER Isolation and Enrichment

Enriched ER from mouse bone marrow cells were obtained using the ER isolation kit (ER0100) from Sigma-Aldrich (St. Louis, MO) with differential centrifugation and calcium precipitation, as mentioned in the manufacturer's protocol. In brief, bone marrow cell

homogenates (H) were first prepared in isotonic extraction buffer using a glass Dounce homogenizer and were subjected to centrifugation for 10 min at 1,000 x*g* at 4°C to remove the nuclear pellet (NP). The post-nuclear supernatant (PNS) obtained from the earlier step was further centrifuged at 12,000 x*g* at 4°C for 15min to obtain the post mitochondrial fraction (PMF) containing a mixture of ER and other cell organelles. Enriched RER was prepared by calcium chloride (8mM) precipitation of PMF followed by centrifugation for 10 min at 8,000 x*g* at 4°C. In order to determine the enrichment as well as recovery of ER, the homogenates as well as the ER fraction were assayed for protein concentration (using BCA protein assay) and NADPH cytochrome c reductase activity, an ER marker enzyme (CY0100, Sigma-Aldrich, St. Louis, MO). Also, the expression of different subcellular marker proteins in the ER fractions were evaluated using Western blotting analysis by probing with specific antibodies against each marker protein.

Immunoblots

Whole cell lysates from blood cells were made in TNE buffer supplemented with protease and phosphatase inhibitors. Total cell lysate containing 20 µg of protein were separated by SDS-PAGE on an 8% or 12% polyacrylamide gel using the Bio-Rad gel electrophoresis system following the manufacturer's protocol. Separated proteins were transferred onto PVDF membranes using the Bio-Rad Trans-Blot Turbo Transfer System. Western blot analysis was performed using GRP78 (Abcam 108615, 1:1000), GRP94 (Abcam 230842, 1:1000), p-PERK (CST 3192, 1:1000), eIF2α (CST 9722, 1:1000), p-eIF2α (CST 9721, 1:1000), IRE1 (Abcam 37073, 1:1000), p-IRE1 (Abcam 48187, 1:1000), ATF6 (Novus Biologicals 40256, 1:1000), ENT3 (ThermoFisher Scientific PA5-38039, 1:1000), and LAMP1 (CST 3243S, 1:1000) primary

antibodies, with GAPDH (CST 97166, 1:5000) as a loading control. Rabbit (Bethyl A120-201P, 1:5000) and mouse (Bethyl A90-116P, 1:5000) secondary antibodies were used, and proteins were visualized using the SuperSignal™ West Pico PLUS Chemiluminescent Substrate (ThermoFisher Scientific, Waltham, MA) on the ChemiDoc MP Imaging System from Bio-Rad. The Image Lab 5.2.1 software (Bio-Rad Laboratories, Hercules, CA) was used for immunoblot analysis. Raw blots are provided in the Data Source file.

XBP1 splicing assay

Total RNA was extracted from ST-HSCs using the RNeasy Mini Kit (Qiagen, Valencia, CA) and reverse transcribed to cDNA using the SuperScript™ IV First-Strand cDNA Synthesis System (ThermoFisher Scientific, Waltham, MA) with random hexamers and RNAse inhibitor added. The spliced (228bp) and unspliced (254bp) XBP1 cDNA segments were amplified by PCR using the Phusion Flash High Fidelity polymerase (ThermoFisher Scientific, Waltham, MA) and primers described in Table S3. Amplified PCR products were run on a 2% agarose gel stained with ethidium bromide in 1X UltraPure TBE buffer (ThermoFisher Scientific, Waltham, MA), and imaged on ChemiDoc MP Imager (Bio-Rad, Hercules, CA). All samples were assayed in duplicate with GAPDH as a loading control and subjected to densitometry measurements by AlphaView Software 3.3 (Protein Simple, Santa Clara, CA).

Metabolomic analysis-Instrumentation

Untargeted metabolomics analysis was performed on a Thermo Fisher Scientific LTQ Orbitrap XL™ Hybrid Ion Trap-Orbitrap hybrid mass spectrometer and a Waters 2795 HPLC separation module interfaced by electrospray ionization (ESI) source. For targeted metabolomics analysis, a Thermo Fisher Scientific Vanquish™ UHPLC system connected to TSQ Quantiva™ Triple Quadrupole Mass Spectrometer and interfaced with a heated electrospray ionization

(HESI-II) source. On both the instruments, data acquisition and analysis was conducted using Thermo Xcalibur™ software.

Solid phase extraction (SPE) was carried out using Visiprep™ SPE Vacuum Manifold (Sigma-Aldrich, St. Louis, MO). Mettler AE163 analytical balance was used for weighing standards and buffers. An ultra-low upright temperature freezer from New Brunswick (Eppendorf) maintained at -70 ± 10°C was used to store the plasma, urine and tissue samples. In addition, a branson 2800 ultrasonicator, Savant™ SPD121P SpeedVac and Eppendorf™ 5424R Microcentrifuge were used for study sample preparation.

Metabolomic analysis-Sample Preparation and Extraction

Initially, all the samples i.e. plasma, urine, liver and bone marrow cell samples were thawed on ice to avoid metabolite degradation. The plasma and the urine samples were vortexed and centrifuged at 21,000 x*g* prior to sample preparation. Since LT-HSC levels are low in mice, we pooled LT-HSCs from multiple mice (10-12 mice/group) within each group to obtain sufficient cells for our analysis. Quality control samples were prepared by creating a pooled sample for each matrix.

For reversed phase (RP) mode analysis, plasma and urine samples were extracted with 200 µL of cold acetonitrile: methanol (3:1, *v/v*); while 200 µL of cold methanol: water (1:1, *v/v*) was used in case of hydrophilic interaction liquid chromatography (HILIC) mode analysis. After vortex-mixing, the samples were centrifuged at 21,000 x*g* at 4°C for 10 min and 10 µL of the supernatant was injected into the LC-FTMS system.

For subcellular analysis, samples corresponding to 100 µg of lysosomal or ER protein (determined using protein concentration by BCA assay) were extracted with 100 µL of cold acetonitrile: methanol (3:1, *v/v*). The samples were vortex-mixed and then, centrifuged at 21,000 x*g*at 4°C for 10 min. The supernatant was filled in autosampler vials and volumes of 10 µL injected into the LC-FTMS system.

LC-FTMS Data Analysis

Untargeted metabolomics data processing was performed using the open source software package MZmine v.2.21 (http://mzmine.sourceforge.net/). Briefly, peak detection was performed using the following filter conditions: chromatography peak intensity signal/noise > 25, retention time tolerance: ± 0.25 min, and m/z tolerance: ± 0.04, followed by normalization using reserpine and 4-nitrophenol as internal standards for positive and negative mode respectively. Then, a chromatogram for each mass was constructed, which was deconvoluted into individual peaks using the local minimum search algorithm. To remove isotopic peaks from the peak list, the deconvoluted data were processed using the isotopic peak grouper algorithm. RANSAC algorithm was applied to align detected peaks in the different samples, generating an aligned peak list for *Slc29a3*$^{+/+}$ and *Slc29a3*$^{-/-}$ samples. The aligned peak lists containing *m/z*, retention time and peak area were exported to Microsoft® Excel and filtered based on coefficient of variation (CV) for the QC samples (< 20%) and normalized p-values for the *Slc29a3*$^{-/-}$ samples (< 0.05). Database search using HMDB, KEGG & LipidMaps was performed to establish peak identities for each of the *m/z* in this filtered aligned list with a mass tolerance set to ±10 ppm. Peak identification was based on both accurate mass and calculated formula matching. In

addition, tandem mass spectra of pure standards confirmed the identity of the metabolites examined in the transport assay.

The identified peak lists containing peak identities, sample identities and their normalized peak area intensities were imported separately into MetaboAnalyst® 3.0 for multivariate data analysis. Briefly, data normalization of each dataset was initially performed using log transformation and pareto-scaling with the missing values (if any) being replaced by a small default value. Principal component analysis (PCA), partial least squares discriminant analysis (PLS-DA) as well as orthogonal projection to latent structures discriminant analysis (OPLS-DA) was used to visualize clustering of the *Slc29a3*$^{+/+}$ and *Slc29a3*$^{-/-}$ samples along with the QCs. Unsupervised hierarchical clustering for data overview of each of the datasets was accomplished with help of a heatmap generated using t-test, Euclidean distance measure and Ward clustering algorithm. Cross-validated PLS-DA was used to obtain a list of discriminating metabolites from each dataset, ranked according to their Variable Importance in Projection (VIP) scores, with VIP >1.0 being considered relevant for group discrimination.

***Xenopus* Oocyte Transport Assay**

The construction of pOX-Δ36hENT3 Xenopus expression vector and *in vitro* transcription and expression of Δ36hENT3 in Xenopus oocytes were described earlier (2, 3). Transport of radiolabeled substrates was conducted as described before (*4*). Briefly, 50 nanoliters (400–800 ng/μl) of Δ36hENT3 mRNA were injected into defolliculated oocytes. The injected oocytes were incubated at 15 °C for 24 h before performing transport assays. Uptake of radiolabeled substrates (^{3}H-adenosine or ^{3}H-BAs; 0.02 μM) supplemented in combination with unlabeled substrates (to a final concentration of 100 μM; for estimation of kinetic parameters)

was measured after 30 min of incubation in transport buffer (100 mM NaCl, 2 mM KCl, 1 mM $CaCl_2$, 1 mM $MgCl_2$, and 10 mM HEPES, pH 5.5) at room temperature. Uptake was terminated by washing oocytes 3 times with arrest buffer (20 mM Tris-HCl, 3 mM K_2HPO4, 1 mM $MgCl_2 \cdot 6H2O$, 2 mM $CaCl_2$, 5 mM glucose, 130 mM N-methyl-D-glucamine) containing 20 mM uridine. Individual oocytes were shaken overnight in 10% SDS for complete dissolution, and then the radioactivity was quantified by Beckman liquid scintillation counter. Data represent the average ± S.E. (n = 5–10 oocytes). The results were normalized for experimental variations and analyzed using Graphpad prism software. The kinetic parameters, K_m and V_{max} were calculated by fitting the data of uptake rate of identified metabolites to the Michaelis-Menten equation. A representative experiment from 3–5 independent experiments is presented.

The mass spectrometric validation of the transport of all unlabeled metabolites was performed with oocytes according to the method reported earlier. Briefly, the oocytes obtained after treatment with wash buffer was homogenized with 200 µL of extraction buffer (50% methanol containing 0.1% acetic acid) and spiked with cholic acid-d4 (1µg/mL) and glycocholic acid-d4 (1µg/mL) as internal standards. After incubation at 4 ^{o}C for 24 h, samples were centrifuged at 20,000g for 10 min and supernatants collected. Samples (10 µL) were injected into the Thermo Scientific Vanquish UPLC system (Waltham, MA) interfaced with Thermo Scientific TSQ Quantiva triple-stage quadrupole mass spectrometer (Waltham, MA) equipped with H-ESI ion source. MS detection was carried out in positive and negative ionization modes and the transition monitored for each metabolite were reported in table (Table S2 and S3). The operational mass spectrometric parameters included capillary voltage: 4.5 kV; sheath gas: 35 arbitary units; auxiliary gas: 10 arbitary units; sweep gas: 2 arbitary units; ion transfer tube temperature: 350°C; vaporizer temperature: 450°C; Dwell time: 50 ms per transition; Collision

Energy (CE): 5 V and collision induced dissociation (CID) gas: 1.5 mtorr. Chromatographic separation was carried out on a Kinetex C_{18} (100 X 2.1mm; 1.7 μm particle size) from Phenomenex (Torrance, CA). The mobile phase consisted of solvent A: Water (0.1 % formic acid) and Solvent B: Acetonitrile (0.1 % formic acid). The flow rate was set at 0.5 mL/min and the gradient program used was 0 min: 10% B; 2 min: 10% B; 5 min: 22% B; 20 min: 95% B; 24 min 95%B; 25 min: 10% B; 30 min: 10% B. The total run time was 30 min per sample and the auto-sampler was maintained at 4°C throughout the analysis.

ENT3 Overexpression in HEK293 cells and HEK293 Transport Assay

HEK293 cells were seeded at a density of 1.0 x 10^{5} cells/well in 24-well plates. After 24 hours, cells were transfected with a plasmid encoding ENT3 (pEYFP-ENT3) described previously (*2*,*3*) using Lipofectamine 3000 following the manufacture's protocol. The culture media was removed 24 hours post-transfection, and transport buffer (20 mM Tris-HCl, 3 mM dipotassium phosphate, 1 mM magnesium, 2 mM calcium chloride, and 5 mM glucose) containing 1 μM cholic acid (CA; including 0.02 μM ^{3}H-cholic acid) was added to each cell type for 0.5, 3, 12 or 24 hours. Uptake was terminated by washing the cells with arrest buffer as previously described (3), and radioactivity was quantified using the Beckman liquid scintillation counter after cell lyses with 10% SDS. Protein was quantified using the Pierce™ BCA Protein Assay Kit (23225), and DPM values were normalized to protein content and presented as pmol/mg of protein.

3D QSAR Analysis

3D QSAR models were built through the 3D-QSAR online server (http://www.3d-qsar.com/). The transport data relative to adenosine of 21 BA compounds was employed as the training dataset to build 3D QSAR models. 2D chemical structures of BAs were converted to 3D structures and subsequently minimized using Chem3D 16.0 (PerkinElmer/CambridgeSoft, UK). 30 conformations for each BA molecule were generated using Py-ConfSearch, and all conformations were aligned to a cholic acid conformation with the RDKit method. After the alignment, molecular interaction field (MIF) point based descriptors were used for building 3D-QSAR models with known BA transport values with Py-CoMFA. The MIFs were generated using C.3, C.cat, N.3, O.3, H, and C.2 as atom probes. Both electrostatic and steric interaction fields were used. The sample point maximum distance was set to 1.0 Å. The experimental transport coefficients relative to adenosine (TC_{ade}) were converted to their positive-logarithmic scale by using the formula: $pTC_{ade} = -\log (TC_{ade})$ and defined as the dependent variable. The partial least square (PLS) regression method was used through the 3D-QSAR Py-CoMFA module. The maximum number of components was set to 8, and Y scrambles were set to 10. The derived QSAR models were assessed by regression coefficient (r^2) and validated by leave-one-out (LOO) cross-regression coefficient (q^2). The robustness of the derived 3D-QSAR models were validated by the leave-one-out (LOO) technique.

Lentiviral gene transfer in mouse LSKs and HSCs

Lentiviruses harboring ENT3 or RFP were generated in HEK293T (packaging) cells by transfecting the target plasmids (Applied Biological Materials Inc., BC, Canada) and packaging plasmids (Trans-Lentiviral ORF Packaging Kit, Dharmacon, IL, USA), using the calcium phosphate transfection method as per the manufacturer’s instructions. For viral transduction,

HSPCs were transduced with lentivirus (10^8 transduction units) in the presence of 8 μg/ml polybrene in HSC medium on polypropylene tubes pre-coated with 1% bovine serum albumin (BSA), centrifuged at 1500 x*g* for 3 h at 32°C and incubated for 24 h without centrifugation.

Colony-forming cell (CFC) assay

HSCs were plated at a concentration of 3.0×10^5 in semisolid MethoCult media containing a cocktail of cytokines designed for burst-forming unit-erythroid (STEMCELL Technologies, SF M3436) and colony-forming unit-erythroid (STEMCELL Technologies, M3334) enumeration following the manufacture's protocol. Burst-forming unit-erythroid (BFU-E) and colony-forming unit-erythroid (CFU-E) were quantified after 14 days with and without salubrinal treatment (10 mM) using a Bright-field microscope.

References

1. Schmittgen, T. & Livak, K. Analyzing real-time PCR data by the comparative CT method. *Nature Protocols 3*, 1101-1108 (2008).
2. Govindarajan, R., Leung, G.P.H., Zhou, M., Tse, C., Wang, J., and Unadkat, J.D. Facilitated mitochondrial import of antiviral and anticancer nucleoside drugs by human equilibrative nucleoside transporter-3. *American Journal of Physiology-Gastrointestinal and Liver Physiolog*y 296, G910-G922(2009).
3. Kang, N., Jun, A. H., Bhutia, Y. D., Kannan, N., Unadkat, J. D., and Govindarajan, R. Human equilibrative nucleoside transporter-3 (hENT3) spectrum disorder mutations impair nucleoside transport, protein localization, and stability. *J. Biol. Chem*. 285, 28343–28352 (2010).
4. Rahman, M., Askwith, C. & Govindarajan, R. Molecular determinants of acidic pH-dependent transport of human equilibrative nucleoside transporter 3. *Journal of Biological Chemistry* 292, 14775-14785 (2017).

Supplemental Figures

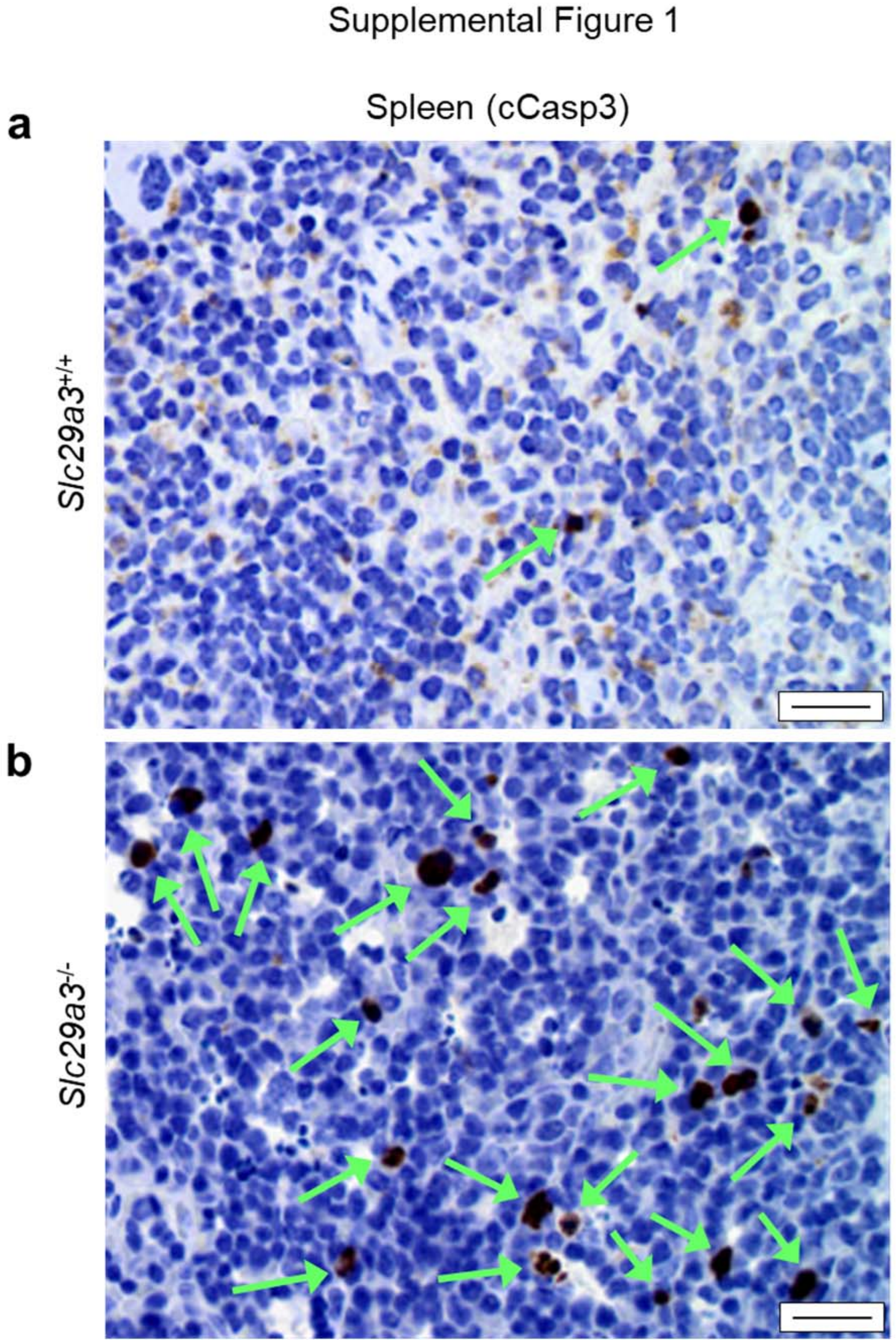

Supplemental Fig 1. *Slc29a3*$^{-/-}$ mice have increased splenic apoptosis. **(a)** Representative histological image of *Slc29a3*$^{+/+}$ spleen at 16 weeks immunostained for cleaved caspase 3 (cCasp3; *brown*) showing few hematopoietic cells undergoing apoptosis as indicated by green arrows. (40x; Scale bar = 20 mm). One representative image from three independent experiments is shown. **(b)** Representative histological image of *Slc29a3*$^{-/-}$ spleen at 16 weeks immunostained for cleaved caspase 3 (cCasp3; *brown*) showing numerous hematopoietic cells undergoing apoptosis as indicated by green arrows. (40x; Scale bar = 20 mm). One representative image from three independent experiments is shown. Bone marrow sections were not amenable for immunostaining due to the decalcification process.

Supplemental Figure 2

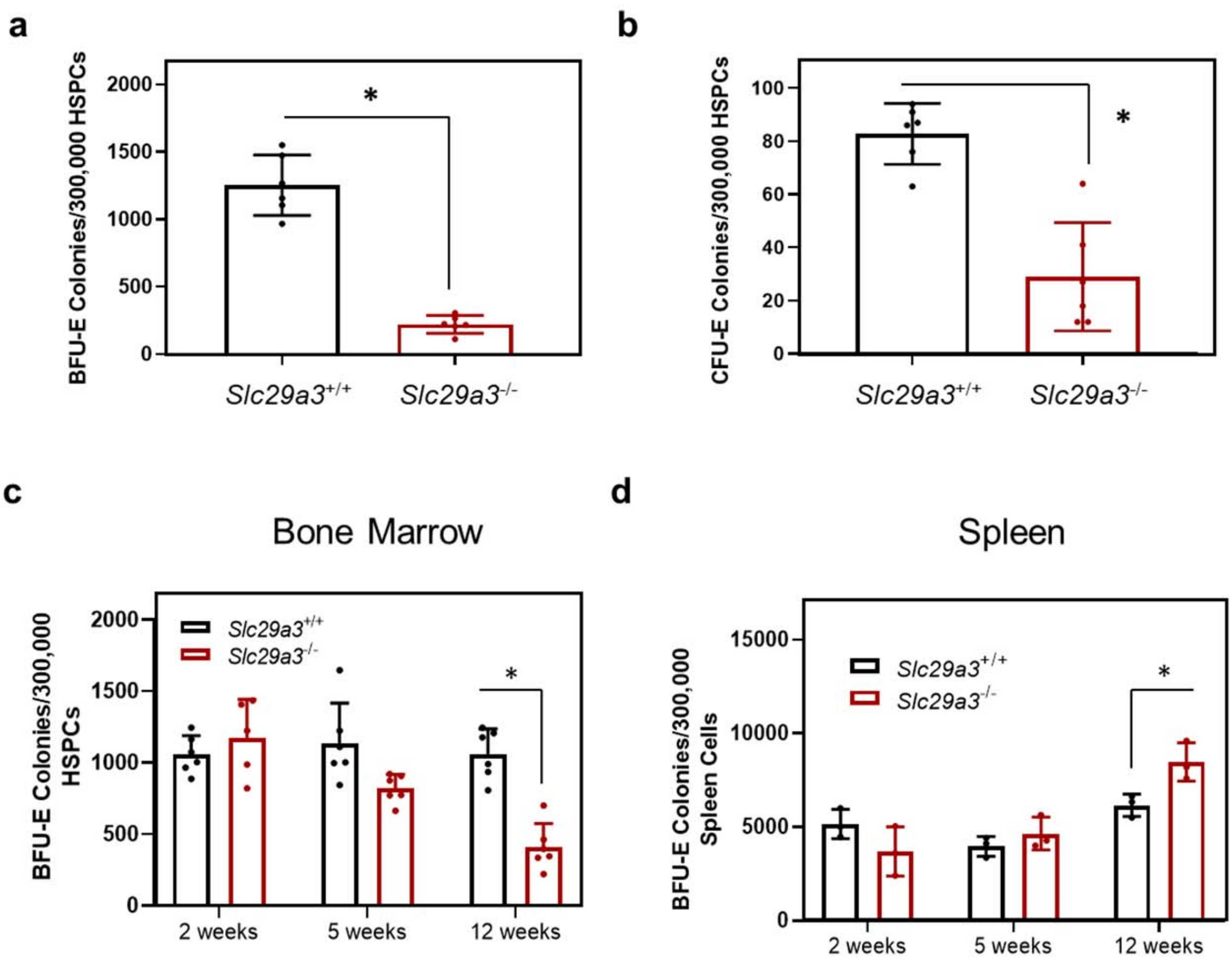

Supplemental Fig. 2. The loss of ENT3 decreases *Slc29a3*$^{-/-}$ mouse HSPC differentiation into erythroid precursors. **(a)** Colony enumeration of burst forming unit-erythrocytes (BFU-E) produced from *Slc29a3*$^{+/+}$ (*black*) and *Slc29a3*$^{-/-}$ (*red*) HSPCs from 12-weeks-old mice (*n* = 6 mice/group , mean ± SEM, *p < 0.05 by two-tailed t-test). **(b)** Colony enumeration of colony forming unit-erythrocytes (CFU-E) produced from *Slc29a3*$^{+/+}$ (*black*) and *Slc29a3*$^{-/-}$ (*red*) HSPCs from 12-week-old mice (*n* = 6 mice/group, mean ± SEM, *p < 0.05 by two-tailed t-test). **(c)** Colony enumeration of BFU-E produced from *Slc29a3*$^{+/+}$ (*black*) and *Slc29a3*$^{-/-}$ (*red*) HSPCs isolated from 2, 5, and 12-week-old mice (*n* = 6 mice/group, mean ± SEM, *p < 0.05 by two-tailed t-test). **(d)** Colony enumeration of BFU-E produced from *Slc29a3*$^{+/+}$ (*black*) and *Slc29a3*$^{-/-}$ (*red*) spleen cells isolated from 2, 5, and 12-week-old mice (*n* = 3 mice/group, mean ± SEM, *p < 0.05 by two-tailed t-test).

Supplemental Figure 3

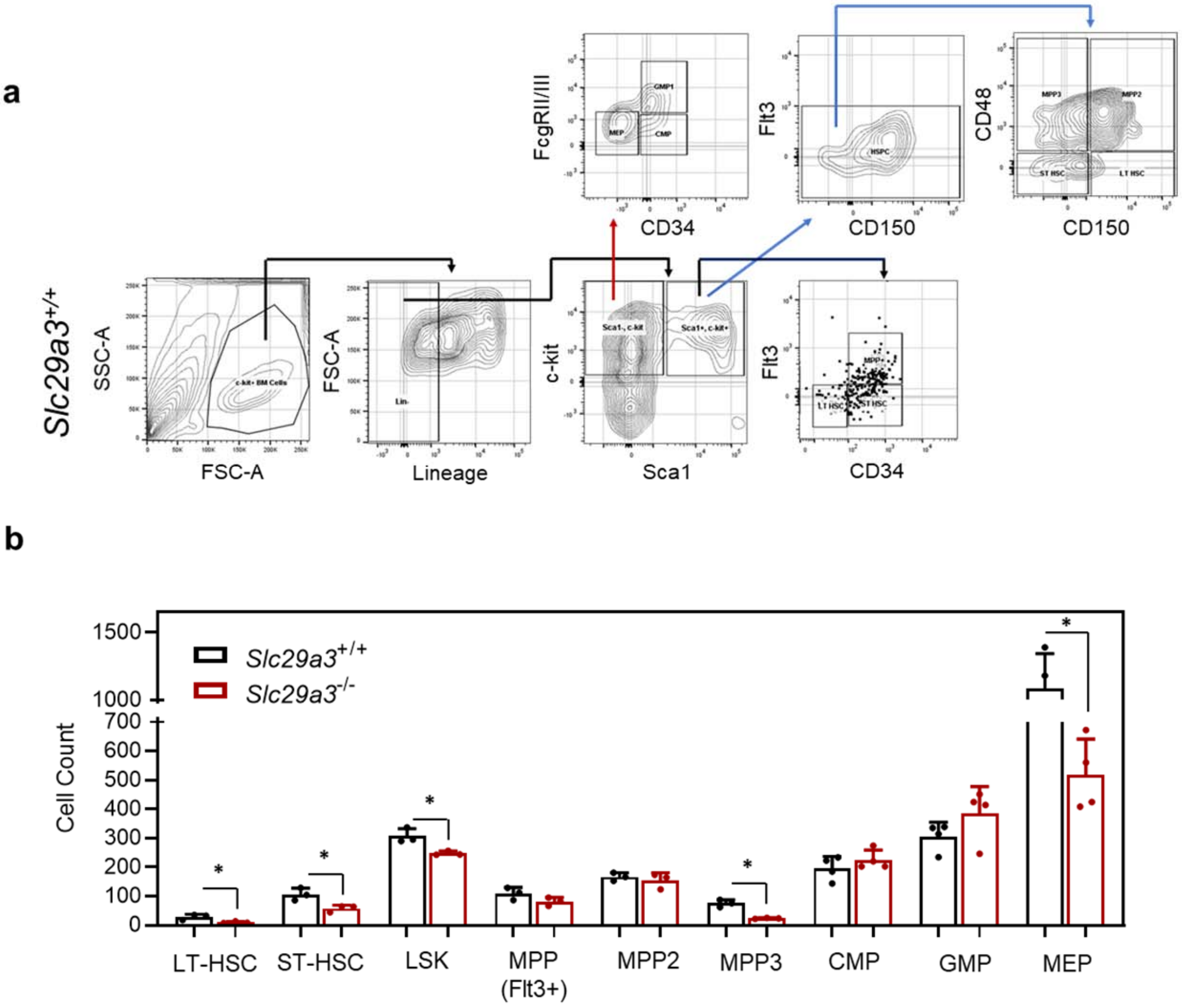

Supplemental Fig. 3. The loss of ENT3 alters the cell count of HSPC subpopulations in *Slc29a3*$^{-/-}$ mice. **(a)** Representative gating strategy for HSPC subpopulations (LT-HSC, ST-HSC, LSK+, MPP FLT3+, MPP2, MPP3, CMP, GMP, and MEP) from *Slc29a3*$^{+/+}$ and *Slc29a3*$^{-/-}$ mice presented in supplemental fig. S9B and S9C. **(b)** HSPC subpopulations cell count derived from analytical cytometry analysis of c-kit enriched bone marrow cells isolated from 12 week old *Slc29a3*$^{+/+}$ (*black*) and *Slc29a3*$^{-/-}$ (*red*) mice (n = 3 mice/group, mean ± SEM, *$p < 0.05$ by two-tailed t-test).

Supplemental Figure 4

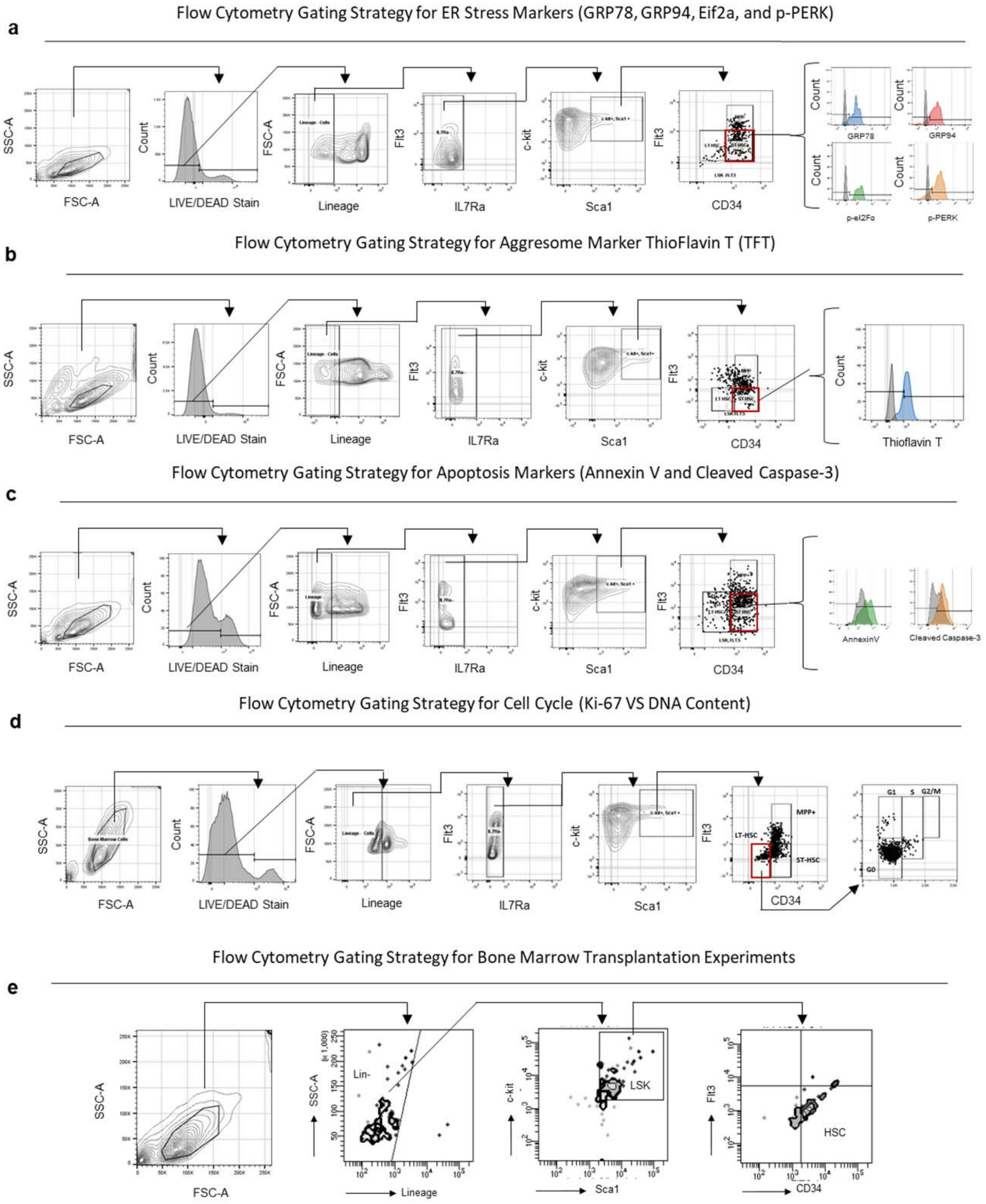

Supplemental Fig. 4. Flow cytometry gating strategies for ER stress, aggresome, apoptosis, and cell cycle markers. **(a)** ER stress marker (GRP78, GRP94, Eif2a, and p-PERK) gating strategy for HSPC populations (LSK, LSK FLT3-, ST-HSCs and LT-HSCs) from *Slc29a3*$^{+/+}$ and *Slc29a3*$^{-/-}$ mice presented in fig. 2a and fig. S6 and S7. **(b)** Aggresome marker (TFT) gating strategy for HSPC populations (LSK, LSK Flt3-, ST-HSCs and LT-HSCs) from *Slc29a3*$^{+/+}$ and *Slc29a3*$^{-/-}$ mice presented in fig 2a and fig. S6 and S7. **(c)** Apoptosis marker (Annexin V and Cleaved Caspase-3) gating strategy for HSPC populations (LSK, LSK FLT3-, ST-HSCs and LT-HSCs) from *Slc29a3*$^{+/+}$ and *Slc29a3*$^{-/-}$ mice presented in fig. 2a and fig. S6 and S7. **(d)** Cell cycle (Ki-67 and Hoecht 33342) gating strategy for HSPC subpopulations from *Slc29a3*$^{+/+}$ and *Slc29a3*$^{-/-}$ mice presented in fig 2b. **(e)** Gating strategy for lineage depleted HSC populations (Lin^{-}, c-kit^{+}, Sca1^{+}, FLT3, CD34^{+}) from *Slc29a3*$^{+/+}$ and *Slc29a3*$^{-/-}$ transplant mice presented in fig 3.

Supplemental Figure 5

a

Averaged RAW Ct Values							
		LT-HSC	ST-HSC	MPP (Flt3$^+$)	LSK	c-kit$^+$ BM	BM
Slc29a3	***Slc29a3$^{+/+}$***	26.5	29.3	27.6	25.3	21.8	18.2
	Slc29a3$^{-/-}$	31.1	32.0	32.3	32.3	30.7	33.2
GAPDH	***Slc29a3$^{+/+}$***	21.3	23.3	24.6	23.3	16.1	12.5
	Slc29a3$^{-/-}$	22.0	22.5	22.5	21.2	18.2	14.6

b

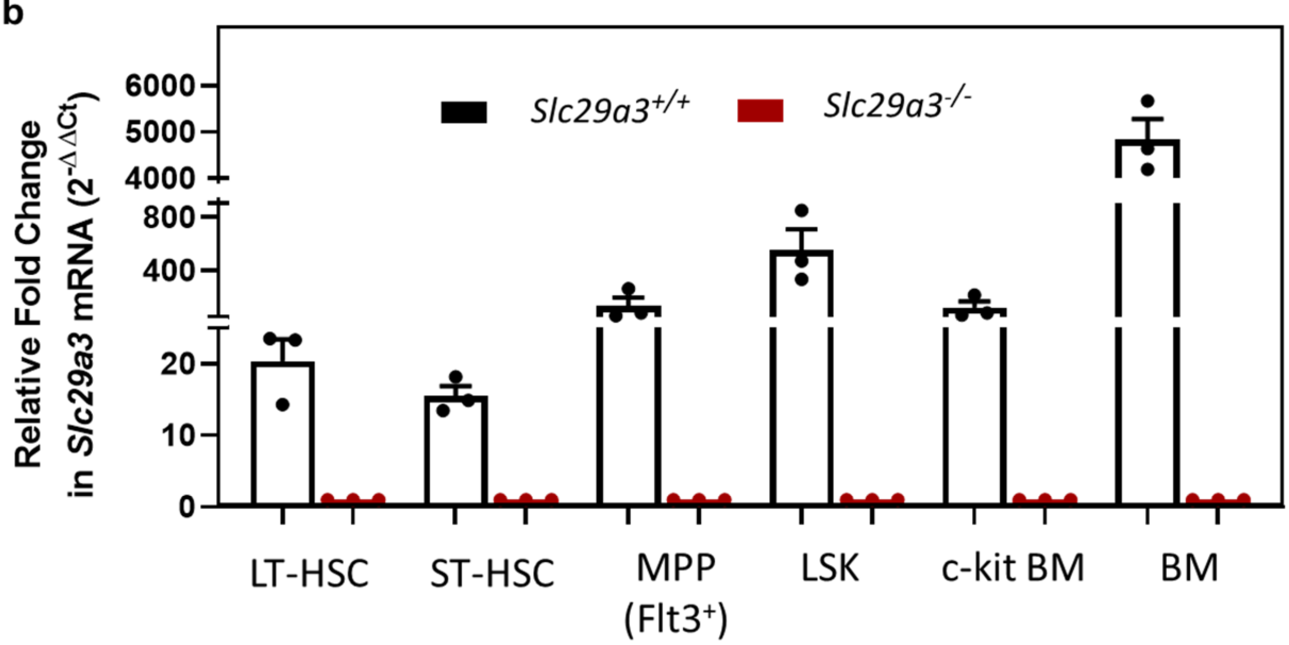

c

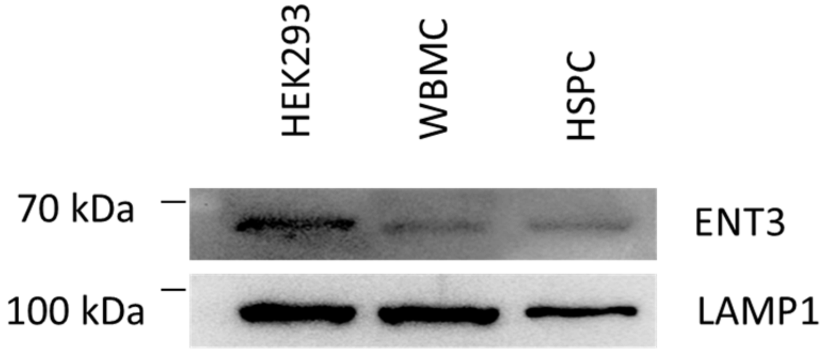

Supplemental Fig. 5. *Slc29a3* expression in HSPC subpopulations. **(a)** Averaged raw Ct values after amplification of *Slc29a3* and *GAPDH* from LT-HSCs, ST-HSCs, MPP+, LSK, c-kit bone marrow (BM), and crude BM cells isolated from 5-week-old *Slc29a3*$^{+/+}$ and *Slc29a3*$^{-/-}$ mice. **(b)** qPCR data normalized to GAPDH and *Slc29a3*$^{+/+}$ mouse gene expression presented as relative fold change (2-ΔΔCt) for each cell population from *Slc29a3*$^{+/+}$ (*black*) and *Slc29a3*$^{-/-}$ (*red*) mice (n = 3 mice/group, mean ± SEM). **(c)** Representative immunoblots of lysosomal ENT3 from HEK293 (positive control), bone marrow cells, and HSPCs from *Slc29a3*$^{+/+}$ mice. Lysosomal fractionation was confirmed with co-fractionation with the lysosomal membrane marker LAMP1. One representative blot from three independent experiments is shown.

Supplemental Figure 6

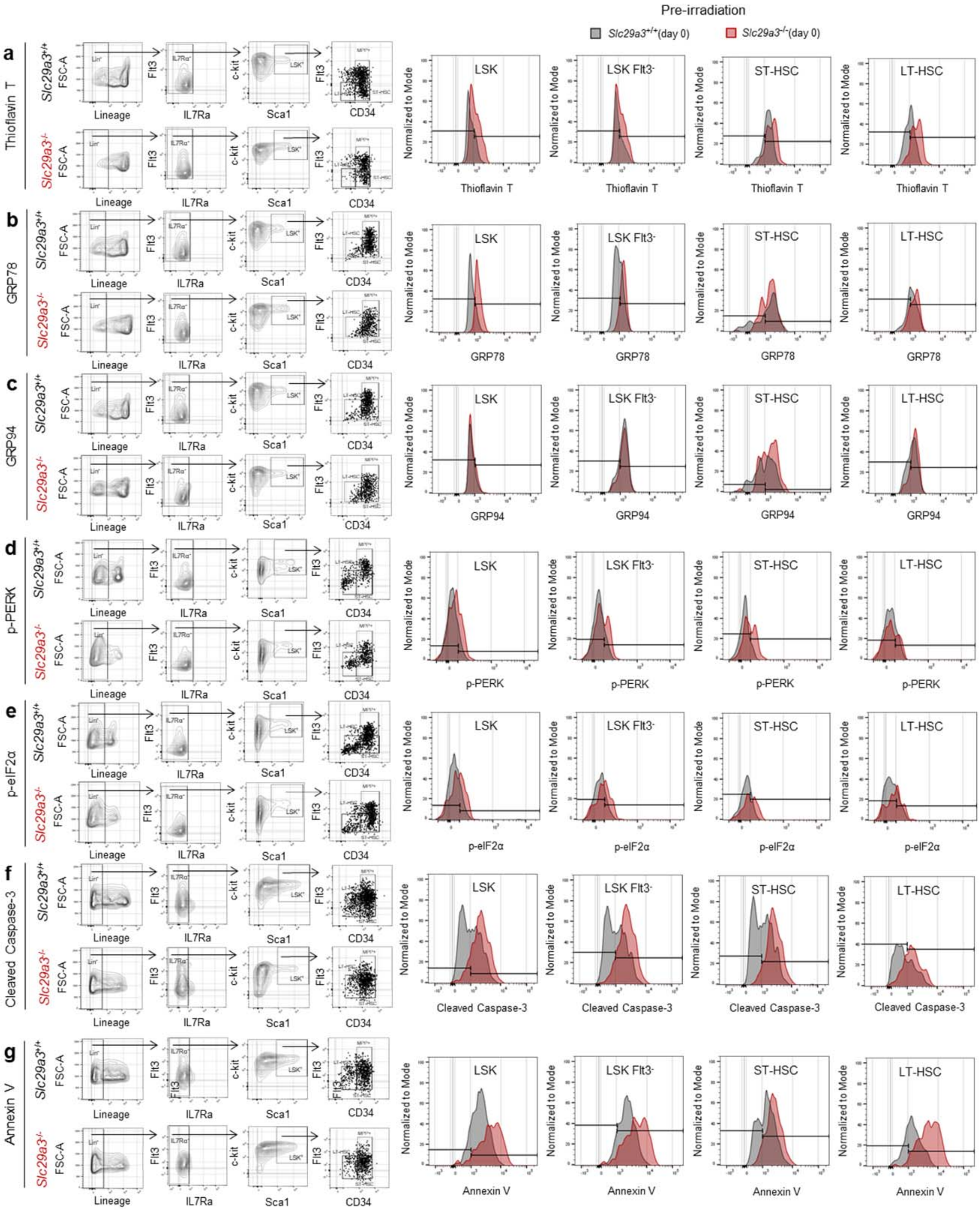

Supplemental Fig. 6. Flow cytometry profiling of ER stress, apoptosis, and aggresomal markers pre-irradiation. LSK, LSK FLT3-, ST-HSCs and LT-HSCs were examined for **(a)** Thioflavin T (ThT) stained unfolded proteins (ThT^{+}), **(b)** GRP78, **(c)** GRP94, **(d)** p-eIF2α, **(e)** p-PERK, **(f)** Cleaved Caspase-3, and **(g)** Annexin V in 8-week old *Slc29a3*$^{+/+}$ and *Slc29a3*$^{-/-}$ mice before irradiation. Gating strategy for HSPC populations (LSK, LSK Flt3-, ST-HSCs and LT-HSCs) from *Slc29a3*$^{+/+}$ and *Slc29a3*$^{-/-}$ mice followed by a histogram overlay of *Slc29a3*$^{+/+}$ (gray) and *Slc29a3*$^{-/-}$ (red) are presented for each biomarker.

Supplemental Figure 7

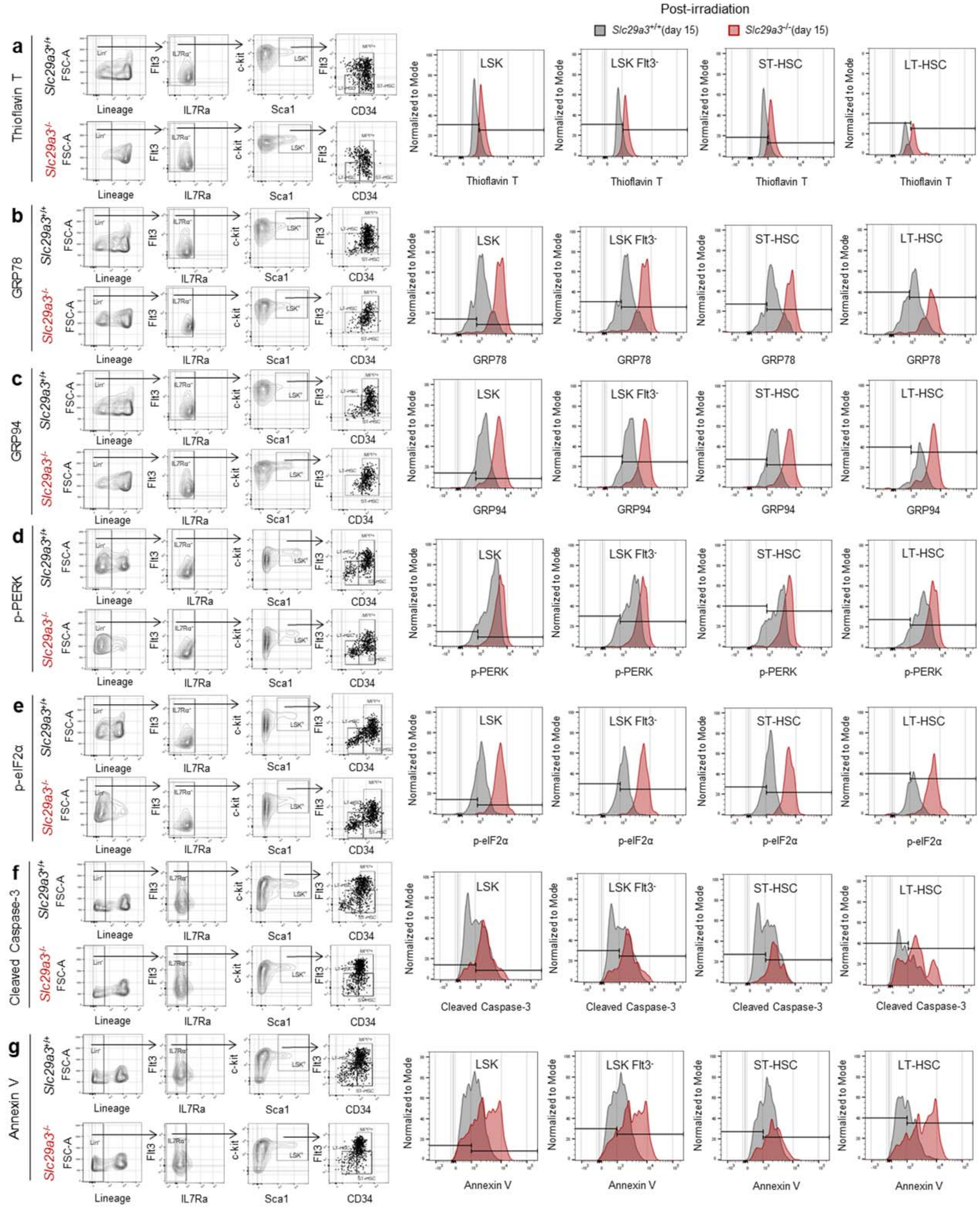

Supplemental Fig. 7. Flow cytometry profiling of ER stress, apoptosis, and aggresomal markers post-irradiation. LSK, LSK FLT3-, ST-HSCs and LT-HSCs were examined for **(a)** Thioflavin T (ThT) stained unfolded proteins (ThT^{+}), **(b)** GRP78, **(c)** GRP94, **(d)** p-eIF2α, **(e)** p-PERK, **(f)** Cleaved Caspase-3, and **(g)** Annexin V in 8-week old *Slc29a3*$^{+/+}$ and *Slc29a3*$^{-/-}$ mice 15 days after sub-lethal irradiation (6.5 Gy). Gating strategy for HSPC populations (LSK, LSK Flt3-, ST-HSCs and LT-HSCs) from *Slc29a3*$^{+/+}$ and *Slc29a3*$^{-/-}$ mice followed by a histogram overlay of *Slc29a3*$^{+/+}$ (gray) and *Slc29a3*$^{-/-}$ (red) are presented for each biomarker.

Supplemental Figure 8

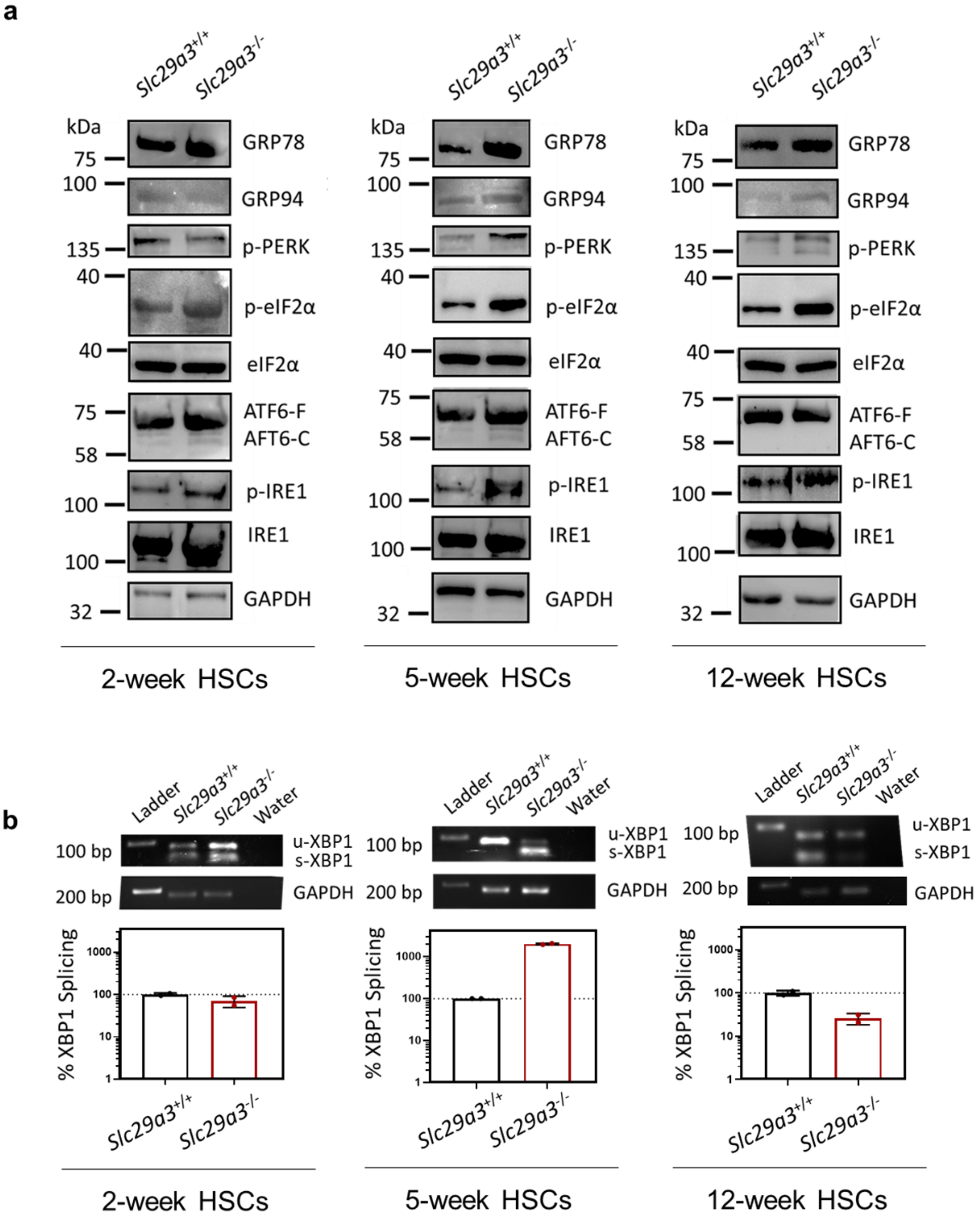

Supplemental Fig. 8. The loss of ENT3 activates the unfolded protein response in *Slc29a3*$^{-/-}$ mouse HSCs. **(a)** Representative immunoblots of proteins from the PERK, ATF6, and IRE1 arm of the unfolded protein response in *Slc29a3*$^{+/+}$ and *Slc29a3*$^{-/-}$ mouse ST-HSCs at 2, 5, and 12 weeks of age. One representative blot from three independent experiments is shown for each experimental condition. **(b)** XBP1 splicing assay of cDNA extracted from *Slc29a3*$^{+/+}$*(black)* and *Slc29a3*$^{-/-}$ *(red)* mouse ST-HSCs at 2, 5, and 12 weeks of age. RT-PCR of sXBP1 and uXBP1 was run on a 3% agarose gel and visualized with ethidium bromide followed by XPB1 splicing quantification (*n* = 2 mice/group).

Supplemental Figure 9

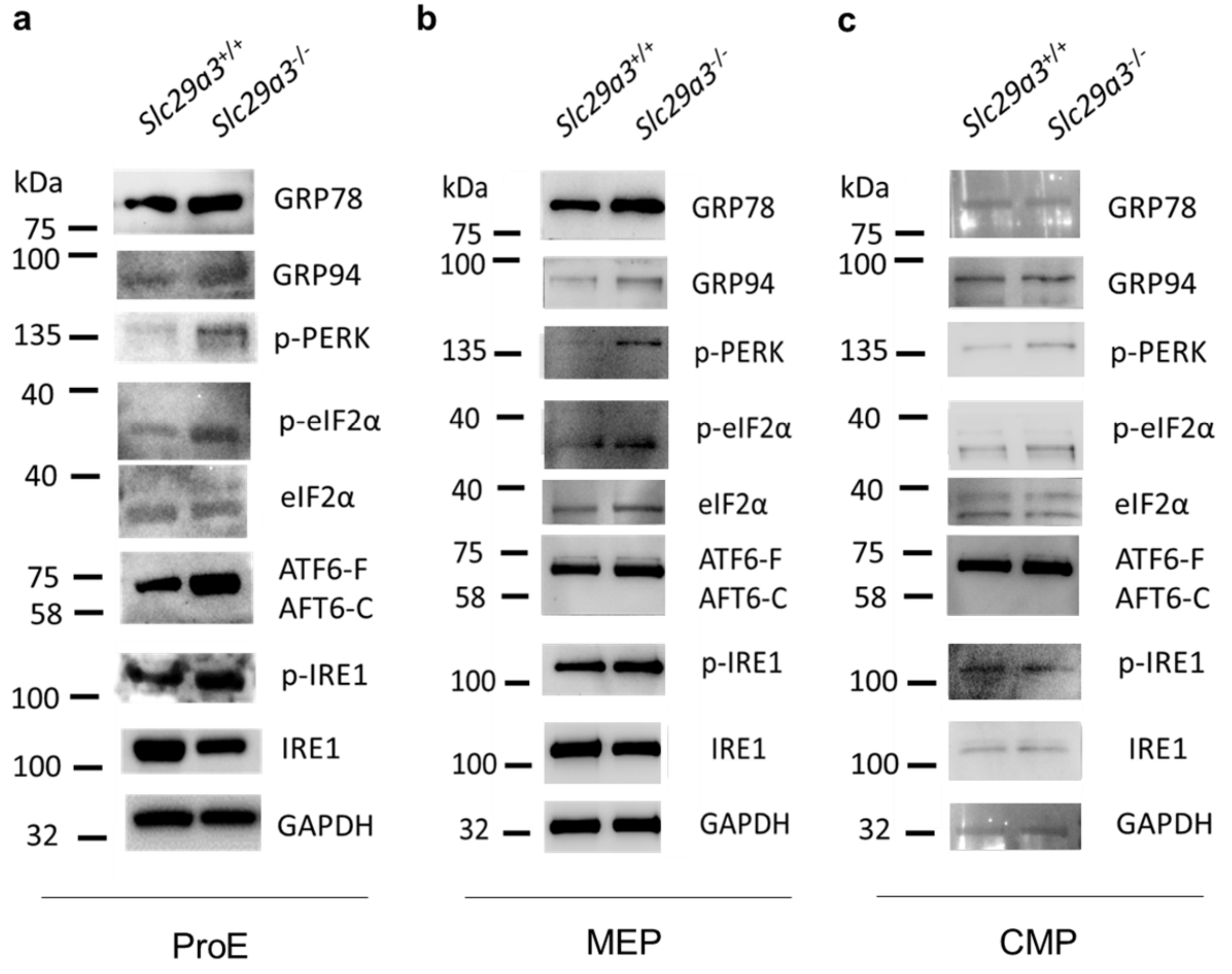

Supplemental Fig. 9. ENT3 loss activates the unfolded protein response in *Slc29a3*$^{-/-}$ mouse erythroid precursors and progenitors. **(a)** Representative immunoblots of proteins from the PERK, ATF6, and IRE1 arm of the unfolded protein response in *Slc29a3*$^{+/+}$ and *Slc29a3*$^{-/-}$ mouse ProE cells at 12 weeks of age. **(b)** Representative immunoblots of proteins from the PERK, ATF6, and IRE1 arm of the unfolded protein response in *Slc29a3*$^{+/+}$ and *Slc29a3*$^{-/-}$ mouse MEP cells at 12 weeks of age. **(c)** Representative immunoblots of proteins from the PERK, ATF6, and IRE1 arm of the unfolded protein response in *Slc29a3*$^{+/+}$ and *Slc29a3*$^{-/-}$ mouse CMP cells at 12 weeks of age. One representative blot from three independent experiments is shown for each experimental condition.

Supplemental Figure 10

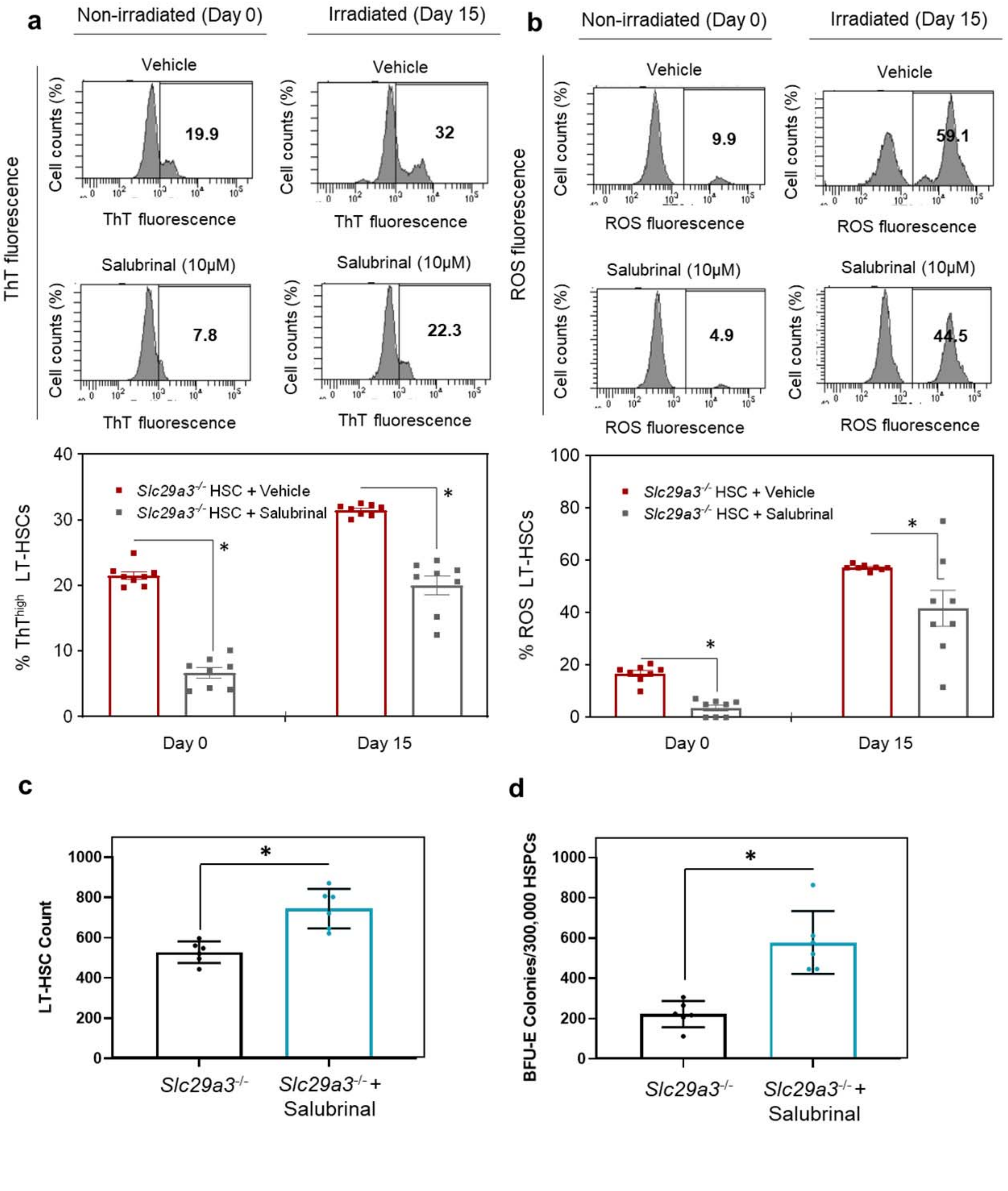

Supplemental Fig. 10. Salubrinal treatment of *Slc29a3*$^{-/-}$ mice reduces aggresomes and reactive oxygen species. **(a)** LT-HSCs isolated from *Slc29a3*$^{-/-}$ mice have reduced Thioflavin T (ThT) fluorescence with (*grey*) and without (*red*) salubrinal treatment pre- and post-irradiation. Data represent mean±SEM (n=8 mice/group, *p < 0.05 by two-tailed t-test). **(b)** LT-HSCs isolated from *Slc29a3*$^{-/-}$ mice have reduced reactive oxygen species (ROS) with (*grey*) and without (*red*) salubrinal treatment pre- and post-irradiation. Data represent mean±SEM (n=8 mice/group, *p < 0.05 by two-tailed t-test). **(c)** LT-HSC count after treatment of *Slc29a3*$^{-/-}$ HSPCs with (*black*) or without (*blue*) salubrinal (10 mM) (*n* = 6 mice/group, mean ± SEM, *p < 0.05 by two-tailed t-test). **(d)** Colony enumeration of burst forming unit-erythrocytes (BFU-E) produced from *Slc29a3*$^{+/+}$ and *Slc29a3*$^{-/-}$ HSPCs after vehicle (DMSO)(*black*) or salubrinal (10 mM) (*blue*) treatment for 24 hours (*n* = 6 mice/group, mean ± SEM, *p < 0.05 by two-tailed t-test).

Supplemental Figure 11

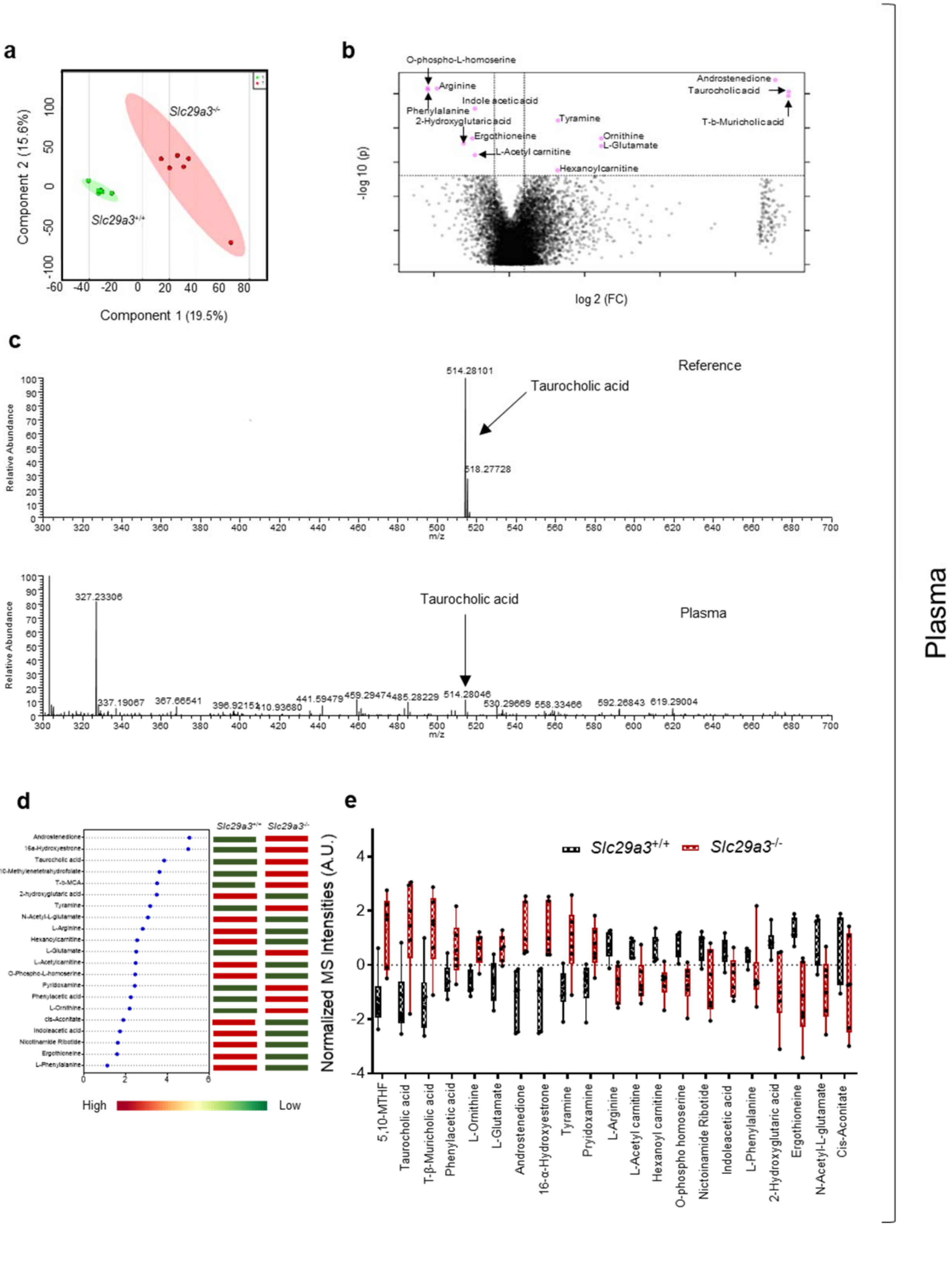

Supplemental Fig. 11. Analysis of differentially produced metabolites in *Slc29a3*$^{-/-}$ plasma. **(a)** Projection to latent structure-discriminant analysis (PLS-DA) score plot constructed based on metabolic profiles of *Slc29a3*$^{+/+}$ and *Slc29a3*$^{-/-}$ plasma samples (*green, Slc29a3*$^{+/+}$; *red, Slc29a3*$^{-/-}$). **(b)** Volcano plots showing metabolite profiles of *Slc29a3*$^{+/+}$ compared with *Slc29a3*$^{-/-}$ plasma samples. Dotted lines along x axis represent ± log2(2) fold change and the dotted line along y axis represents –log10(0.05). *pink*, differential metabolites; *black*, other metabolites. **(c)** Representative reference standard and plasma sample MS2 spectra used for identification of the metabolite taurocholic acid. **(d)** Variable in projection (VIP) plot illustrating the 21 significantly altered metabolites in *Slc29a3*$^{-/-}$ mice plasma ranked on the basis of PLS-DA modelling. The colored boxes represent the relative levels of the metabolites in each group. *red*, high; *green*, low. **(e)** Box-whisker plot representation of 21 significantly altered metabolites in *Slc29a3*$^{-/-}$ mice plasma samples. Values, Mean ± S.E.M; n=6 mice/group, *, $p<0.05$ by two-tailed t-test comparing *Slc29a3*$^{+/+}$ and *Slc29a3*$^{-/-}$ and fold change >2.0 w.r.t *Slc29a3*$^{+/+}$**.** Box plots represent the median (middle line), 25th, and 75th percentile (box), while the whiskers span from the minimum to the maximum value.

Supplemental Figure 12

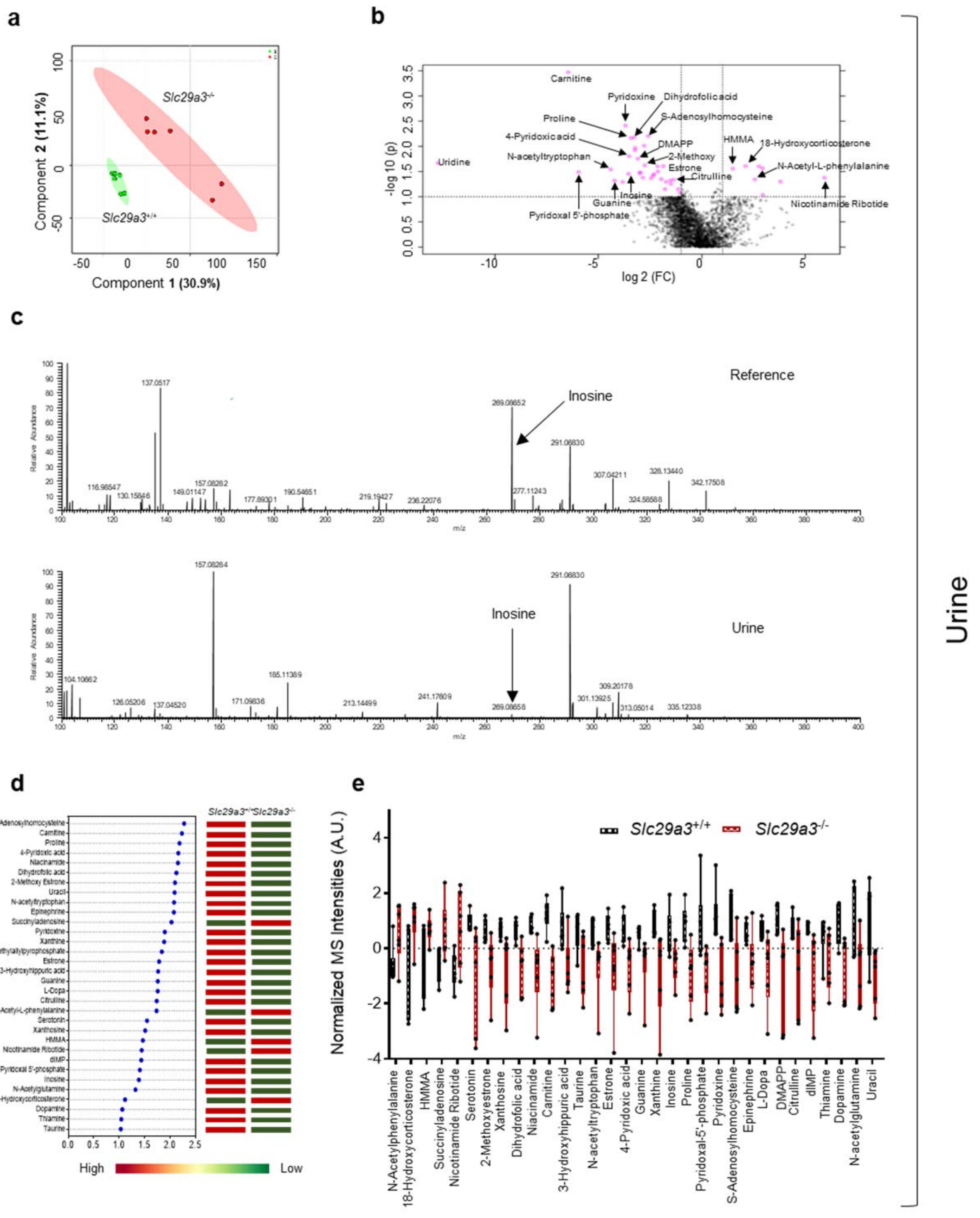

Supplemental Fig. 12. Analysis of differentially produced metabolites in *Slc29a3*$^{-/-}$ urine. **(a)** PLS-DA score plot constructed based on metabolic profiles of *Slc29a3*$^{+/+}$ and *Slc29a3*$^{-/-}$ urine. **(b)** Volcano plot for comparison of *Slc29a3*$^{+/+}$ and *Slc29a3*$^{-/-}$ urine. **(c)** Representative reference standard and urine sample MS2 spectra used for identification of the metabolite inosine. **(d)** VIP plot for 32 significantly altered metabolites in *Slc29a3*$^{-/-}$ urine. **(e)** Box-whisker plot representation of for 32 significantly altered metabolites in *Slc29a3*$^{-/-}$ mice urine. Values, Mean ± S.E.M; n=6 mice/group, *, $p<0.05$ by two-tailed t-test comparing *Slc29a3*$^{+/+}$ and *Slc29a3*$^{-/-}$ and Fold change >2.0 w.r.t *Slc29a3*$^{+/+}$. Box plots represent the median (middle line), 25th, and 75th percentile (box), while the whiskers span from the minimum to the maximum value.

Supplemental Figure 13

a

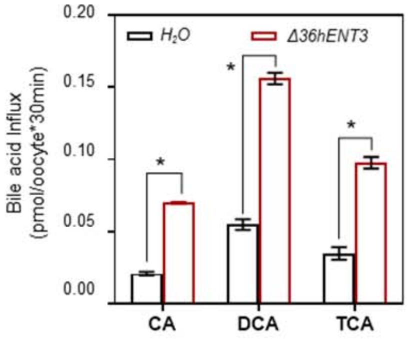

b

Bile acid	Exp	Fit	Pred	Err Pred
TCA	3.042	3.048	1.483	-1.559
DCA	2.101	2.061	1.694	-0.407
CA	2.228	2.366	2	-0.228
UDCA	2.173	2.118	1.39	-0.783
TUDCA	1.795	1.714	1.39	-0.405
GCA	1.738	1.73	1.416	-0.322
GUDCA	1.422	1.435	1.315	-0.107
HCA	1.492	1.321	1.127	-0.365
CDCA	1.223	1.197	1.18	-0.043
MCA	1.189	1.268	1.592	0.403
TMCA	1.305	1.34	1.528	0.223
**Bile acid**	**Exp**	**Fit**	**Pred**	**Err Pred**
GCDCA	1.324	1.361	1.406	0.082
LCA	1.139	1.125	1.274	0.135
DHCA	1.145	1.146	1.279	0.134
HDCA	1.057	1.132	1.563	0.506
THDCA	1.039	1.059	1.156	0.117
THCA	1	1.007	1.349	0.349
GLCA	1.062	1.021	1.028	-0.034
GDCA	1.193	1.211	1.377	0.184
GDHCA	1.157	1.135	1.189	0.032
GHCA	1.154	1.159	1.416	0.262

c

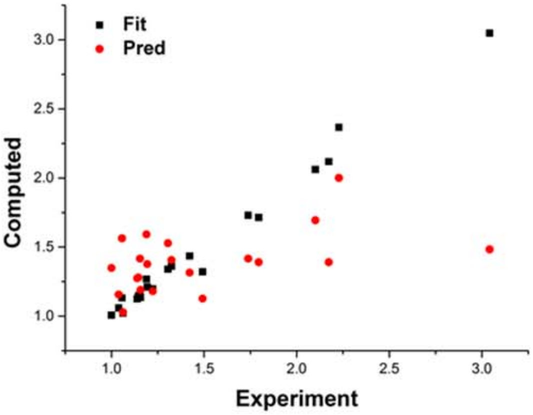

Supplemental Fig. 13. Radiometric BA transport assay in oocytes and fitted-predicted transport activities of BA. **(a)** Graphical representation of the transport activities of ^{3}H-CA, ^{3}H-DCA and ^{3}H-TCA by Δ36hENT3 expressing oocytes with respect to water-injected oocytes. Uptake was measured in sodium-free transport buffer at pH 5.5. Data represent mean±SEM (n=8 oocytes/group, *$p < 0.05$ by two-tailed t-test) **(b)** The predicted versus experimental activity of ENT3 BA transport comparison data presented. Cross-validation regression coefficient, q2 = 0.24; regression coefficient r2 = 0.98. Fit, fitted; Pred, predicted; Err Pred, error prediction. **(c)** Fitted (*black*) and predicted (*red*) transport activities for the 21 BAs in the Training Data Set. Fit, fitted; Pred, predicted.

Supplemental Figure 14

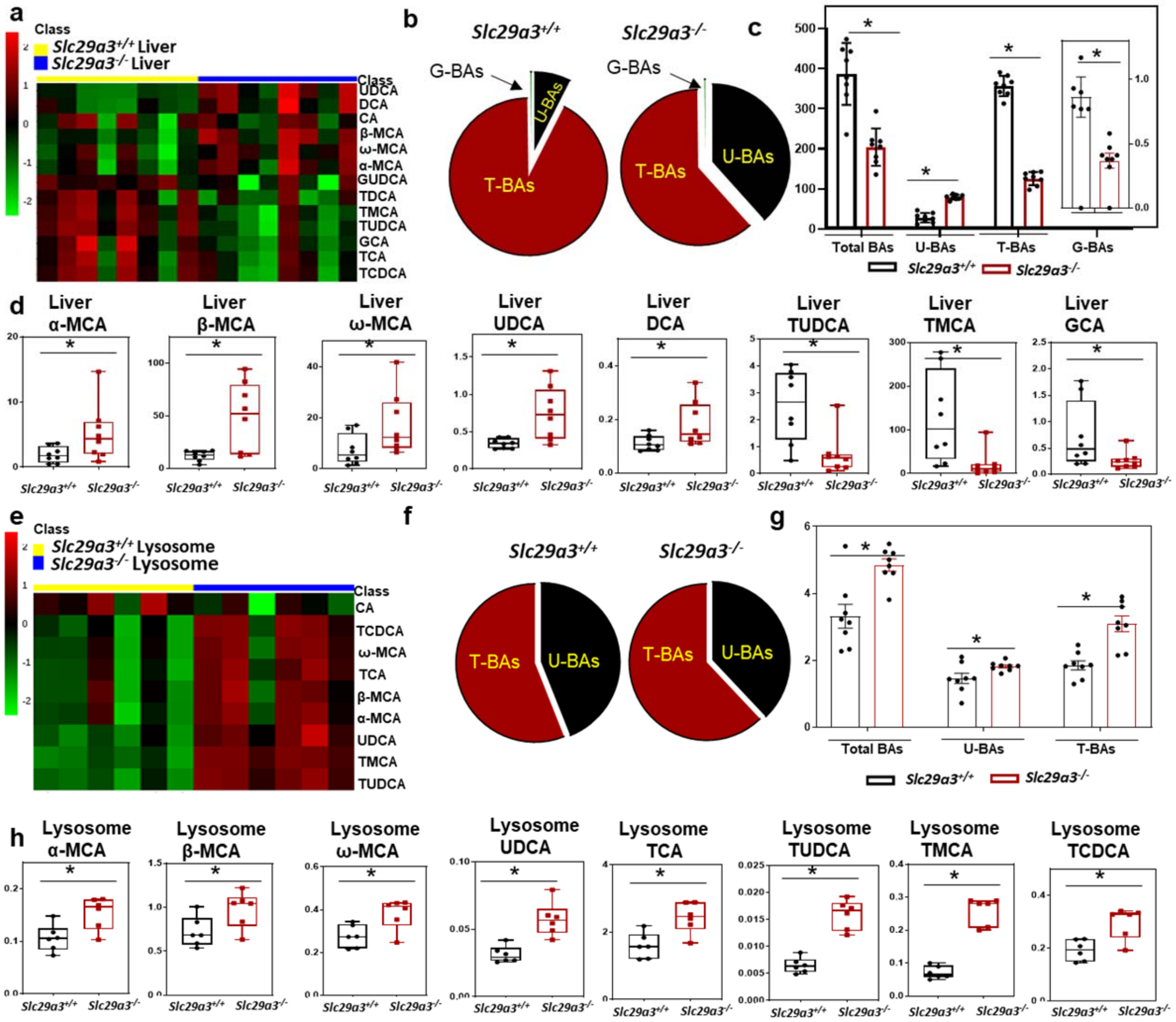

Supplemental Fig. 14. Targeted mass spectrometric analysis of *Slc29a3-/-* mouse liver and lysosomes. **(a)** Heat map representing hierarchical clustering of 13 BAs determined by targeted mass spectrometric analysis of 8 *Slc29a3*$^{-/-}$ mice liver samples. MS signal intensities were clustered in two dimensions (row represent metabolites while column indicates samples) on the basis of Euclidean distance. Colors indicate the metabolite abundances with red for high and green for low. **(b)** Pie chart illustrating the bile acid composition in *Slc29a3*$^{+/+}$ and *Slc29a3*$^{-/-}$ mice liver samples (n=8 mice/group). **(c)** The concentrations of liver total BAs, unconjugated BAs (U-BAs), taurine-conjugated BAs (TBAs) and glycine-conjugated (GBAs) as determined by LC-MS/MS analyses. Values, Mean ± S.E.M; n=8 mice/group, $p<0.05$ by two-tailed t-test comparing *Slc29a3*$^{+/+}$ and *Slc29a3*$^{-/-}$. **(d)** Box-whisker plot representation of significantly altered BAs in *Slc29a3*$^{-/-}$ mice liver samples as determined by targeted LC-MS/MS analyses. Values, Mean ± S.E.M; n=8 mice/group, $p<0.05$ versus *Slc29a3*$^{+/+}$. Box plots represent the median (middle line), 25th, and 75th percentile (box), while the whiskers span from the minimum to the maximum value. **(e)** Heat map representing hierarchical clustering of 13 bile acids determined by targeted mass spectrometric analysis of 8 *Slc29a3*$^{-/-}$ mice liver lysosome samples. MS signal intensities were clustered in two dimensions (row represent metabolites while column indicates samples) on the basis of Euclidean distance. Colors indicate the metabolite abundances with red for high and green for low. **(f)** Pie chart illustrating the bile acid composition in *Slc29a3*$^{+/+}$ and *Slc29a3*$^{-/-}$ mice liver lysosome samples (n=8 mice/group). **(g)** The concentrations of lysosome total bile acids (BAs), unconjugated BAs (U-BAs), taurine-conjugated BAs (T-BAs) and glycine-conjugated (G-BAs) as determined by LC-MS/MS analyses. Values, Mean ± S.E.M; n=8 mice/group, $p<0.05$ by two-tailed t-test comparing *Slc29a3*$^{+/+}$ and *Slc29a3*$^{-/-}$. **(h)** Box-whisker plot representation of significantly altered BAs in *Slc29a3*$^{-/-}$ mice liver samples as determined by targeted LC-MS/MS analyses. Values, Mean ± S.E.M; n=8 mice/group, $p<0.05$ by two-tailed t-test comparing *Slc29a3*$^{+/+}$ and *Slc29a3*$^{-/-}$. Box plots represent the median (middle line), 25th, and 75th percentile (box), while the whiskers span from the minimum to the maximum value.

Supplemental Figure 15

a

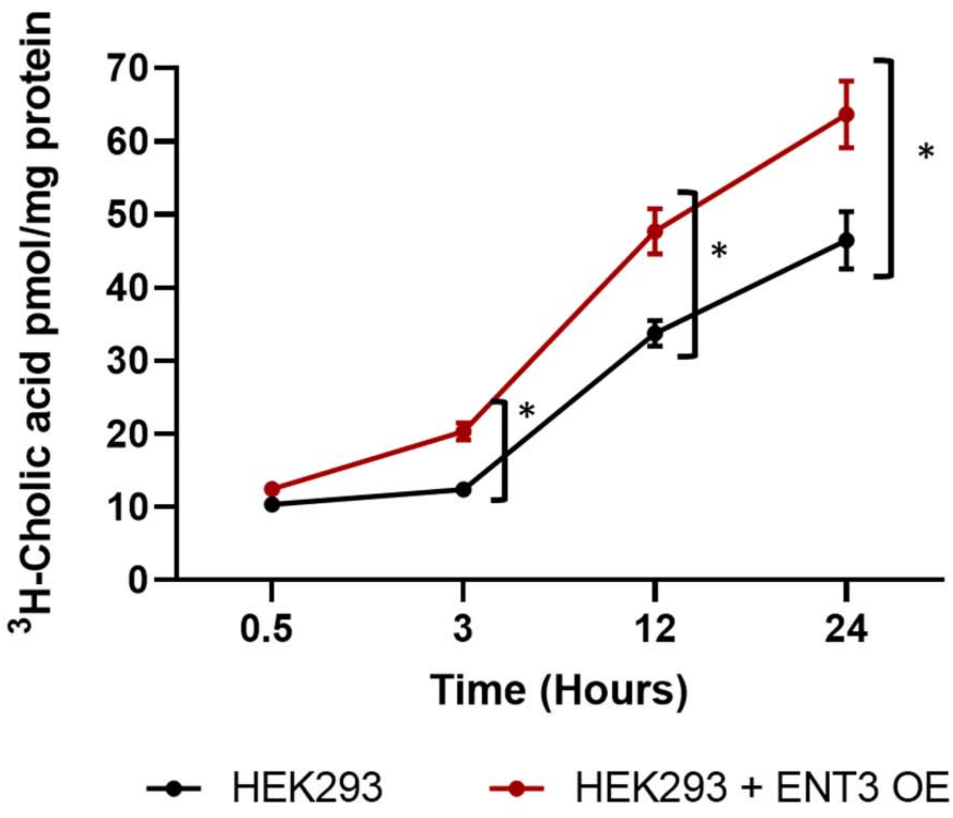

Supplemental Fig. 15. ENT3 overexpression increases BA accumulation in HEK293 cells. **(a)** Uptake of ^{3}H-cholic acid into HEK293 (control)(*black*) and HEK293 overexpressing ENT3 (HEK293 + ENT3 OE)(*red*) were measured after cells were incubated with culture media containing 1 μM cholic acid (including 0.02 μM ^{3}H-cholic acid) for 0.5, 3, 12, and 24 hours. Data represent mean±SEM (n=3 biological replicates/group, *$p < 0.05$ by two-tailed t-test).
