# Supplementary material for: Facilitative lysosomal transport of bile acids alleviates ER stress in mouse hematopoietic precursors
Source: Nat Commun. 2021 Feb 23;12:1248. doi: 10.1038/s41467-021-21451-6 (PMC7902824; doi:10.1038/s41467-021-21451-6)
Supplement: Supplementary file 2 — Reporting Summary [file 41467_2021_21451_MOESM2_ESM.pdf]

natureresearch

Corresponding Author: Rajgopal Govindarajan

Date: 23-11-2020

Life Sciences Reporting Summary

Nature Research wishes to improve the reproducibility of the work we publish. This form is published with all life science papers and is intended to promote consistency and transparency in reporting. All life sciences submissions use this form; while some list items might not apply to an individual manuscript, all fields must be completed for clarity.

For further information on the points included in this form, see Reporting Life Sciences Research. For further information on Nature Research policies, including our data availability policy, see Authors & Referees and the Editorial Policy Checklist.

Experimental design

1. Sample size

Describe how sample size was determined.

For all in vitro experiments, a sample size of n=3-12 was used based on the effect size and overlap between distributions. A power of 0.8 was set as minimal to decide the sample size for each experiment. For in vivo bone marrow transplant mice studies, sample size estimation was initially chosen by using power calculations (http://biomath.info/power/ttest.htm), and effect sizes were estimated with additional guidance from pilot studies. With alpha=0.05 and power=0.9 and allowing for unexpected mortalities of ~5%/group, ≥5 mice per group were needed for in vivo studies. A sample size of n=6 independent mice/group was selected because a definitive endpoint mortality post-irradiation was achieved in all non-transplanted mice. For in vivo studies that assessed the effects of ENT3 or bile acid treatment on survival of Slc29a3(-/-) mice, a sample size of n=6 independent mice per group was used to include inherent variabilities in survival times among Slc29a3(-/-) mice based on previous pilot studies that yielded high power (>0.8). For microarray analysis, a sample size of at least 6 independent mice/group was used base on previous experience to yield high power (>0.8) to detect differences in transporter expression, which is consistent with other published literature (PMID: 26106909, and 21455274). The sample size for the targeted metabolomics experiments was calculated based on the power analysis module in Metaboanalyst software, which uses algorithms described by van Iterson et al. 2013. The desired power of 0.8 was achieved for both the metabolomics and lipidomics data validating the sample size of n=9 independent mice per group.

2. Data exclusions

Describe any data exclusions.

No data were excluded from the analysis.

3. Replication

Describe whether the experimental findings were reliably reproduced.

All experimental findings were reliably reproduced, and attempts at replication of experimental findings were successful. Both in vitro and in vivo experiments were replicated at least three times to confirm experimental trends prior to publication.

4. Randomization

Describe how samples/organisms/participants were allocated into experimental groups.

For all studies using mice and mouse tissues, animals of either sex and similar age were randomly arranged into groups. This allocation method allowed us to generate homogeneous blocks for a randomized block design. For immunohistochemistry and clinical/anatomic pathology, the field of view was randomly selected for analysis. For flow cytometry, immunoblotting, RT-qPCR, transport studies, and metabolite profiling, samples were randomized and analyzed with standard approaches.

5. Blinding

Describe whether the investigators were blinded to group allocation during data collection and/or analysis.

Endpoint measures and outcomes for all animal experiments were objective (survival times) that did not necessitate blinding in animal experiments. For in vitro studies, the experimental groups and data collection were blind. However, the data were then analyzed without blinding since we need to know each experimental condition to analyze samples as groups.

Note: all studies involving animals and/or human research participants must disclose whether blinding and randomization were used.

6. Statistical parameters

For all figures and tables that use statistical methods, confirm that the following items are present in relevant figure legends (or the Methods section if additional space is needed).

n/a	Confirmed	
☐	☒	The exact sample size (*n*) for each experimental group/condition, given as a discrete number and unit of measurement (animals, litters, cultures, etc.)
☐	☒	A description of how samples were collected, noting whether measurements were taken from distinct samples or whether the same sample was measured repeatedly.
☐	☒	A statement indicating how many times each experiment was replicated
☐	☒	The statistical test(s) used and whether they are one- or two-sided (note: only common tests should be described solely by name; more complex techniques should be described in the Methods section)
☐	☒	A description of any assumptions or corrections, such as an adjustment for multiple comparisons
☐	☒	The test results (e.g. *p* values) given as exact values whenever possible and with confidence intervals noted
☐	☒	A summary of the descriptive statistics, including central tendency (e.g. median, mean) and variation (e.g. standard deviation, interquartile range)
☐	☒	Clearly defined error bars

See the web collection on statistics for biologists for further resources and guidance.

› Software

Policy information about availability of computer code

7. Software

Describe the software used to analyze the data in this study.

Mzmine 2.21 for mass spectrometry data deconvolution, the RANSAC algorithm for peak alignment, and Metaboanalyst 3.0 for mass spectrometry data statistical analysis were used. Graphpad Prism 7 was used for all other statistical analyses. BD FACSDIVA 8.0.1 and FlowJo 10.4.2 was used for flow cytometry data acquisition and analysis, respectively. Image Lab software 5.2.1 was used for Western blotting analysis.

For all studies, we encourage code deposition in a community repository (e.g. GitHub). Authors must make computer code available to editors and reviewers upon request. The *Nature Methods* guidance for providing algorithms and software for publication may be useful for any submission.

› Materials and reagents

Policy information about availability of materials

8. Materials availability

Indicate whether there are restrictions on availability of unique materials or if these materials are only available for distribution by a for-profit company.

No unique materials were used in the study.

9. Antibodies

Describe the antibodies used and how they were validated for use in the system under study (i.e. assay and species).

For Western blotting analysis, the following antibodies were used: Primary Antibodies - GRP78 (Abcam 108615, 1:1000) reacts with mouse and human variants and was validated by the manufacturer for use in western blotting using a series of cell lines and mouse tissues. GRP94 (Abcam 230842, 1:1000) reacts with mouse, human, and Chinease hamster variants and was validated by the manufacturer for use in western blotting using a series of cell lines and mouse tissues. p-PERK (CST 3192, 1:1000) reacts with mouse, human, rat and monkey variants and was validated by the manufacturer for use in western blotting using a series of human and mouse cell lines. eIF2a (CST 9722, 1:1000) reacts with mouse, human, rat and monkey variants and was validated by the manufacturer for use in western blotting using PC12 cells, untreated or thapsigargin-treated (300 nM) using Phospho-eIF2α (Ser51) Antibody #9721 and control eIF2α Antibody. p-eIF2a (CST 9721, 1:1000) reacts with mouse, human, rat, monkey, and D. melanogaster variants and was validated by the manufacturer for use in western blotting using PC12 cells, untreated or thapsigargin-treated (300 nM) using Phospho-eIF2α (Ser51) Antibody #9721 and control eIF2α Antibody. IRE1 (Abcam 37073, 1:1000) reacts with mouse, rat, and human variants and was validated by the manufacturer for use in western blotting using Caco-2 and SK-N-SH cell lysate. p-IRE1 (Abcam 48187, 1:1000) reacts with mouse, rat, human, and pig variants and was validated by the manufacturer for use in western blotting using HeLa cells treated with 30nM calyculin A:AP-buffer and 30nM calyculin A:Alkaline Phosphatase buffer. ATF6 (Novus Biologicals 40256, 1:1000) reacts with mouse, rat, human, porcine, plant, and rabbit variants and was validated by the manufacturer for use in western blotting using SW480 and HCT116 cells cultured in normoxia or hypoxia-like conditions. ENT3 (ThermoFisher Scientific PA5-38039,1:000) reacts with mouse, rat, and human variants and was validated by the manufacturer for use in western blotting using human bladder tissue lysate. LAMP1 (CST 3243S, 1:000) reacts with mouse, human, monkey variants and was validated by the manufacturer for use in western blotting using HeLa, Neuro2a and COS cells. GAPDH (CST 97166, 1:5000) reacts with mouse, human, monkey, and rat variants and was validated by the manufacturer for use in western blotting using various cell lines and tissues. Secondary Antibodies- Rabbit (Bethyl A120-201P,1:5,000) and mouse (Bethyl A90-116P, 1:5,000) secondary antibodies were used. Note: The source of these antibodies and catalog numbers are provided in parenthesis.

For flow cytometry analysis, the following antibodies were used: Flt3 (PE-CF594-anti-CD135, clone A2F10.1; BD B562537, 1:50 dilution) reacts with the mouse variant and was validated by the manufacturer for use in flow cytometry using a series of mouse Flt-3 transfected cells lines. IL7Ra (V450-anti-CD127, clone SB/199; BD 561205 1:50 dilution) reacts with the mouse variant and was validated by the manufacturer for use in flow cytometry using CD127 expressing BALB/c mouse splenocytes. Sca1 (PE-Cy7-anti-Ly-6A/E, clone D7; BD 558162,1:50 dilution) reacts with the mouse variant and was validated by the manufacturer for use in flow cytometry using multipotent hematopoietic stem cells (HSC) in the bone marrow of mice with both Ly-6 haplotypes. CD34 (FITC-anti-CD34, clone RAM34; BD 553733, 1:50 dilution) reacts with the mouse variant and was validated by the manufacturer for use in flow cytometry using BALB/c bone marrow cells treated with Mouse BD Fc Block™ purified anti-CD16/CD32 mAb 2.4G2 and stained with either FITC-conjugated anti-mouse CD34 (Clone RAM34) or a FITC-conjugated rat IgG2a, κ isotype control. c-kit (PE-anti-CD117, clone REA791; Miltenyi Biotech 130-122-937, 1:50 dilution) reacts with the mouse variant and was validated by the manufacturer for use in flow cytometry using mouse bone marrow suspension cells. C/EBP homologous protein (CHOP) (Alexa Fluor 647 anti-GADD153/CHOP, clone 9C8; Novus Biologicals NB600-1335AF647, 1:50) reacts with human, mouse, rat, and primate variants and is validated by the manufacturer for use in flow cytometry. Cleaved caspase-3 (FITC anti-active caspase3, clone C92-605, BD 559341, 1:50) reacts with human and mouse variants s validated by the manufacturer for use in flow cytometry using a variety of untreated and treated cell lines. Ki-67 (APC, clone SolA15; eBioscience 17-5698-82, 1:50) reacts with fish, human, and mouse variants and was validated by the manufacturer for use in flow cytometry using BALB/c splenocytes with and without stimulation.

Alternative antibody conjugates were also used in ER stress flow cytometry panels: CD34 (Alexa Fluor 700-anti-CD34, clone RAM34; BD 560518, 1:50 dilution) reacts with the mouse variant and was validated by the manufacturer for use in flow cytometry using BALB/c bone marrow cells treated with Mouse BD Fc Block™ (Purified Rat Anti-Mouse CD16/CD32 mAb 2.4G2 (Cat. No. 553141)) and stained with either the Alexa Fluor® 700 Rat Anti-Mouse CD34 antibody or a Alexa Fluor® 700 Rat IgG2a, κ isotype control. IL7Ra (PE-CF594-anti-CD127, clone SB/199; BD 562419, 1:50 dilution) reacts with the mouse variant and was validated by the manufacturer for use in flow cytometry using CD127 expressing BALB/c mouse splenocytes. Flt3 (APC-anti-CD135, clone A2F10.1; BD 560718, 1:50 dilution) reacts with the mouse variant and was validated by the manufacturer for use in flow cytometry using bone marrow cells from BALB/c mice stained with FITC Rat Anti-Mouse CD11b (Cat. No. 553310) and FITC Rat Anti-Mouse CD45R/B220 (Cat. No. 553088) in conjunction with either a APC Rat IgG2a, κ isotype control or with the APC Rat Anti-Mouse CD135 antibody. The Lineage Cell Detection Cocktail-Biotin (Miltenyi Biotech 130-092-613, 1:10 dilution) reacts with the mouse lineage cells and was validated by the manufacturer for use in flow cytometry using mouse bone marrow cells. The anti-Biotin secondary antibody (PerCP-Vio700-anti-Biotin, clone Bio3-18E7; Miltenyi Biotech 130-113-856,1:50 dilution) reacts with biotinylated markers and was validated by the manufacturer for use in flow cytometry using PBMCs labeled with biotin.

Unconjugated ER stress antibodies were also used:p-eI2Fa (anti-p-eIF2a, clone EPR11042; Abcam 169528,1:20 dilution) reacts with mouse, human, and rat variants and was validated by the manufacturer for use in western blots, immunohistochemistry, and flow cytometry using several animal tissues. p-PERK (anti-p-PERK, clone Thr980; ThermoFisher Scientific MA5-15033, 1:20 dilution) reacts with human and mouse variants and have been validated for western blot and flow cytometry in RSC96 cells treated with thapsigargin and tunicamycin. GRP78 (anti-GR78/BIP, clone EPR4041 (2); Abcam 108615, 1:20 dilution) reacts with human and mouse variants and have been validated for western blot and flow cytometry in cell lines as well as human and mouse tissue lysates. GRP94 (anti-GRP94, clone EPR22847-50; Abcam 238126, 1:20 dilution) reacts with human, mouse, and rat variants and have been validated for western blot and flow cytometry in HEK293T and HeLa cell lysates. Cleaved caspase-3 (anti-cleaved-caspase-3, clone 299518; R&D Systems MAB835, 1:20 dilution) reacts with human and mouse variants and have been validated for western blot and flow cytometry in Jurkat human acute T cell leukemia cell line and DA3 mouse myeloma cell line untreated or treated with 1 μM staurosporine.

Stem cell transplantation flow cytometry was performed using: Mouse lineage antibody cocktail (APC anti-lineage; BD 558074, 1:5 dilution) reacts with mouse lineage cells and was validated for use in flow cytometry using BALB/c bone marrow cells were treated with Mouse BD Fc Block™ Purified anti-CD16/CD32 (Cat. No. 553141/553142), stained with FITC Rat anti-Mouse CD34 (Cat. No. 553733) and either APC Mouse Lineage Isotype Control Cocktail or APC Mouse Lineage Antibody Cocktail. Sca1 (BUV395 anti-Sca1, clone D7; BD 563990, dilution 1:50) reacts with the mouse variant and has been validated for flow cytometry in mouse splenocytes. Sca1 (APC/Cy7 anti-Sca1, clone D7; BioLegend 108125, 1:50 dilution) reacts with the mouse variant and has been validated for flow cytometry in C57BL/6 mouse splenocytes. CD117 (PE-Cy7 anti-CD117, clone 2B8; BD 558163, 1:50 dilution) reacts with the mouse variant and has been validated for flow cytometry in C57BL/6 mouse bone marrow cells. CD34 (PerCP/Cy5.5 anti-CD34, clone HM34; BioLegend 128608, 1:50 dilution) reacts with the mouse variant and has been validated for flow cytometry in mouse fibroblast NIH/3T3 cells. Flt3 (PE anti-CD135, clone A2F10.1; BD 553842, 1: 50 dilution) reacts with the mouse variant and has been validated for flow cytometry in mouse splenocytes. CD71 (PerCP/Cy5.5 anti-CD71, clone C2; BD 562858, 1:50 dilution) reacts with mouse variant and has been validated for flow cytometry in BALB/c mouse bone marrow cells.

TER119 (Biotin Anti-mouse TER119, clone TER-119; BioLegend 116204, 1:50 dilution) reacts with mouse variant and has been validated for flow cytometry in BALB/c mouse bone marrow cells. Streptavidin (APC anti-streptavidin, Biolegend 405243, 1:5000 dilution) reacts with human, mouse, and rat has been validated for IHC and flow cytometry in conjunction with biotinylated primary antibodies.

10. Eukaryotic cell lines

a. State the source of each eukaryotic cell line used.

Retroviral (ATCC, CRL 9078) and lentiviral packaging (ATCC, CRL 11268) cell lines and HEK 293 (ATCC, CRL 1573) were purchased from American Type Culture Collection (ATCC, Manassas). These cell lines were propagated, expanded, and frozen immediately upon receipt. The cells revived from the frozen stock were used within 10-20 passages, not exceeding a period of 2-3 months.

b. Describe the method of cell line authentication used.

The ATCC uses morphological, cytogenetic, and DNA profile analyses for the characterization of cell lines.

c. Report whether the cell lines were tested for mycoplasma contamination.

All cell lines tested negative for mycoplasma contamination. Last testing date: September 2020.

d. If any of the cell lines used in the paper are listed in the database of commonly misidentified cell lines maintained by ICLAC, provide a scientific rationale for their use.

No commonly misidentified cell lines were used in the study.

Animals and human research participants

Policy information about studies involving animals; when reporting animal research, follow the ARRIVE guidelines

11. Description of research animals

Provide details on animals and/or animal-derived materials used in the study.

Mice of 2-20 weeks from either sex from mixed background of 129S5/ SvEvBrd and C57BL6/J were used for the study. Mouse plasma, urine and liver were used for untargeted and targeted metabolomics and mouse bone marrow was used for flow cytometry, histopathology, biochemical and molecular studies. All animal studies were performed in accordance with Animal Care and Use Programs under protocols approved by the Institutional Animal Care and Use Committee at OSU. We have complied with the relevant ethical considerations for animal research overseen by this committee. Mice were housed in a conventional animal facility with an ambient temperature and humidity of 20-26C and 40%-60%, respectively.

Policy information about studies involving human research participants

12. Description of human research participants

Describe the covariate-relevant population characteristics of the human research participants.

The study did not involve human research participants.
