# Supplementary material for: Facilitative lysosomal transport of bile acids alleviates ER stress in mouse hematopoietic precursors
Source: Nat Commun. 2021 Feb 23;12:1248. doi: 10.1038/s41467-021-21451-6 (PMC7902824; doi:10.1038/s41467-021-21451-6)
Supplement: Supplementary file 3 — Description of Additional Supplementary Files [file 41467_2021_21451_MOESM3_ESM.pdf]

Description of Additional Supplementary Files

File Name: Supplementary Data 1

Description: Primer pairs used for PCR assay in the study

File Name: Supplementary Data 2

Description: Table representing normalized peak intensities, fold change, p-value and VIP score for all the detected features in untargeted profiling of *Slc29a3*$^{-/-}$ plasma samples. List of differential metabolites detected in the plasma from *Slc29a3*$^{+/+}$ and *Slc29a3*$^{-/-}$ mice (12 weeks age) based on a p-value < 0.05 using a one-sided t-test and a fold-change > 2.0. The m/z and ionization mode (positive ([M+H]+) or negative ([M-H]-)) are mentioned for all the detected metabolites. In addition to the fold change, the log2-fold change, p-value, –log 10 (p) and VIP score for each compound are indicated. The chemically annotated metabolites have been categorized according to their superclass—i.e., lipids ; amino and organic acids; carbohydrates; nucleosides, nucleotides, and analogues; organoheterocyclic compounds and benzenoids.

File Name: Supplementary Data 3

Description: Table representing normalized peak intensities, fold change, p-value and VIP score for all the detected features in untargeted profiling of *Slc29A3*$^{-/-}$ urine samples. List of differential metabolites detected in the urine from *Slc29a3*$^{+/+}$ and *Slc29a3*$^{-/-}$ mice (12 weeks age) based on a p-value < 0.05 calculated using a one-sided t-test and a fold-change > 2.0. The m/z and ionization mode (positive ([M+H]+) or negative ([M-H]-)) are mentioned for all the detected metabolites. In addition to the fold change, the log2-fold change, p-value, –log 10 (p) and VIP score for each compound are indicated. The chemically annotated metabolites have been categorized according to their superclass—i.e., lipids ; amino and organic acids; carbohydrates; nucleosides, nucleotides, and analogues; organoheterocyclic compounds and benzenoids.
